# 3D anatomical atlas of the heads of male and female adult *Chamaeleo calyptratus*

**DOI:** 10.1002/ar.70077

**Published:** 2025-11-15

**Authors:** Alice Leavey, Eloy Gálvez‐López, Anthony Herrel, Laura B. Porro

**Affiliations:** ^1^ Centre for Integrative Anatomy, Cell and Developmental Biology University College London London UK; ^2^ Mécanismes Adaptatifs et Evolution, UMR 7179 Muséum National d'Histoire naturelle CNRS Paris France; ^3^ Department of Biology, Evolutionary Morphology of Vertebrates Ghent University Ghent Belgium; ^4^ Functional Morphology Laboratory, Department of Biology University of Antwerp Antwerp Belgium; ^5^ Naturhistorisches Museum Bern Bern Switzerland

**Keywords:** deep learning, digital dissection, education, model organism, sexual dimorphism, Squamata, veiled chameleon

## Abstract

The veiled chameleon, *Chamaeleo calyptratus,* is a model organism in the study of reptile development. The extreme size of its cranial casque relative to other chameleons has been linked to high bite forces and conspecific signaling, yet its unique head anatomy has not been described. In general, detailed accounts of chameleon cranial musculature are scarce and not in formats that are easily accessible for use by researchers and educators. In this study, we provide the first complete description of both the cranial osteology and soft tissues in two adult specimens, exploring differences between sexes. Utilizing contrast‐enhancing staining techniques, microCT scanning and deep learning models for segmentation, we have generated the first digital anatomical atlases of the chameleon skull, nervous system, cranial muscles and, in the female, hyolingual muscles. This includes the first maps of chameleon cranial sutures and muscle attachment sites. Our results show that the sexes differ most in the structure of the facial skeleton and the enlarged cranial casque. Many osteological differences are reflected in muscle topology and size, where the male presents a considerably larger jaw adductor. These atlases represent novel insights and potential applications for future research and education in reptile evolution and development. As an example, we have published a series of lesson plans alongside our atlases to help educators bridge the gap between theoretical knowledge and hands‐on analysis in comparative anatomy.

## Graphical abstract

The veiled chameleon is a model organism in reptile development research. Utilizing contrast‐enhanced microCT and deep learning segmentation models, we have generated the first digital atlases of the skull, nervous system, cranial muscles and hyolingual muscles. This includes the first maps of cranial sutures and muscle attachment sites in chameleons.

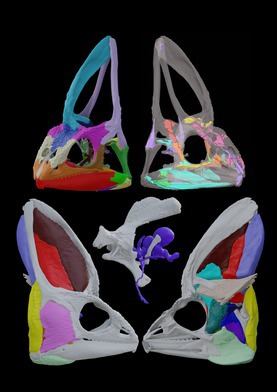

## 1 INTRODUCTION

Reptiles have long been valued in comparative anatomy because their extraordinary morphological and developmental diversity offers critical insights into both conserved vertebrate traits and lineage‐specific innovations. However, their potential in developmental studies has often been limited by the fact that most reptile embryos are deposited at relatively advanced stages, restricting access to the earliest phases of embryogenesis (Diaz Jr et al., 2015). Recently, the veiled chameleon, *Chamaeleo calyptratus*, has been adopted as a model system for the study of reptile development, evolution, and ecological niche specialization (Diaz Jr et al., 2015). For example, *C. calyptratus* has previously been used to study embryonic development (Andrews & Donoghue, 2004; Buchtová et al., 2013; Diaz Jr et al., 2019, 2022), disease (Hoby et al., 2010; Paré et al., 2006), sexual dimorphism (Bauerová et al., 2020; Vanhooydonck et al., 2007), social behavior (Ballen et al., 2014; Drown et al., 2022), communication (Denny et al., 2023; Ligon & McGraw, 2018; Tegge et al., 2020), phenotypic plasticity (Andrews, 2018), feeding mechanics (Herrel et al., 2014), and climbing mechanics (Krause & Fischer, 2013; Luger et al., 2021). Several factors have prompted this promotion to model organism. Unlike most other squamate reptiles, several chameleon species present an embryonic diapause shortly after fecundation, leading to its development at the time of oviposition being only at the early gastrula stage (Andrews & Donoghue, 2004; Measey et al., 2014). Thus, females do not need to be sacrificed in order to study early embryonic stages (Diaz Jr et al., 2017). They also display rapid growth within the first year of life (Karsten et al., 2008). Veiled chameleons have simple husbandry requirements, large clutch sizes, and relatively short life spans compared to other reptiles (Molnar et al., 2017). Furthermore, this species could be an excellent model for understanding how multiple factors influence skull morphology. Habitat or intraspecific aggression and communication have been linked to differences in chameleon skull morphology in general (Measey et al., 2009; Stuart‐Fox et al., 2006), and in veiled chameleons in particular (Ligon & McGraw, 2018). Veiled chameleons are also sexually dimorphic in both body and casque size (Bauerová et al., 2020; Vanhooydonck et al., 2007), which has been linked to differences in skull morphology in other chameleon species (Dollion et al., 2017; da Silva et al., 2014). Changes in veiled chameleon skull morphology—particularly the enlargement of their distinctive casque—likely permit changes in the size and orientation of the jaw muscles, which in turn could facilitate dietary differences among age groups and between dimorphic males and females. For example, some dietary differences between sexes have been described for *Bradypodion* species (da Silva et al., 2016, but cf. Measey et al., 2011). Detailed descriptions of the structure, orientation, and distribution of the cranial muscles in *C. calyptratus* would allow future studies to be better equipped to address how structural variation is linked to biomechanical function, behavior, and ecology in chameleons.

Despite this, there is a lack of comprehensive anatomical descriptions for *C. calyptratus*. Skeletal anatomy has been described for other species of *Chamaeleo* (Methuen & Hewitt, 1914; Parker, 1881) and other chameleon genera (see Anderson & Higham, 2014 for a review), but the only studies which include *C. calyptratus* focus on the vertebral atlas (Čerňanský et al., 2014) and teeth (Kavková et al., 2022). Regarding soft tissue anatomy, there is a lack of literature for chameleons in general (Anderson & Higham, 2014; Iordansky, 2016). Recent research has explored the unique structure of the veiled chameleon tail (Luger et al., 2021) and limbs (Molnar et al., 2017), but information on cranial anatomy is scarce compared to other reptiles (Banzato et al., 2012; Callahan et al., 2021; Daza et al., 2011; Holliday et al., 2022). Detailed anatomical descriptions tend to focus on feeding, as chameleon tongues can rapidly protract over a distance exceeding the length of the chameleon's body during prey capture (Gnanamuthu, 1930; Herrel et al., 2001). For example, Iordansky (2016) describes specific elements of the musculature and ligaments associated with the jaw apparatus for four other species of *Chamaeleo* (as well as two *Trioceros* species, mistakenly attributed to *Chamaeleo*; Tilbury & Tolley, 2009). For *C. calyptratus*, the hyolingual apparatus has been described (Herrel et al., 2001), which is the only anatomical description of any cranial musculature in this species to date. Furthermore, no studies have explored whether the considerably larger casques in males relative to females (Bauerová et al., 2020; Kelso & Verrell, 2002) result in sexual dimorphism of cranial muscle anatomy.

Most anatomical descriptions of chameleons are sourced from physical dissections (Iordansky, 2016; Rieppel, 1981, 1987), but there are several limitations to this approach. The damage caused by gross dissection makes data collection unrepeatable and destroys the 3D topology of muscles. This can make it difficult to visualize muscle pathways, attachment sites, and fiber architecture, which can impact functional analyses and biomechanical models (Gröning et al., 2013). Microcomputed tomography has facilitated a non‐invasive approach to examine chameleon skeletal (Čerňanský et al., 2014; Kavková et al., 2022; Maisano, 2003) and soft tissue anatomy (Luger et al., 2021; Molnar et al., 2017). Staining specimens with contrast agents increases the radiopacity of soft tissues so they are visible through microCT scanning (see Koç et al., 2019 for a review). Phosphomolybdic acid (PMA) binds to phospholipids and collagen, allowing the visualization of not only muscles, but also tendons and ligaments (Balint et al., 2016; Descamps et al., 2014; Nierenberger et al., 2015; Pauwels et al., 2013). Previously, PMA has been used to visualize and analyze the soft tissues of marine invertebrates (Eggermont et al., 2020; Golding et al., 2009; Golding & Jones, 2007; Roscian et al., 2023), fish (Aiello et al., 2023; Brocklehurst et al., 2019; Criswell et al., 2017; Kague et al., 2019; Neutens, 2016), mammals (Disney et al., 2017; Nieminen et al., 2015), amphibians (Lowie et al., 2022, 2023), and reptiles (Baeckens et al., 2017; Luger et al., 2021; Rajabizadeh et al., 2021).

This paper aims to provide the first in‐depth description of the cranial musculoskeletal anatomy of adult *C. calyptratus* in both a male and female specimen. We discuss how major anatomical features, such as bone contacts and muscle topology, vary between sexes, as well as how they could be involved in function in this emerging model organism. Finally, 3D digital atlases and virtual dissection lesson plans aimed at GCSE and university undergraduate students are published alongside this manuscript for both specimens, facilitating future research and education on reptile cranial anatomy.

## 2 METHODS

### 2.1 Specimen staining and μCT scanning

Using the HECTOR system at the μCT scanning facilities of UGCT (Ghent University, Belgium), scans were generated for the heads of two adult specimens of similar age—one male (mandible length: 43.93 mm) and one female (mandible length: 33.77 mm). Scans of the unstained specimens were performed first to capture skeletal morphology in high resolution (100 kV, 40 W, 1801 projections, 500 ms exposure time, 1 mm Al filter). As these specimens had been fixed and stored in 70% ethanol, they were placed in 50% ethanol then in 30% ethanol for 24 h each prior to staining to minimize osmotic shock. The specimens were stained in 2.5% PMA for approximately 3 weeks and scanned again (100 kV, 40 W, 1801 projections, 500 ms exposure time, no filter). All original CT data generated by this study are available on MorphoSource under the project name “Veiled chameleon atlas” (https://www.morphosource.org/projects/000752255?locale=en).

### 2.2 Digital dissection

First, the unstained scans were registered and aligned with those of their respective stained scan, allowing the incorporation of our skull segmentation into the muscle segmentation. Then, the skulls were digitally segmented from the unstained scans. For the male specimen, imaging data was segmented using Dragonfly 3D World (Version 2024.1). The skull was separated from the surrounding media using the “split at Otsu” function, which creates two distinct regions of interest (ROIs) by separating the voxels into two classes: a lighter “foreground,” and a darker “background.” This separation is achieved by minimizing intra‐class intensity variance and maximizing inter‐class variance (Otsu, 1975). The function was applied twice, once to separate the specimen from the background, and again to separate bone from soft tissues. Then, the individual bones were separated using a combination of paintbrush tools and ROI operations (e.g., subtraction, island processing). For the female specimen, we used a new automated add‐on for Amira (Version 2020.2) called BounTI (Didziokas et al., 2024) to perform an initial segmentation of each individual bone before finishing the segmentation manually using thresholding, paintbrush and “magic wand” tools.

Soft tissue dissection for both specimens took place in Dragonfly 3D World using its deep learning tool, which required the creation of a training dataset. To do that, the “split at Otsu” function was first used to separate the background from the specimen. Then, the bone ROI from the unstained scan segmentation was imported in and subtracted from the specimen, followed by “split at Otsu” again to separate the muscle (which generally appears lighter in the scan) from other soft tissues. This generated preliminary ROIs for the four key materials in our training dataset: background, bone, muscle, and other soft tissue. Second, we selected large areas in five slices spread across the scan using the square drawing tool to create a “mask” ROI. These areas were selected to showcase the existing variability within our materials. The intersects between this “mask” and each of our four key materials were then computed and manually refined using paintbrush tools to move voxels between materials. The resulting materials were combined into a single multi‐ROI, which represents the “ground truth” data for training the deep learning neural networks.

After some preliminary tests, the deep learning architecture that resulted in the most accurate separation of our four materials was the UNet3D model for semantic segmentation, which is specifically designed for biomedical imaging data (Ronneberger et al., 2015). To train the UNet3D model, data were augmented 10 times using Dragonfly's default settings—a random combination of horizontal and vertical flipping, rotating by up to 180°, shearing by up to 2°, and scaling between 75% and 125%. The training was set up for 100 epochs but the loss function stabilized (i.e., did not decrease any further for 15 consecutive epochs) by epoch 20 for both specimens. We repeated this process over five cycles, as this was the point by which the validated loss no longer decreased during training. Once this training phase was complete, we applied the model to the entire dataset. The resulting multi‐ROI had separated our four key materials more accurately than using just the “Split at Otsu” function, which better facilitated the manual separation of each muscle body using paintbrush tools and ROI operations. Individual muscles and their respective bundles were identified and separated based on visual differences in the density between tissues, muscle fiber orientation, and muscle topology. Muscles were digitally dissected on the right side of the male specimen and on the left side of the female specimen. Finally, the sutures between bones and the muscle attachment sites were visualized by dilating each bone and muscle once, and then creating a new ROI from the intersect between them.

STL meshes for each bone, muscle, suture, and muscle attachment site were then exported. Blender (Version 4.2) was used to smooth each material for visualization and figure creation. The resulting 3D anatomical atlases are available to view on SketchFab (https://sketchfab.com/aleavey/collections) and download from Figshare (https://figshare.com/projects/3D_anatomical_atlas_of_the_heads_of_male_and_female_adult_Chamaeleo_calyptratus/263071). Lesson plans aligned with the UK science curriculum for GCSE level and first‐year undergraduate students have been included in the Supplementary Information to facilitate the use of these atlases.

### 2.3 Calculating muscle and bite forces, and mechanical advantage

To estimate muscle forces, bite forces, and mechanical advantage for our specimens, we used information from digital dissections and 3D lever models (Davis et al., 2010). Individual muscle volumes were measured in Dragonfly 3D World and total muscle lengths (centroid to centroid distance between attachment sites) were calculated in Amira from the exported STL meshes. The XYZ coordinates of muscle attachment site centroids were identified using the Matlab script Area_Centroids_From_ STL (Davis et al., 2010). As we did not measure fiber lengths in this study, all muscles were modeled to have fiber lengths that were two‐thirds the length of the muscle following Bates and Falkingham (2018) who found this value is generally conserved across vertebrates. Muscle force for parallel‐fibred muscles was estimated using Equation (1) (Sacks & Roy, 1982):

(1) $$\text{Force}=\frac{\text{Volume}}{\mathrm{Lm}}\times \text{Specific tension}$$

In which *Lm* is muscle length and specific tension of vertebrate striated muscle is 30 Ncm^**−**2^ (Hieronymus, 2006). It should be noted that we calculated anatomical cross‐sectional area for muscles rather than physiological cross‐sectional area as we did not account for pennation. See Section 4.4 for further discussion and the Supplementary Dataset for the cross‐sectional areas and estimated maximal force for each muscle.

3D moment arm lengths were calculated as the perpendicular distance from the jaw joint to the vector of the muscle force, the latter being a line between the XYZ coordinates of the centroids of the muscle origins and insertions. Muscle forces from both sides of the skull were used to estimate unilateral bite forces at three different points along the tooth row: we selected the most anterior tooth, a middle tooth (~50% of the tooth row length), and the most posterior tooth. 3D distances were measured from the jaw joint to the tip of these teeth in Amira. Only force from the jaw‐closing muscles was used in estimating bite force, which was calculated using Equation (2):

(2) $$\text{Bite Force}=\frac{\left(\sum \text{Muscle Forces}*\text{Muscle Moment Arms}\right)}{\text{Bite Point Moment}\;\mathrm{Arm}}$$

The estimated bite force was divided by the input muscle forces to estimate mechanical advantage along the tooth row, which is a measure of how efficiently skull geometry converts input muscle force into output bite force.

As the male skull is larger than that of the female, we also calculated volumes, muscle lengths, cross‐sectional areas and resulting forces with the female skull scaled to the same head width as the male, following findings from Measey et al. (2009) that bite force is best predicted by skull width. This allowed us to look at the impact of skull shape on bite force independent of skull size.

## 3 RESULTS

### 3.1 Skull

Here, we provide the first detailed anatomical description of the cranial skeleton for *C. calyptratus*, including the first detailed discussion of sutures in chameleons. This study focuses on the male specimen in detail and notes anatomical variations present in the female specimen (summarized in Table 1). The bone nomenclature and suture terminology used in this study are consistent with previous literature (Anderson & Higham, 2014; Jones et al., 2011; Rieppel, 1981, 1987; Villa & Delfino, 2019). While the digital atlas of the female specimen also includes the tongue skeleton (Figure S1), we do not describe it here as it has already been extensively described (see Herrel et al., 2001).

**TABLE 1:** Summary of *Chamaeleo calyptratus* skull morphology, color‐coded to match Figures 1 and 2.

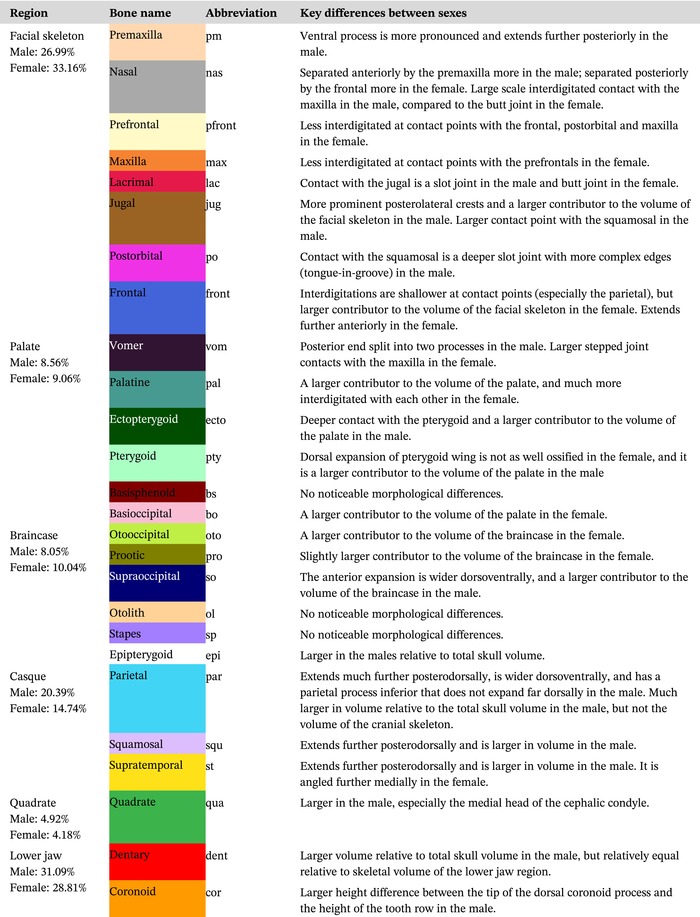
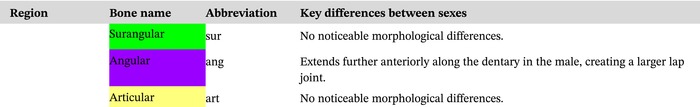

*Note*: The percentages in the first column represent the total regional muscle volume relative to skull muscle volume. For quantitative comparisons of the size differences for each bone, see Figure 5.

**FIGURE 1:**
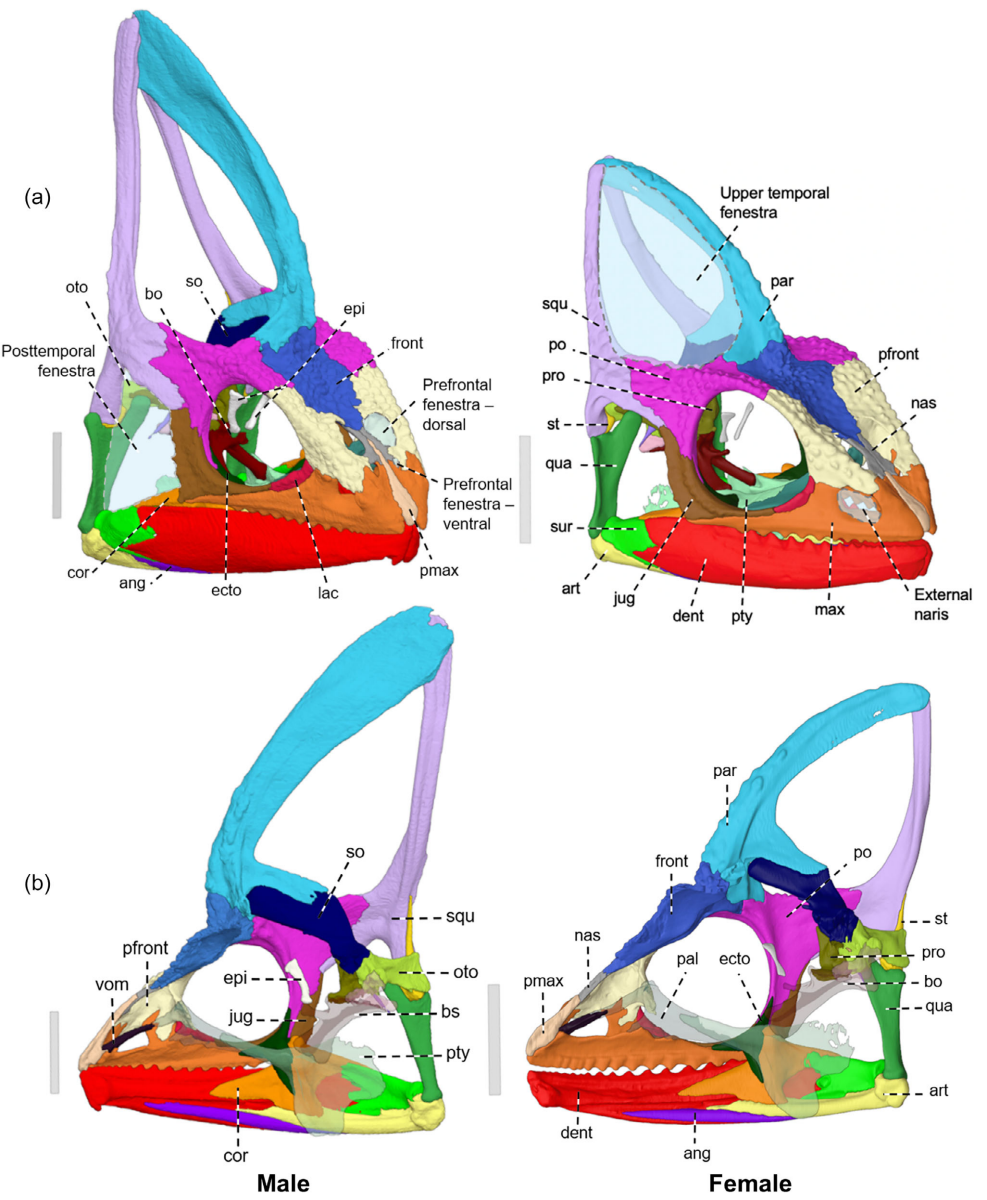
3D digital reconstruction of the skull of *Chamaeleo calyptratus*, visualized in Blender (Version 4.2) in a) anterolateral and b) medial views. The female (right) has been scaled to the same mandibular length as the male (left). The gray scale bars next to each figure are 10 mm in length. The palatine, pterygoid, basisphenoid and basioccipital have been made transparent in the medial view. See Table 1 for the full names of each abbreviated label.

**FIGURE 2:**
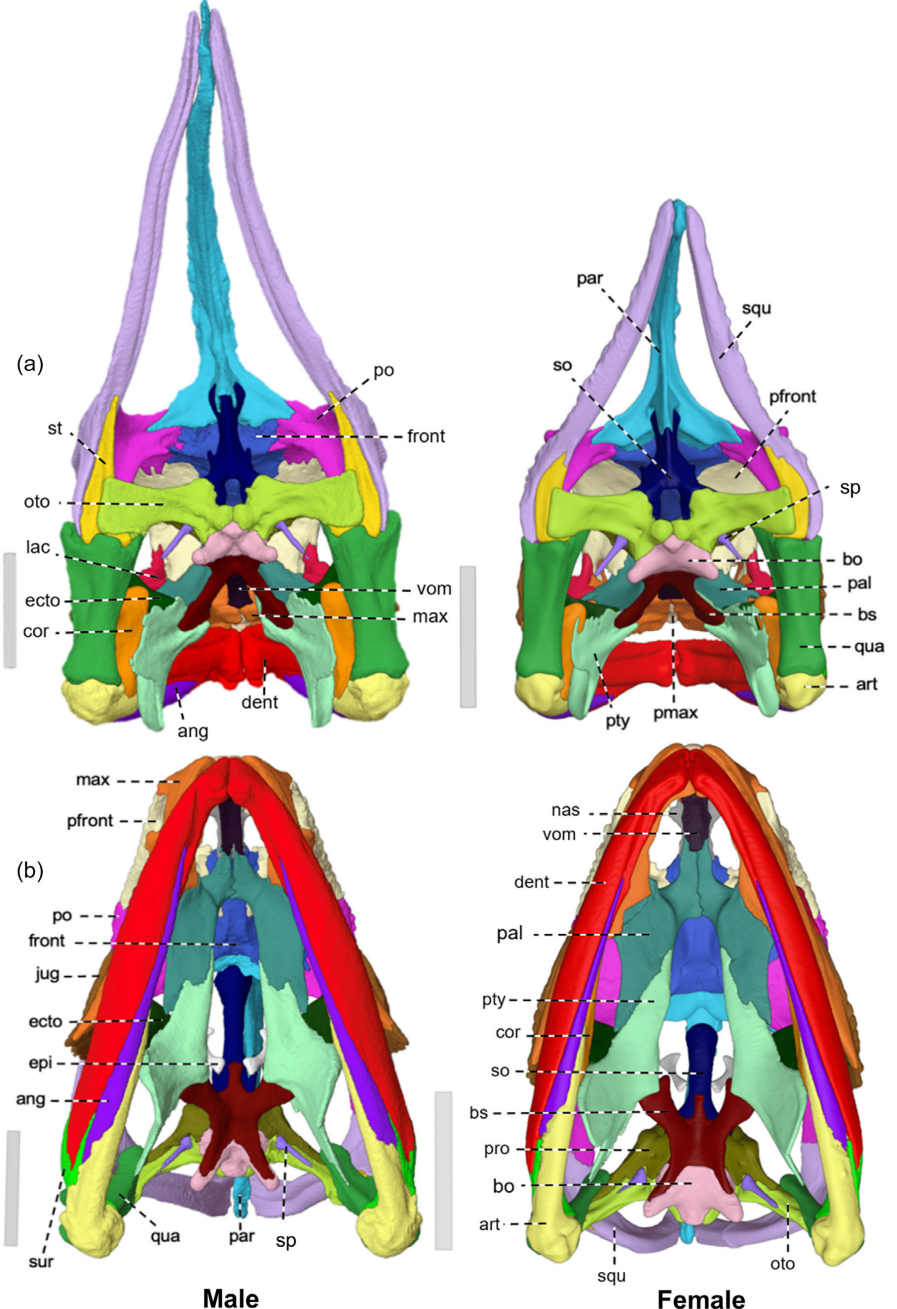
3D digital reconstruction of the skull of *Chamaeleo calyptratus*, visualized in Blender (Version 4.2) in (a) posterior and (b) ventral views. The female (right) has been scaled to the same mandibular length as the male (left). The gray scale bars next to each figure are 10 mm in length. See Table 1 for the full names of each abbreviated label.

**FIGURE 3:**
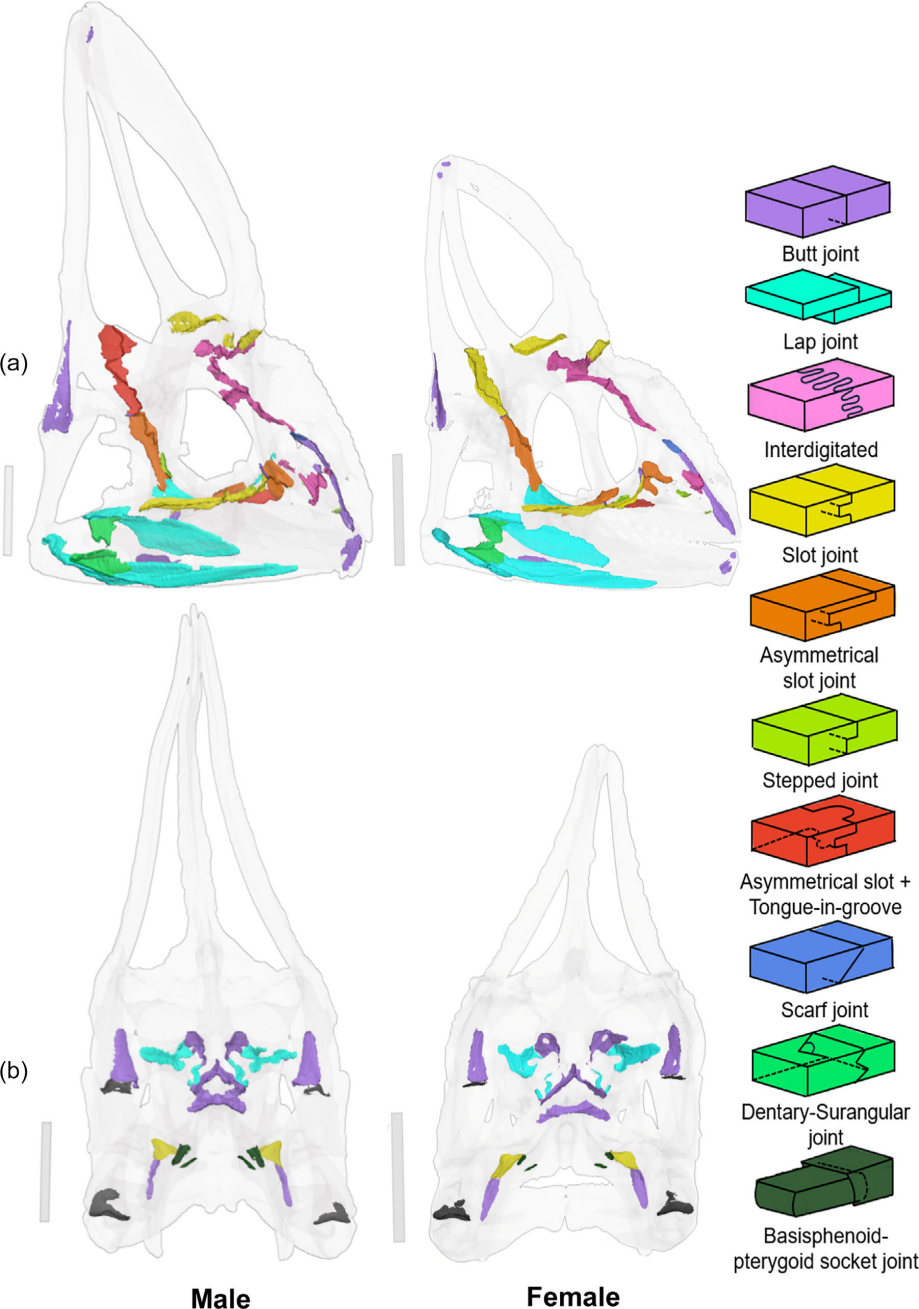
Map of the sutures in the skull of *Chamaeleo calyptratus*, visualized in Blender (Version 4.2). The female (right) has been scaled to the same mandibular length as the male (left). The anterolateral view (a) shows the sutures of the facial skeleton, casque, lower jaw, and some of the palate on the right side of the skull. The posterior view (b) shows sutures of the braincase and the posterior end of the palate. The key shows how each suture type is color‐coded and is ordered from most to least frequent suture type. The black contacts shown for the quadrate (b) represents its synovial joints, rather than a suture. The gray scale bars next to each figure are 10 mm in length.

The overall profile of the skull in *C. calyptratus* is dominated by the presence of a dorsally expanded casque as part of the cranial skeleton—the largest among known chameleon species (Measey et al., 2009; AH, personal observation)—especially in the male (Table 2; Figures 1, 2, 3; Bauerová et al., 2020). The skull of chameleons can be divided into five major regions—the facial skeleton, palate, braincase, casque, and lower jaw—plus the quadrates, which connect the cranium to the mandible. Each region is described in detail below, and our findings indicate that the total volume of bone in each region relative to total skull volume varies between males and females (Table 1).

**TABLE 2:** Descriptive ratios from the skull of each specimen, extracted from Dragonfly 3D World using the 3D line tool.

Measurements	Male	Female
Basal skull length: Mandible length	0.888	0.948
Skull width: Mandible length	0.448	0.472
Total skull height: Mandible length	1.46	1.128
Casque height: Total skull height	0.599	0.484
Tooth row length: Mandible length	0.466	0.532
Orbit diameter: Mandible length	0.315	0.362
Prefrontal fenestra (dorsal) diameter: Mandible length	0.124	0.167
Prefrontal fenestra (ventral) diameter: Mandible length	0.043	0.039
External naris diameter: Mandible length	0.1	0.133
Nasolacrimal canal diameter: Mandible length	0.051	0.073

*Note*: Diameters were measured at the widest point. All raw data, including descriptions of each measurement, can be found in the Supplementary dataset.

As with most lepidosaurs, the skull in *C. calyptratus* is highly fenestrated. The upper temporal fenestra is one of the largest openings in the skull due to the highly developed casque (Figure 1a). As in other species of chameleon (Anderson & Higham, 2014), the orbit and posttemporal fenestra are both enlarged (Figure 1). While the prefrontal fenestra is not connected to the external naris, as in many extant chameleons (Čerňanský et al., 2020), we find that this fenestra is divided in two in these specimens. In some adult specimens the smaller ventral division is completely closed (EGL, pers. obs.). The maximum diameter of the dorsal division is larger in the female, as is the maximum diameter of the external naris (both relative to mandible length; Table 2).

As with other species of chameleon, the position of the jaw joint is depressed relative to the tooth row, and our specimens have acrodont teeth that are firmly connected to the dentary and maxilla (Figure 1a; Kavková et al., 2022). The mandibular adductor fossa is continuous with the medial side of the dentary, and there is an opening to the mandibular canal positioned between the posterior end of the dentary and the anterior end of the surangular on the medial aspect.

#### 3.1.1 Facial skeleton

**FIGURE 4:**
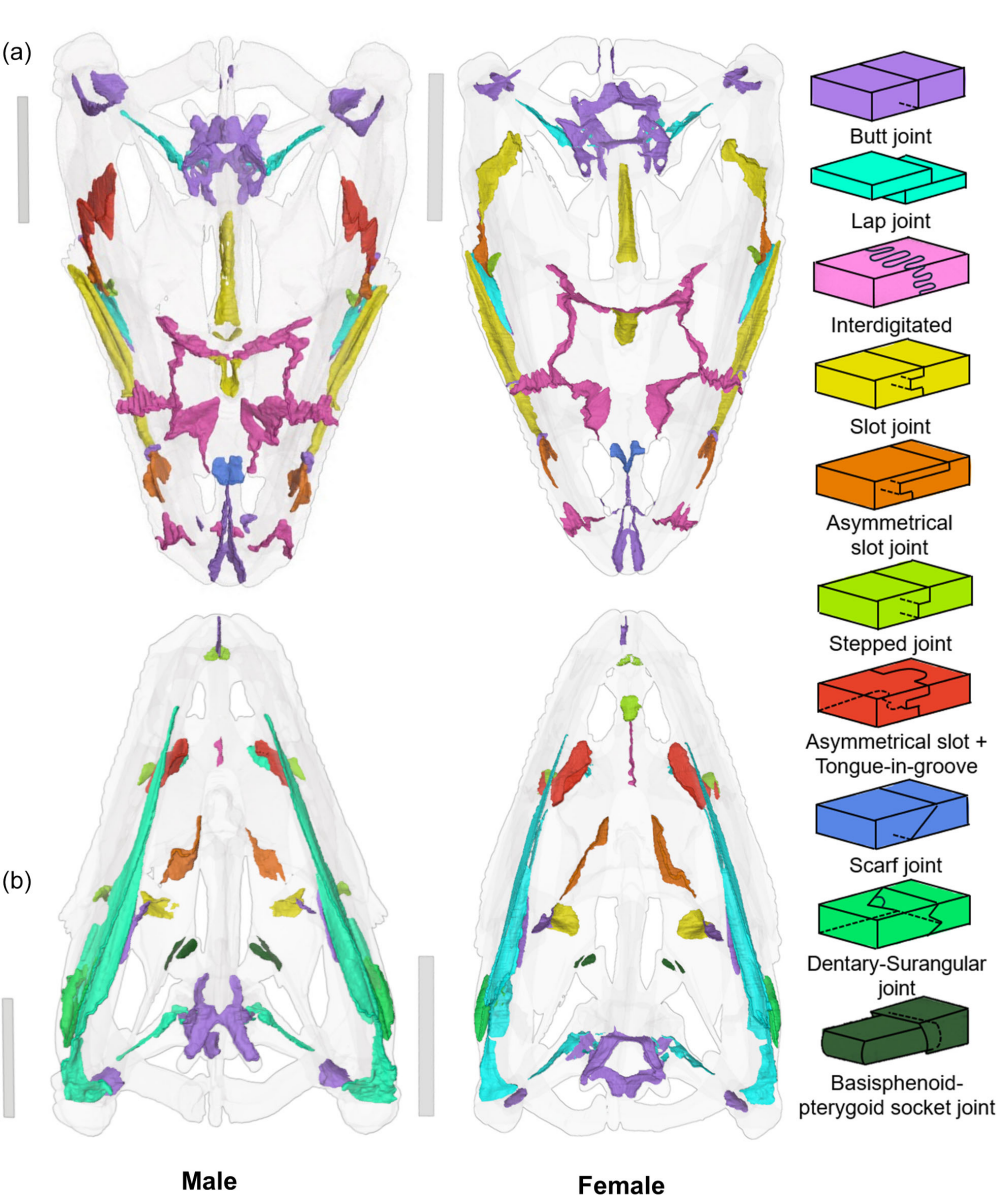
Map of the sutures in the skull of *Chamaeleo calyptratus*, visualized in Blender (Version 4.2). The female (right) has been scaled to the same mandibular length as the male (left). The dorsal view (a) shows the sutures of the facial skeleton, casque, and braincase. The ventral view (b) shows the sutures of the palate, lower jaw, and braincase. The key shows how each suture type is color‐coded and is ordered from most to least frequent suture type. The gray scale bars next to each figure are 10 mm in length.

The facial skeleton shows the largest difference in relative volume between the male and female (6.18%), with it being the largest contributor to total skull volume in the female, and the second largest in the male (Table 1). As with other chameleons, the premaxilla is unpaired (Anderson & Higham, 2014). Unlike in *C. chamaeleon* ([vulgaris]; Rieppel, 1981), this bone does not appear to have any vestigial teeth, but previous histological studies (e.g., Buchtová et al., 2013) and our own observations of younger specimens confirm the existence of two premaxillary teeth. As with most of the maxillary teeth that first calcified during embryonic development these are completely worn off in many adult specimens. The premaxilla fully separates the maxillae at butt joints and partially separates the nasals via butt joints at its posterior end, extending further posteriorly in the male compared to the female (Figures 1a and 3a). The nasal process is delicate and mediolaterally compressed. Similar to *Brookesia* but unlike *Bradypodion* (Anderson & Higham, 2014), the premaxilla has a small process on its ventral side known as the vomerine process, which extends posteriorly to underlap the vomer in a stepped joint (Figures 1b and 4b). This process is more pronounced in the male specimen. When the mouth is shut, the premaxilla meets the anterior end of the dentary at the midline.

According to previous literature (see Anderson & Higham, 2014, for a summary), the nasals are fused in *Chamaeleo*, but we find a clear anteroposterior suture between the left and right bones (butt joint; Figure 3a). The nasals ventrally lap the frontal, which separates them posteriorly (Figure 4a). The anterior ends of the nasals expand laterally to divide the prefrontal fenestra into dorsal and ventral portions (Figure 1a). As in other species of *Chamaeleo* (Anderson & Higham, 2014), the nasals do not form the dorsal edge of the external naris, which is formed by the anterolateral edge of the prefrontal.

The prefrontals are oriented anteroventrally and have a textured dorsal surface. The prefrontal contacts the maxilla both anterior and posterior to the external naris, forming its dorsal edge (Figure 1a). The anterior contact (i.e., with the facial process of the maxilla) is interdigitated (Figure 4a), and the prefrontal expands medially along the dorsal edge of the maxilla (Figure 1a). The posterior contact (i.e., with the orbital process of the maxilla) is an asymmetrical slot joint, where the medial edge of the prefrontal forms the longer side (Figures 1a and 3a). As in other chameleons (Anderson & Higham, 2014), the prefrontals are separated by the nasals anteriorly at butt joints and by the frontal posteriorly (interdigitated). The posterodorsal edge of the prefrontal contacts the postorbital (strongly interdigitated), while the posteroventral margin medially laps the palatine (Figures 1b and 3a). The smaller ventral division of the prefrontal fenestra is bound by the prefrontal laterally and partly posteriorly, while the larger dorsal division is bounded by the prefrontal at its lateral, posterior, and part of the anterior edges (Figure 1a). The prefrontal also forms the anterodorsal margin of the orbit.

The maxillae are the main tooth‐bearing bones of the upper jaw, and up to 23 teeth per side have been described (Buchtová et al., 2013). However, only 13 teeth per side can be counted in the male and 11 in the female. This likely indicates that all nine of the early‐initiating embryonic teeth and some of the five juvenile‐stage teeth have been worn off from usage (Buchtová et al., 2013). Unlike *C. chameleon* (Villa & Delfino, 2019), the maxillae approach but do not contact each other behind the premaxilla (premaxillary process) at the anteroventral margins of the vomer (Figure 1b), which they underlap in a stepped joint (Figure 4b). Posteriorly, the maxilla contacts the lacrimal, jugal, palatine, and ectopterygoid, details of which are provided in their descriptions below. In dorsal view (Figures 1a and 4a), the dorsomedial edge of its facial (frontal) process contacts the nasal at large scale interdigitations in the male and a butt joint in the female. The facial process forms the ventral and medial edges of the ventral division of the prefrontal fenestra. The maxilla also forms the anterior, ventral, and posterior edges of the nostril. While the orbital process extends anteriorly to contact the prefrontal and lacrimal, it is excluded from the orbit by the latter, as found in other species of *Chamaeleo* (Anderson & Higham, 2014).

The lacrimal is present in *C. calyptratus*, as in *Trioceros* ([*Chamaeleo melleri*]; Rieppel, 1981) and *Furcifer* (Rieppel & Crumly, 1997; Anderson & Higham, 2014). It joins the jugal at its posterior edge at a small slot joint in the male and a butt joint in the female (Figure 1a), as well as the lateral margin of the palatine at a stepped joint via a small medial expansion (Figures 2a and 3a). The entire ventral edge of the lacrimal contacts the maxilla at a slot joint, in which the lacrimal forms the pocket (Figure 3a). Together with the prefrontal (butt joint) and palatine, the lacrimal forms the medial margin of the nasolacrimal canal, which is situated just inside the anteroventral edge of the orbit (Klembara et al., 2017; Figure S2). The nasolacrimal canal is larger in diameter in the female (Table 2). The lacrimal also contacts the prefrontal at the anterior edge of the orbit at a butt joint.

The jugal is an “L”‐shaped bone with a scalloped posterior edge, where small projections extend posterolaterally—the male specimen has five distinct projections while the female specimen has three small projections (Figure 1a). The postorbital process of the jugal contacts the postorbital on its medial and anterior edges at an asymmetrical slot joint in which the medial side is expanded (Figure 3a). Ventral to this, the medial edge of the jugal contacts the ectopterygoid at a lap joint (Figure 1b; Figure 3a). The entire ventral margin of the jugal (maxillary process) contacts the posterodorsal edge of the maxilla at a slot joint where the maxilla forms the pocket (Figure 3a). The jugal forms the ventral and posteroventral edges of the orbit, as well as the anterior margin of the posttemporal fenestra, where its dorsal tip meets the squamosal at a butt joint (Figure 1a; Figure 3a). This contact, which is only found in *Chamaeleo*, *Furcifer*, and *Trioceros* species (Anderson & Higham, 2014; Rieppel, 1981), is larger in the male specimen.

**FIGURE 5:**
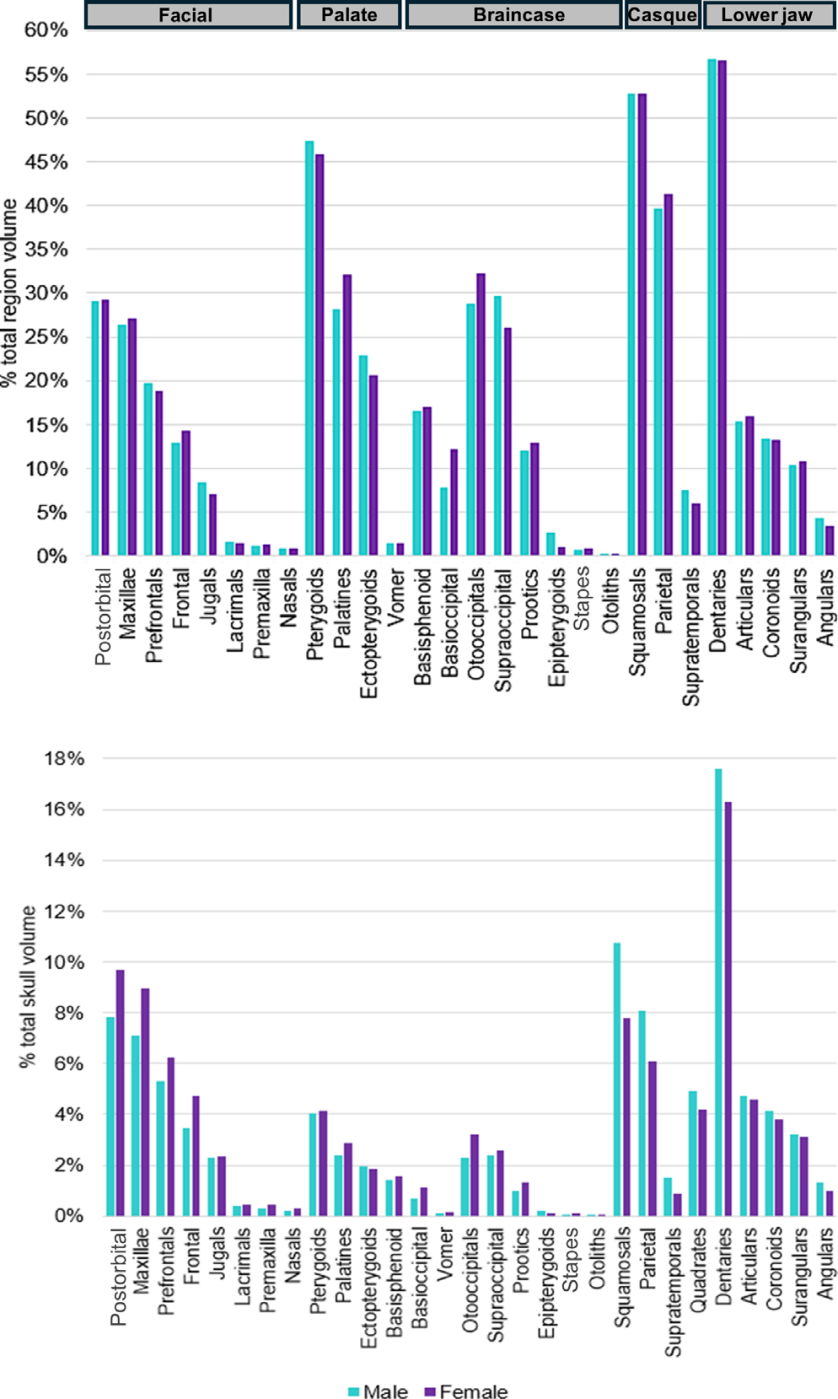
Summary of volumetric differences between the male and female skull (left and right sides combined) relative to total regional volume (top) and total skull volume (bottom). The quadrates are excluded from the regional plot, where the five different skull regions are indicated by the horizontal bars.

Combined, the postorbitals are the largest contributors to the facial skeleton in terms of volume (Figure 5). Dorsally, the surface of the postorbital is textured. Its medial edge contacts the frontal and the most anterolateral aspect of the parietal (both interdigitated, but weaker compared to its contact with the prefrontal; Figures 1a and 4a). On the ventral side, the posteromedial edge of the postorbital contacts the ectopterygoid at a stepped joint (Figure 3a). Dorsal and medial to where the jugal contacts the squamosal, the postorbital extends posteriorly and contacts the anterodorsal edge of the squamosal (Figure 1a). This posterior process expands dorsomedially over the squamosal, reducing the latter bone's contribution to the margin of the post‐temporal fenestra. The primary suture type for this contact is a slot joint in which the postorbital forms the pocket (Figure 3a). In the male, the pocket is deeper toward the dorsal and ventral ends, and the edges are more complex, where the lateral side is shaped as a large “tongue in groove” joint (Figure 3a). The postorbital forms the posterodorsal margin of the orbit and the anterolateral margin of the upper temporal fenestra.

As with other species of *Chamaeleo* (Anderson & Higham, 2014; Villa & Delfino, 2019), the frontal is a single fused bone with a textured dorsal surface that is oriented anteroventrally down the midline of the skull. It is excluded from the orbit (Figure 1a). The posterior edge of the frontal contacts the anterior margin of the parietal (interdigitated, weaker in the female; Figure 4a). At the medial point of this contact, the frontal has a dorsal expansion which straddles the parietal. The pineal foramen opens anteriorly in the middle of this expansion and traverses the whole frontal almost perpendicularly (Figure S3). The frontal forms the posteromedial margin of the prefrontal fenestra, similar to what has been found in other *Chamaeleo* species (Anderson & Higham, 2014).

#### 3.1.2 Palate

The vomer, palatine and pterygoid are rigidly connected to each other to form a firm palate that supports the muscles required for tongue protraction (Iordansky, 2016). Considering the number of bones involved, there is a relatively small difference in the relative volume of the palate between the sexes (1.07%; Table 1). There are also no palatal teeth (Figure 2b), unlike in some other reptiles (Villa & Delfino, 2019).

The vomer is unpaired, which is common across chameleons (Rieppel, 1981). At the posterior end, it contacts the palatines at a stepped joint (Figures 2b and 4b), where it separates slightly into a “V” shape in the male, similar to what is found in *C. chamaeleon* (Villa & Delfino, 2019).

The palatines contact each other anteromedially (interdigitated, with a larger contact in the female; Figure 4b) along their vomerine processes, which contact the vomer. The maxillary process of the palatine extends anteroventrally and lateroventrally below the contact with the prefrontal to articulate with the palatal process of the maxilla. This is an asymmetrical slot joint combined with a tongue‐in‐groove joint, in which the lateral edge of the palatine forms the pocket (Figures 2b and 4b). The posterior half of the palatine (pterygoid process) contacts the palatine process of the pterygoid on its medial (butt joint) and posterior edges (asymmetrical slot joint where the longer side of the pocket is formed by the dorsal edge of the palatine; Figure 4b). The palatine forms a small part of the ventral edge of the nasolacrimal canal and the entire medial edge of the infraorbital fenestra (Figure S2).

The lateral edge of the ectopterygoid is triradiate, with its postorbital process expanding both anteriorly and posteriorly along the jugal as well as posterodorsally to contact the postorbital. The ventral edge of this process also contacts the posteromedial part of the maxilla at a butt joint (Figure 1b). Medially, the pterygoid process of the ectopterygoid clasps the pterygoid through a medial deep slot joint in which the ectopterygoid receives a small projection from the pterygoid (ectopterygoid process of the pterygoid), as well as a ventral expansion (butt joint; Figures 1b and 4b). When the mouth is closed, the lateroventral edge of the ectopterygoid aligns with the coronoid, immediately anterior to the dorsal coronoid process.

The pterygoid is a large thin bone structure that fans out at the posterior end of the palate. It is also the largest contributor to the palate in terms of volume (Figure 5). As described above, it expands anteriorly to connect with the palatine and inserts into the ectopterygoid via a lateral expansion. The pterygoid has a posterolateral expansion, the quadrate wing of the pterygoid, and a posteroventral expansion, the pterygoid flange, that abuts the medial aspects of the coronoid and articular when the mouth is closed. These expansions appear broader and better ossified in the male (Figure 1b). While the quadrate wing of the pterygoid is only connected to the quadrate via a ligamentous sheet (Anderson & Higham, 2014), the ventral expansion of the pterygoid process of the ectopterygoid connects anterolaterally with the pterygoid flange.

#### 3.1.3 Braincase

The braincase has rarely been described in previous studies of chameleon cranial anatomy (see Villa & Delfino, 2019). Compared to the other regions of the skull, the volume of the braincase is relatively small (Table 1).

In posteroventral view, the basisphenoid is “X” shaped and forms the anterior floor of the braincase. Dorsally, it houses a concavity (*dorsum sellae*) that continues onto the dorsal aspect of the basioccipital. The lateral walls of the concavity (alar processes) contact the lateral lamina of the prootic at a butt joint (slightly interdigitated in the male, Figure 4). The parasphenoid process extends from the anterior end of the body of the basisphenoid along the midline just ventral to the dorsum sellae (Figure 2b). The paired basipterygoid processes (the anterior legs of the “X”) extend anteroventrally to contact sockets on the posterodorsal aspect of the pterygoids (Figure 3b; Figure 4b), forming the basal articulations. The posterolateral expansions (the posterior legs of the “X”) contact the basioccipital at a butt joint (Figure 3b).

The dorsal aspect of the basioccipital continues the concavity on the basisphenoid (*dorsum sellae*). Along the lateral walls of the *sella*, the basioccipital contacts the prootic anteriorly and the otooccipital posteriorly at butt joints (Figures 1b and 4b). The centre of the basioccipital expands posteriorly to form the ventral component of the occipital condyle, and it has two blunt posterolateral expansions (basal tubercles) (Figure 1c; Anderson & Higham, 2014).

**FIGURE 6:**
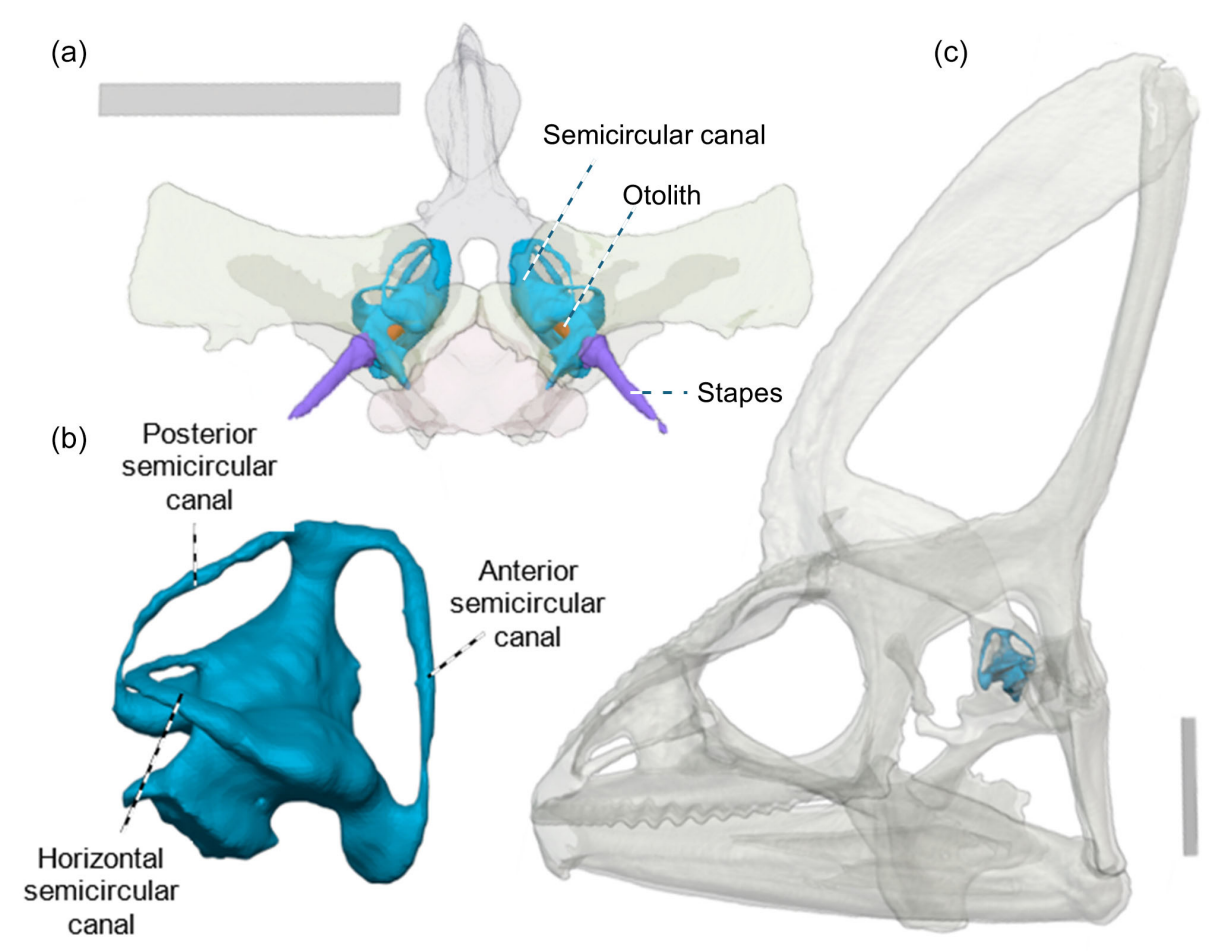
The semicircular canals, represented by an endocast of the inner ear of the male specimen: (a) ventral view of the semicircular canals (cyan) in relationship to the otooccipitals (transparent), prootics (transparent), supraoccipital (transparent), otoliths (orange) and stapes (purple); (b) lateral view of the right inner ear endocast showcasing the semicircular canals; (c) left lateral view of skull (transparent) to show the location of the semicircular canals. The scale bars in a and c represent 10 mm in length.

The otooccipitals are the largest contributors to the braincase in terms of volume (Figure 5). They span nearly the entire width of the posterior end of the skull to form broad rectangular wings, the paroccipital processes (Figure 2a). The lateral edge of the wing contacts the posteroventral side of the supratemporal at a butt joint (Figure 3b). Here, the ventral edge of the otooccipital approaches but does not contact the quadrate. The ventral side of the otooccipital bones meets medially at a butt joint, where each contributes a dorsolateral component to the occipital condyle and contacts the basioccipital. The otooccipitals expand anterodorsally from the condyle to contact the supraoccipital at a butt joint, forming the ventral half of the foramen magnum (Figures 2a and 3b). The posterior semicircular canal passes through this otooccipital and supraoccipital contact internally (Figure 6). Anteriorly, the otooccipital contacts the prootic at a lap joint (Figure 3b). Internally, both the horizontal and anterior semicircular canals pass through this otooccipital and prootic contact (Figure 6). The opisthotic portion of the otoccipital forms the posterior walls of the *cavum capsularis* and the cochlear cavity, and the posterior half of the oval fenestra (Figure 6a).

The otoliths—small, egg‐shaped inner ear bones—are situated between the otooccipital and the prootic (Figure 6a). The stapes (columella in Wever, 1968, 1969), a structure of the inner ear, were well ossified for both our specimens. The large stapedial footplate of this bone almost entirely covers the oval fenestra (Figure 6a), similar to other *Chamaeleo* species (Wever, 1968, 1969). The stapes taper ventrolaterally (Figure 2a).

In anterior view, the main body of the prootic has a curved medial edge, forming the sides of the braincase, and a well‐developed *crista alaris* extending from its lateral edge. Ventrally it has a flattened expansion that includes the lateral lamina (anterior inferior process of Villa & Delfino, 2019) and forms the anterior half of the oval fenestra. Posteriorly, it forms the anterior walls of the *cavum capsularis* and the cochlear cavity (Figure 6a). It has a posterodorsolateral expansion (posterior process) that contributes to the paroccipital process (Figure 2b). The dorsomedial edge of the prootic contacts the supraoccipital at a butt joint (Figures 1b and 3b) and the anterior semicircular canal lies within both bones (Figure 6).

The supraoccipital expands anteriorly and dorsally such that most of its dorsal edge underlaps the ventral surface of the parietal, which separates it from the frontal (Figure 1b). This anterodorsal expansion (*processus ascendens*) is lateromedially flattened and taller in the male compared to the female. Anteriorly, on the ventrolateral sides of the supraoccipital, two small but well‐developed cylindrical processes extend anteriorly (Figure 2a). Posteriorly, the ventral edge of the supraoccipital forms the dorsal half of the foramen magnum. The anterior and posterior semicircular canals lie within the supraoccipital (Figure 6).

On the ventral side of the brain, just posterior to where the optic nerves merge, are the epipterygoids, which are more robust in the male (Figure 1b). This is contra to previous descriptions claiming that epipterygoids are absent in chameleons (Evans, 2008; Iordansky, 2016; Villa & Delfino, 2019) due to the loss of amphikinetism. While these long, thin structures do not contact any other bones, the ventral ends of the epipterygoids are positioned dorsolaterally to the basipterygoid processes (Figure 2b).

#### 3.1.4 Casque

The casque is one of the skull regions which differs most between the sexes, The relative volume of the male casque is 5.65% larger than the female casque (Table 1), and it is 22.8% taller relative to total skull height in the male (Table 2). The parietal is the largest unpaired bone in the skull and shows significant size differences among the sexes relative to total skull volume (Figure 5). It forms the anterior edge of the tall tetrahedral casque that characterizes the skull shape of *C. calyptratus*. The parietal has a significant posterodorsal expansion (parietal crest; Figure 1a), especially in the male, which gradually increases in dorsoventral height posteriorly (Figure 1b) and is mediolaterally flattened throughout most of its length (particularly in the male). The posterodorsal tip loosely contacts the two squamosals at butt joints (Figures 2a and 3a). The posteroventral expansion of the parietal (*processus parietalis inferior* in Čerňanský et al., 2014; median crest in Villa & Delfino, 2019), has the structure of a tall arch, forming a deep slot joint with the anterior half of the supraoccipital's *processus ascendens* (Figures 1b and 3a). In addition to this slot joint, the parietal presents a depression on its posteroventral side, just at the base of the medial crest, where the tip of the *processus ascendens* would fit at a butt joint. The height of the *processus parietalis inferior* is consistent in the male, while the anterior end expands dorsally in the female, forming a much thicker base of the parietal. The ventrolateral edge of the parietal forms the anterior margin of the upper temporal fenestra, while the parietal crest forms its medial margin (Figure 1a).

Combined, the squamosals are the largest contributors to the casque, and they show the highest difference in relative volume between the sexes (2.98% larger in the male; Figure 5). The squamosal has a large dorsal expansion (ascending process; parietal process in Villa & Delfino, 2019) that angles posteriorly to meet the parietal, forming the posterior edges of the tetrahedral casque (Figure 1b) and the posterolateral boundary of the upper temporal fenestra (Figure 1a). At the base of the ascending process, the lateral side of the squamosal has a textured surface – this extends further dorsally up the ascending process in the female (Figure 1a). Ventrally, the squamosal has anterior and posterior processes that form the dorsal and posterior margins of the upper temporal arch and posttemporal fenestra (Anderson & Higham, 2014). The anterior process contacts the postorbital and the jugal, as described above, and the posterior process (quadrate process in Villa & Delfino, 2019) articulates with the lateral head of the quadrate's cephalic condyle.

The supratemporal is tetrahedral, in which almost its entire anterior surface expands dorsally up the posteromedial edge of the posterior process of the squamosal (butt joint; Figure 3) until it reaches the same height as the top of the facial skeleton (Figures 1b and 2a). Following its contact with the squamosal, the supratemporal is angled more medially in the female. Ventral to its contact with the otooccipital, the supratemporal forms the articular surface for the head of the quadrate.

#### 3.1.5 Quadrate

The quadrate is a vertically oriented, rod‐shaped bone with a mediolaterally wider dorsal end (Figure 1a). The structure of this bone and its contacts with surrounding soft tissue have been strongly related to the movement of the jaw apparatus during prey capture (Iordansky, 2016). The cephalic condyle (dorsal end) is subdivided into three structures: the central and flat articular surface for the supratemporal, a small lateral head that articulates with the squamosal, and a large medial head for increased muscle attachment (AMEP3a; see Section 3.2.2). The pillar is anteroposteriorly compressed. The anteroventral edge of the quadrate contacts the surangular at a butt joint, while its ventral end, which forms the mandibular condyle, has a synovial joint with the articular (Figure 3b). The ventral end of the quadrate is approximately two to three times wider mediolaterally than anteroposteriorly. It is “saddle shaped”—convex in anteroposterior view and concave in mediolateral view—and has two condyles, separated from each other by a midline indentation. The lateral condyle is smaller and has a tighter corresponding contact surface with the articular than the medial condyle. Most of the posterior margin of the posttemporal fenestra is defined by the anterior edge of the quadrate. The quadrate is larger in the male (Table 1), especially the pronounced medial head of the cephalic condyle (Figures 1a and 2a).

#### 3.1.6 Lower jaw

The lower jaw is the largest contributor to skull volume in the male, and second most in the female (Table 1), largely due to the combined size of the dentaries (Figure 5).

**FIGURE 7:**
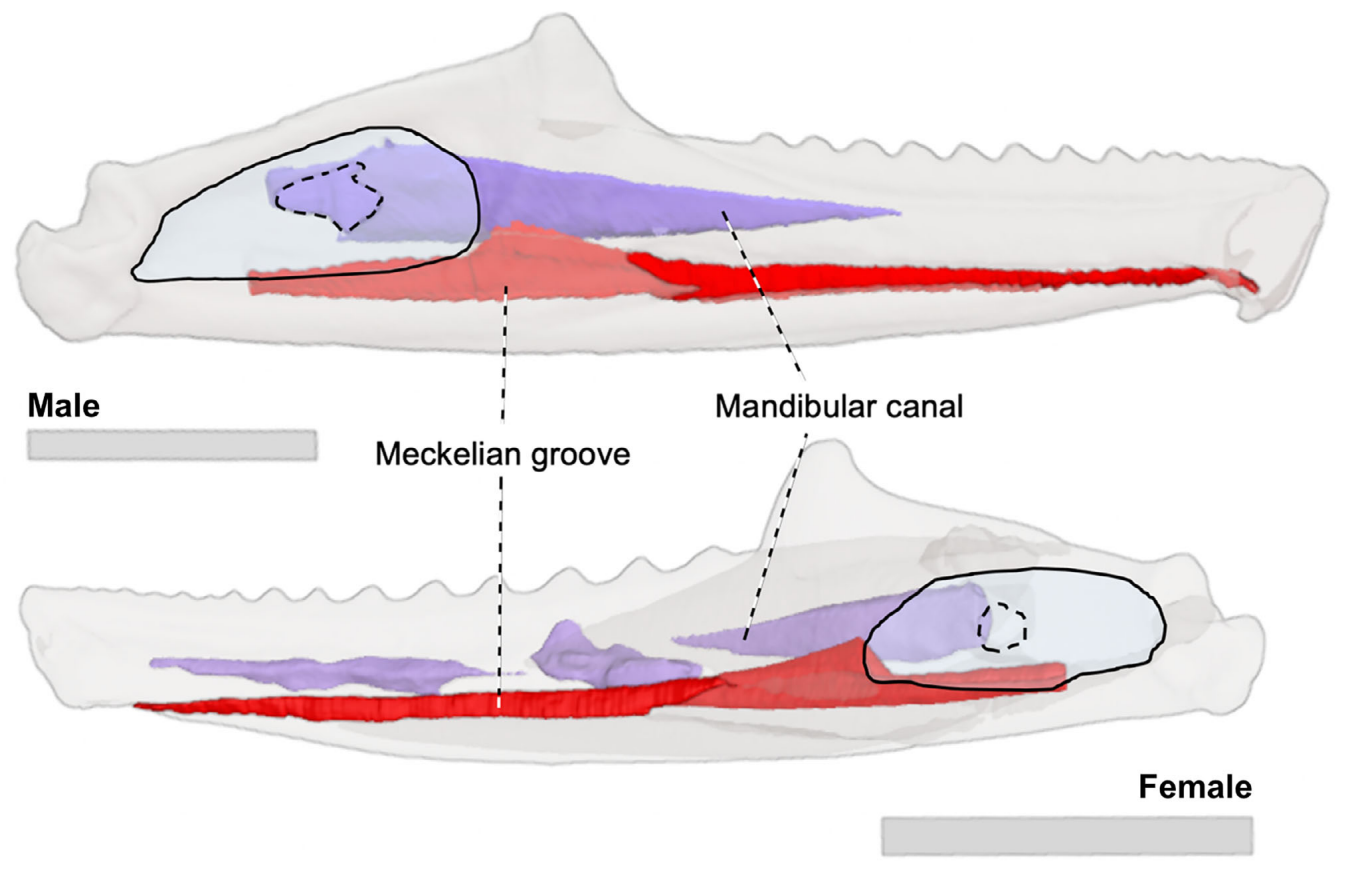
The internal architecture of the lower jaw in male (top) and female (bottom) *Chamaeleo calyptratus*. The mandibular adductor fossa is outlined by the solid line, while the mandibular foramen—the entry to the mandibular canal—is indicated by the dashed line. The main cavity of the mandibular canal has been highlighted in this figure, but there are many smaller branches not represented here for simplicity. The gray scale bars next to each figure are representing 10 mm in length.

The dentaries are the only tooth‐bearing bones of the lower jaw, with up to 21 teeth being present in adults (Buchtová et al., 2013). However, as with maxillary teeth, only 12 and 13 per side can be counted in the male and female, respectively. As with the maxillae, only the five teeth that form early during postembryonic growth and the seven teeth that grow later in life remain, with the nine early initiating embryonic teeth mostly worn off from use. The dentaries are joined anteromedially via a butt joint at the mandibular symphysis, showing a tighter contact in the male (Figure 3a). The rounded anterior borders and the broad, flattened posterior borders of the symphyseal articulation surface suggest a higher degree of anterior rotation (mediolateral spreading) than posterior rotation (mediolateral narrowing) along the dorsoventral axis of the mandibular symphysis. The dentary contacts each bone of the lower jaw, described in detail below. There is a mandibular canal within the dentary (alveolar canal in Villa & Delfino, 2019) that runs anteriorly from an opening between surangular and dentary on the medial side (Figure 2b). This canal contains blood vessels and the mandibular (V3) branch of the trigeminal nerve (Figure 7). The posteroventromedial surface of the dentary forms part of the mandibular adductor fossa, where the deeper bundle of the pterygoideus muscle inserts. The Meckelian groove runs ventral to this surface along the ventromedial edge of the dentary (Figure 7).

The coronoid extends anteriorly ventral to the three most posterior teeth, lapping the medial side of the dentary (anteromedial process; Figures 1b and 3a). Posterior to the end of the tooth row, is the dorsal coronoid process (Anderson & Higham, 2014), which is taller relative to the height of the tooth row in the male (Figure 7). The coronoid also extends ventrally (posteromedial process) to contact the anterolateral side of the articular at a butt joint (Figure 3a), but not the dentary, instead forming a medial wall to the Meckelian groove anteriorly to the mandibular adductor fossa (Figure 7). The dorsal portion of the coronoid extends further posteriorly (posterior process), beyond the end of the dentary, to contact the anterodorsal side of the surangular at a lap joint (Figures 1b and 3a).

When viewed medially (Figure 1b), the surangular contacts ventral and dorsal aspects of the posterior end of the dentary, forming a horizontal “V” shape. There is a distinct notch at the apex of this “V” where the surangular forms the posterior edges of the mandibular foramen (with its anterior edges formed by the dentary; Figures 1b and 7). When viewed laterally (Figure 1a), the anterior edge of the surangular contacts the dentary in an “M” shape (Figure 3a). There is a considerably smaller opening, also classed as the mandibular foramen (Jones et al., 2009), at the apex of this “M” (Figure S4). The anteroventral side of the surangular contacts the posterodorsal edge of the angular in a lap joint (Figure 2b), while its posteroventral side laps the articular lateral and anterior to the jaw joint (Figures 1b and 3a). The posteromedial side of the surangular forms the posterior wall of the mandibular adductor fossa.

Contrary to Villa and Delfino (2019) but in agreement with Anderson and Higham (2014), we were able to separate the angular and articular and thus do not support the presence of a compound bone in chameleon mandibles. The angular is a long and thin bone which contacts the ventromedial aspect of the dentary and the articular in lap joints (Figure 4a). This contact extends further anteriorly in the male than in the female (Figure 2b). Posteriorly, the angular curves laterally to also contact the ventrolateral aspect of the articular in a lap joint (Figures 2b and 4a).

The articular laps the medial side of the posterior half of the dentary (Figure 3a). The anterior two‐thirds of the articular has a dorsal indentation that forms the ventral and lateral edges of the Meckelian groove (Figures 1b and 7). This anterior portion is described as the prearticular process in Villa and Delfino (2019) and would represent the remains of a prearticular bone fused with the articular. Immediately medial to the groove, a depression on the articular forms the floor of the mandibular adductor fossa. As observed in previous studies, the retroarticular process of the jaw joint is reduced (Anderson & Higham, 2014; Villa & Delfino, 2019), implying a relatively smaller moment arm for the depressor mandibulae compared to other reptiles. Where it contacts the quadrate, the articular is mediolaterally convex and anteroposteriorly concave, and has two articular surfaces separated by a rounded midline ridge. The movement of the quadrate is likely restricted by the strong ridges on all sides of this joint surface, with the medial ridge being most pronounced.

### 3.2 Cranial muscles

**FIGURE 8:**
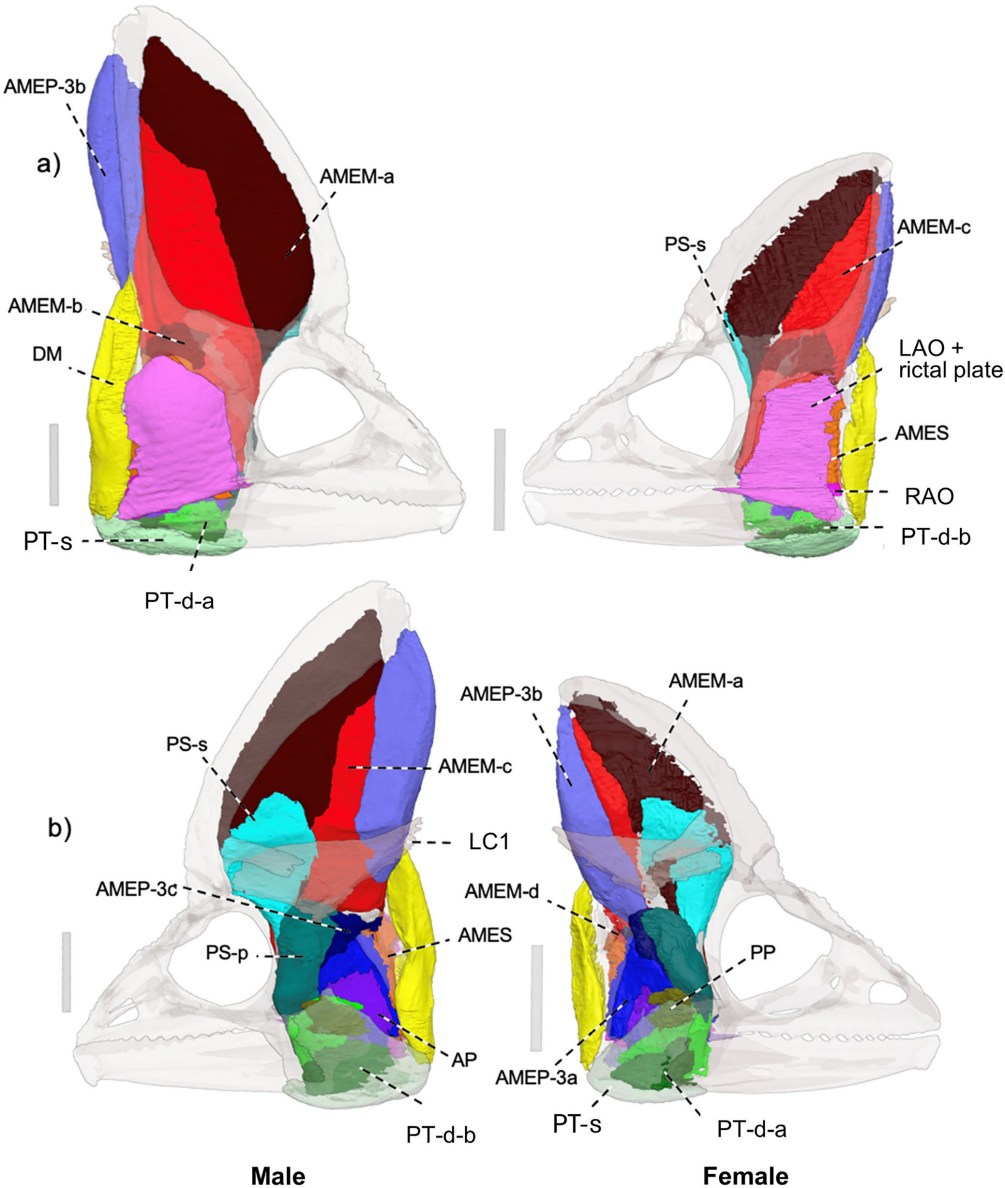
3D digital reconstruction of the cranial muscles of *Chamaeleo calyptratus*, visualized in Blender (Version 4.2) in (a) lateral and (b) medial views. The male (left) has the muscles displayed on the right side of the skull, while the female (right) shows the muscles on the left side of the skull. The female has been scaled to the same mandibular length as the male. The longissimus capitis 1 (LC1) and pterygoideus superficial (PT‐s) have been made partially transparent in the medial view to allow deeper muscles to be visualized. See Table 3 for the full names of each abbreviated label. See Supplementary Figures S5–S15 for visualizations of each muscle complex in isolation. The gray scale bars next to each figure represent 10 mm in length.

**FIGURE 9:**
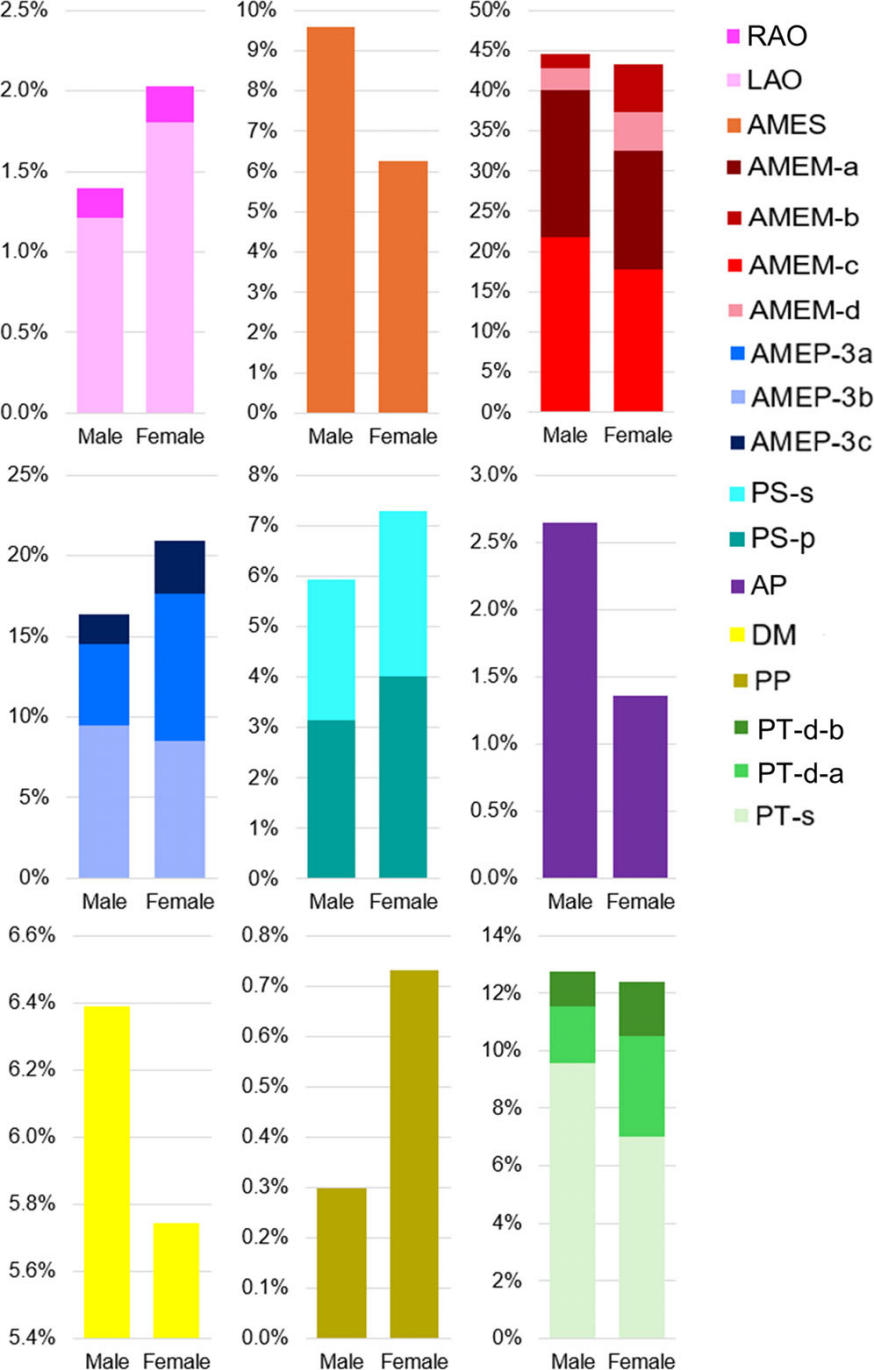
A summary of the differences in muscle volumes between male and female relative to total measured cranial muscle volume (i.e., excluding the neck muscle LC1). The graphs are grouped by muscle complex and color coded to match Figure 8. Vertical axes are different for each muscle complex to facilitate easier visualization of the differences between the sexes. See Table 3 for the full names of each abbreviated label.

The cranial musculature of *C. calyptratus* is dominated by a large series of jaw adductor muscles (Figure 8; Figure 9), which could be further divided into an externus and internus complex (Table 3; Daza et al., 2011). Many of the jaw adductor muscles can be identified based on their position relative to the bodenaponeurosis—a large tendon that travels vertically through the skull, originating from the muscles occupying the casque to an insertion point on the dorsal side of the coronoid process (Figure S5; Rieppel, 1981). Continuing in order of contribution to total cranial muscle volume, the remaining muscles consist of the depressor mandibulae complex, the most superficial lateral muscles, and the constrictor dorsalis musculature (Table 3; Figure 9).

**TABLE 3:** Summary of *Chamaeleo calyptratus* cranial myology, color‐coded to match Figure 8 and Supplementary Figures S4–S15.

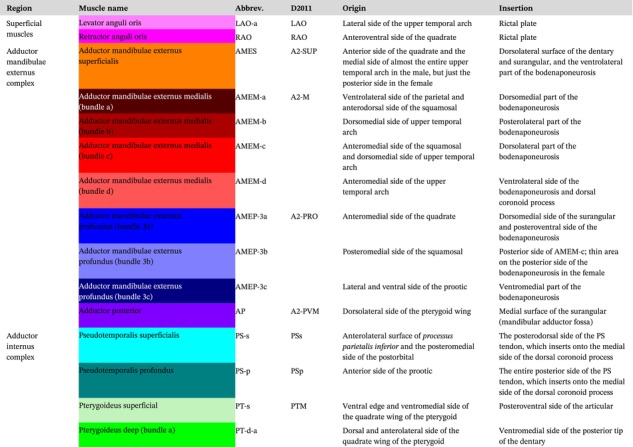
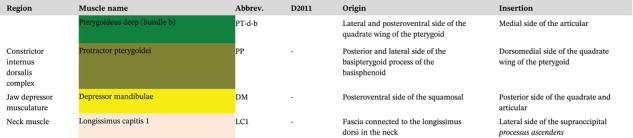

*Note*: Abbrev.—the muscle name abbreviations used throughout the manuscript; D2011—the muscle name abbreviations used in the muscle homology study by Daza et al. (2011).

**FIGURE 10:**
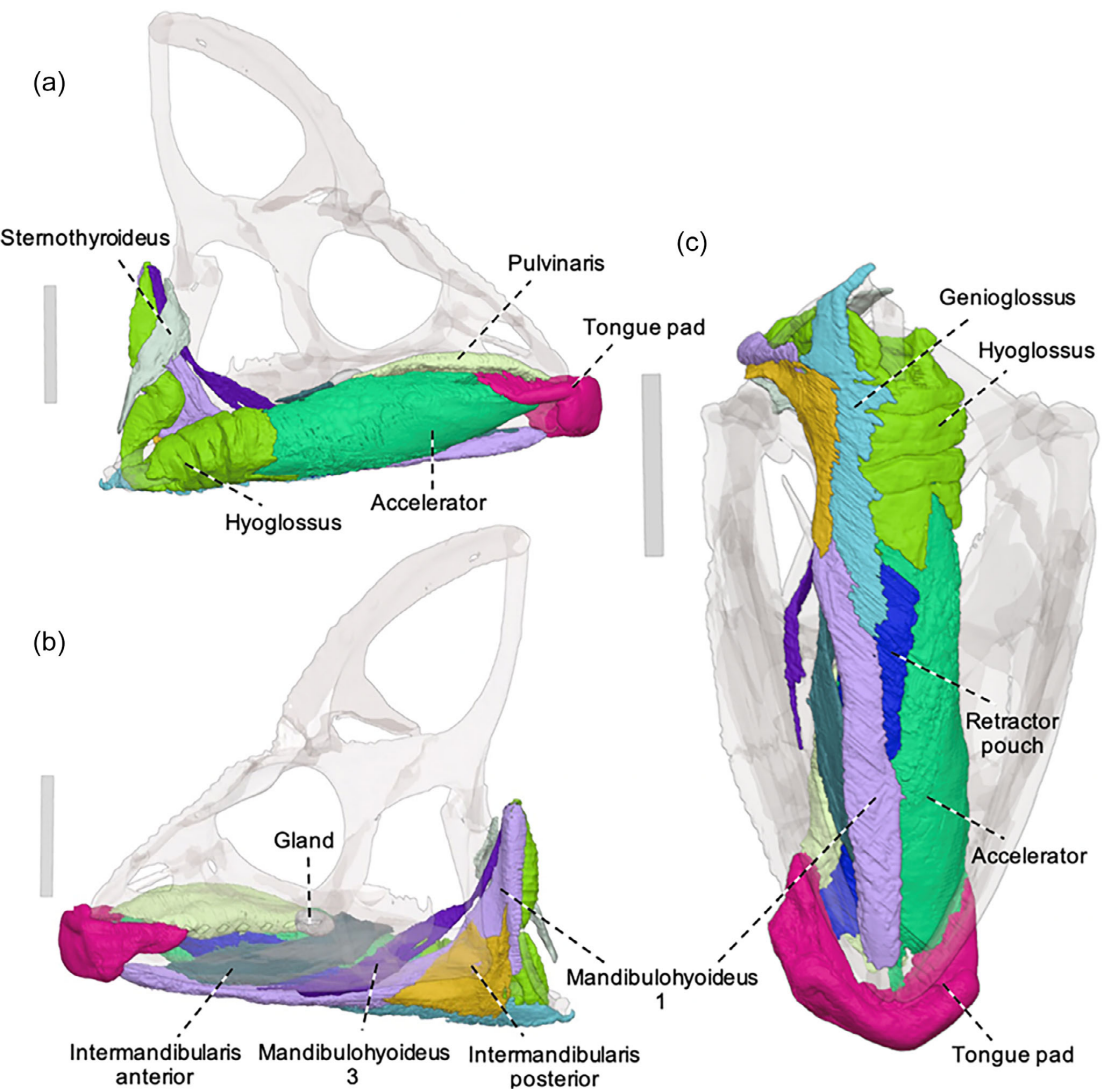
3D digital reconstruction of the left and central hyolingual muscles of a female *Chamaeleo calyptratus*, visualized in Blender (Version 4.2) in (a) lateral, (b) medial, and (c) ventral views. The gray scale bars next to each figure represent 10 mm in length.

Here, we provide the first description of each cranial muscle for *C. calyptratus*, following the same nomenclature and abbreviations as used in previous literature (Anderson & Higham, 2014; Rieppel, 1981; Haas, 1973). Their correspondence with the nomenclature used in Daza et al. (2011) is also provided in Table 3. We present details on the origins, insertions, and muscle fiber orientation in the male specimen, then note any anatomical differences present in the female. While the hyolingual muscles are not described here (see Herrel et al., 2001, 2009), the scan quality of the female specimen was high enough to include many of these muscles in the digital atlas (Figure 10).

#### 3.2.1 Superficial muscles

**FIGURE 11:**
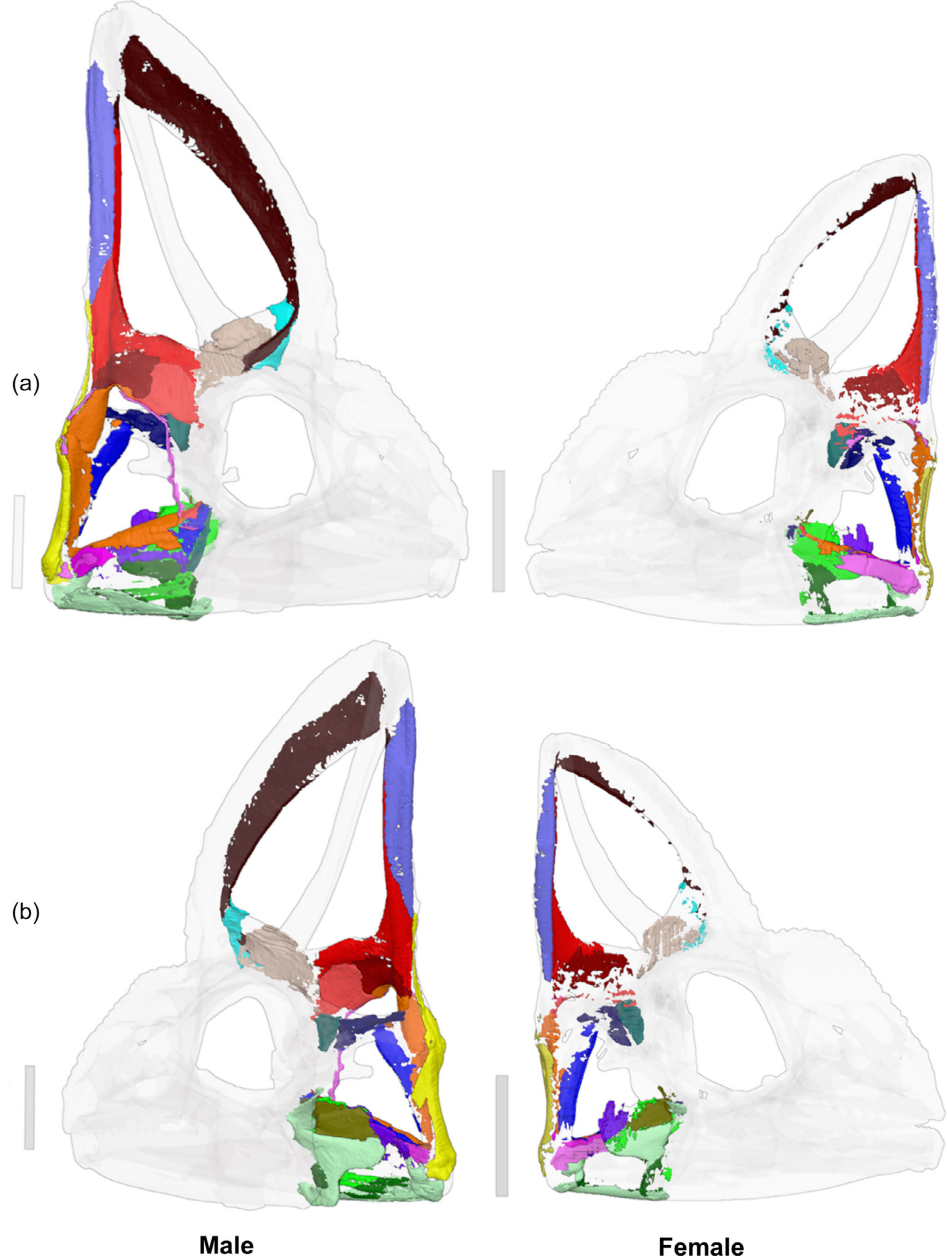
The muscle attachment sites on the skull in *Chamaeleo calyptratus*, visualized in Blender (Version 4.2) in (a) anterolateral and (b) posteromedial views. The male (left) has the muscle attachment sites displayed on the right side of the skull, while the female (right) shows the attachment sites on the left side of the skull. The female has been scaled to the same mandibular length as the male. The gray scale bars next to each figure represent 10 mm in length. See Figure 8 and Table 3 for muscle colors.

The levator anguli oris (LAO) and retractor anguli oris (RAO) are the most superficial jaw muscles (Figure 8a), being almost indistinguishable from the skin on PMA‐stained scans. Unlike *Trioceros* (Rieppel, 1981), there was no clear split of the LAO into anterior and posterior heads. The LAO originates on the upper temporal arch, while the RAO, which lies deep to the posteroventral region of the LAO, is considerably smaller and originates on the anteroventral surface of the quadrate (Figures 8a, 11, and S6). Both muscles insert onto the rictal plate—a fold of skin forming the corner of the mouth (Figure 11a; Figure S6)—and function to raise the corner of the mouth so that the cheek is not bitten when the jaw shuts.

#### 3.2.2 Adductor mandibulae externus complex

As in other chameleons, the jaw adductor musculature for *C. calyptratus* is well developed (Figure 8; Anderson & Higham, 2014). The externus complex is the largest component, and can be divided into the superficialis, medialis, and profundus muscles (Table 3).

**FIGURE 12:**
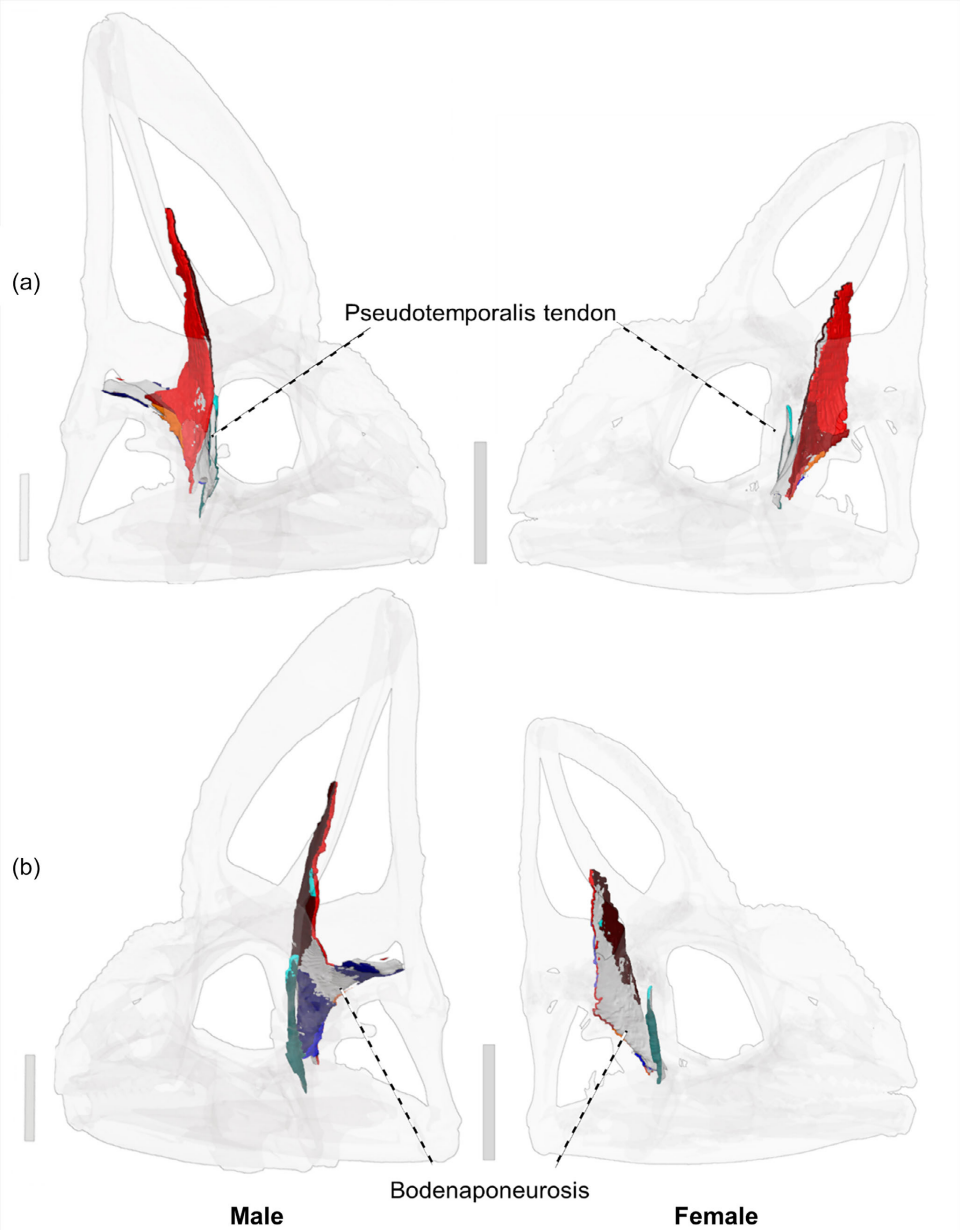
The tendinous muscle attachment sites in *Chamaeleo calyptratus*, visualized in Blender (Version 4.2) in (a) anterolateral and (b) posteromedial views. The male (left) has the attachment sites displayed on the right side of the skull, while the female (right) shows the attachment sites on the left side of the skull. The female has been scaled to the same mandibular length as the male. The gray scale bars next to each figure represent 10 mm in length.

Just deep to the LAO and rictal plate is the adductor mandibulae externus superficialis (AMES; Figures 8a and S7). AMES is larger in the male (Figure 9), originating along the entire ventral aspect of the medial surface of the upper temporal arch, but only from the posterior side of the ventral aspect in the female (Figures 11 and S7). The AMES has fibers oriented from posterior to anterior, inserting on the dorsolateral surface of the dentary and surangular in both sexes (Figure 11), as well as the posterior edge of the bodenaponeurosis (Figure 12a). The medial side of the AMES is closely associated with bundle 3c of the adductor mandibulae externus profundus (Figure 8b).

The adductor mandibulae externus medialis (AMEM) is the largest contributor to cranial muscle volume, more so in the male (Figure 9). We found that it could be clearly divided into separate bundles in both specimens (Figures 8 and S8). The most anterior part of the muscle, herein referred to as “AMEM‐a,” originates on the ventral half of almost the entire lateral aspect of the parietal, as well as the anterodorsal surface of the squamosal (Figure 11a). With fibers oriented from anterior to posterior, AMEM‐a inserts onto the most anterodorsal part of AMEM‐c and onto most of the lateral side of the bodenaponeurosis (Figure 12b), occupying over half of the upper temporal fenestra when viewed laterally. AMEM‐c, the largest bundle (Figure 9), takes up the remainder of that space. This bundle is defined by its origin on the ventral two‐thirds of the anteromedial surface of the ascending process of the squamosal (Figure 11). With fibers oriented from posterior to anterior, AMEM‐c inserts onto the opposite side of the bodenaponeurosis as AMEM‐a (i.e., medially; Figure 12a). Additionally, a large artery travels across the medial side of AMEM‐a and AMEM‐c in an arc, before turning ventrally along the posterior side of AMEM‐c (Figure S8). The ventral surface of AMEM‐c is closely associated with AMEM‐b, the smallest bundle in the AMEM complex (Figures 8 and 9). AMEM‐b originates on the dorsal aspect of the upper temporal arch on its medial side (Figures 11 and S8). It has fibers oriented from posterior to anterior, inserting onto the lateral side of the posteroventral part of the bodenaponeurosis (Figure 12a). AMEM‐b is also closely associated with the dorsal side of AMES and dorsomedial edge of AMEM‐d, the most ventral bundle. AMEM‐d is larger in the female (Figure 9). It originates on the anteromedial side of the upper temporal arch (Figure 11) and courses ventrally to insert on the dorsolateral side of the bodenaponeurosis and coronoid process (Figures 8a, 12a, and S8). AMEM‐d also has fibers that are often challenging to distinguish from the anterodorsal side of the AMES.

The adductor mandibulae externus profundus (AMEP), which runs deep to AMEM and AMES, is divided into three bundles (Figure 8b; Daza et al., 2011; Rieppel, 1981). AMEP‐3b is the largest (Figure 9) and most dorsal part, known to vary widely between taxa (Anderson & Higham, 2014). As with AMEM‐a and AMEM‐c, AMEP‐3b is enlarged in *C. calyptratus* due to the size of the casque. In general, it originates along almost the entire posteromedial surface of the squamosal's ascending process (Figure 12b and S9). However, unlike in any previously described species of *Chamaeleo* (Anderson & Higham, 2014; Rieppel, 1981), AMEP‐3b does not extend anteriorly to occupy the entire casque, but it instead inserts onto AMEM‐c. In the male, there is a distinct, vertically oriented tendon clearly marking this boundary on the medial side (Figures S6 and S9). Additionally, while AMEP‐3b has a small insertion point on the posterior side of the bodenaponeurosis in the female (Figure 12b), it does not insert here in the male specimen, contrasting with previous findings (Anderson & Higham, 2014). Moving ventrally is AMEP‐3c, the smallest bundle of AMEP (Figures 8 and S9), which is larger in the female (Figure 9). It originates on the lateral and ventral surfaces of the prootic (Figure 11) and inserts onto the ventromedial surface of the bodenaponeurosis (Figure 12b), not directly onto the coronoid process (as described in Anderson & Higham, 2014). At its most dorsal and ventral parts, bundle 3c is closely associated with the lateral side of pseudotemporalis profundus. Finally, AMEP‐3a is the most ventral bundle and is larger relative to total measured cranial muscle volume in the female (Figure 8; Figure 9; Figure S9). It originates on the anteromedial side of the quadrate and its fibers are oriented anteroventrally to insert on the dorsomedial surface of the surangular (Figure 11b) and the posteroventral surface of the bodenaponeurosis (Figure 12b). As observed in previous studies, this bundle was sometimes challenging to define (Rieppel, 1981), especially at the boundary it shares with the medial side of the AMES.

#### 3.2.3 Adductor posterior

The adductor posterior (AP) is found deep to AMEP‐3a (Figures 8 and S10). While previously reported to be large (Rieppel, 1981), we find that this muscle is relatively small in the female (Figure 9). It is also said to originate on the dorsolateral part of the pterygoid wing and the medial edge of the quadrate (Rieppel, 1981). Our findings correspond with the former observation, but we found that the AP does not originate on the quadrate (Figure 11a). AP has fibers oriented almost vertically from posterior to anterior, inserting on the medial surface of the surangular (Figures 11b and S10).

#### 3.2.4 Adductor internus complex

The internus complex of the jaw‐closing musculature consists of the pseudotemporalis and pterygoideus muscles. The pseudotemporalis (PS) has two heads—superficialis (PS‐s) and profundus (PS‐p)—that are relatively similar in size (Figures 8b, 9, and S11). PS‐s lies deep to AMEM‐a, originating on the anteromedial surface of the ventral parietal as well as the dorsomedial surface of the postorbital (Figure 11a). Unlike in other species (Rieppel, 1981), it does not expand anteriorly to cover the entire lateral aspect of the casque. The fibers of PS‐s are oriented posteroventrally to insert on the anterior side of a tendon it shares with PS‐p (Figure 12). This tendon, which is thicker in the male, lies deep to the bodenaponeurosis and inserts onto the medial aspect of the coronoid (Figure S11). PS‐p lies deep to AMEP‐3c, originating on the anterior edge of the prootic (Figure 11). Most of PS‐p inserts onto the posterior side of the PS tendon, while its most anteroventral part inserts onto the posterior surface of the bodenaponeurosis and the posteromedial side of the coronoid (Figure 12b).

The pterygoideus (PT) has two main heads—superficial (PT‐s) and deep (PT‐d)—and is generally larger relative to total measured cranial muscle volume in the male (Figure 8, 9, and S12). PT‐s originates on the ventral edge and ventromedial surface of the quadrate wing of the pterygoid, the ventromedial surface of the pterygoid flange, and on the basipterygoid process of the basisphenoid (Figure 11b). This latter attachment site is relatively uncommon for lizards and has been suggested to function as better support for PT during prey capture (Iordansky, 2016). Its insertion wraps around the entire posteroventral surface of the articular and angular (Figure 11a). In the male, this insertion covers both the medial and lateral surfaces of the articular and expands far enough anteriorly to have a small insertion on the most ventrolateral tip of the dentary (Figures 11a and S12). In the female, PT‐s does not cover as much of the lateral surface of the lower jaw. We also found that PT‐d can be separated into two distinct bundles (Figure S12), which have not previously been reported. PT‐d‐a is generally larger, especially in the female (Figure 9), originating on the dorsal edge and anterolateral aspect of the quadrate wing of the pterygoid and inserting on the lower edge and ventrolateral surface of the posterior tip of the dentary (Figures 11 and S12). PT‐d‐b originates on the ventrolateral surface of the quadrate wing of the pterygoid and the posterolateral surface of the pterygoid flange (Figure 11a). It inserts onto the medial surface of the articular, just ventral and anteroventral to the jaw joint (Figure 11b).

#### 3.2.5 Constrictor internus dorsalis complex

The only muscle of the constrictor internus dorsalis complex which could be found in *C. calyptratus* is protractor pterygoidei (PP). In our specimens, PP is similar to that of *C. melleri* in that it is not as strongly developed as in *Bradypodion* (Figure S13; Rieppel, 1981). Relative to total measured muscle volume, it is larger in the female (Figure 9). PP originates on the posterolateral surface of the basipterygoid process of the basisphenoid and inserts on the dorsomedial surface of the quadrate wing of the pterygoid (Figure 11b). As in other chameleons, we could not identify levator pterygoidei or levator bulbi muscles, which have been associated with a reduction in cranial kinesis in chameleons (Anderson & Higham, 2014).

#### 3.2.6 Jaw depressor musculature

The most posterior muscle in the skull is the depressor mandibulae (DM), which originates on the posteroventral part of the ascending process of the squamosal, as well as the posterior surface of the quadrate (Figure 11). Ventral to the cephalic condyle, the DM expands laterally (Figures 8a and S14). In the male, the DM wraps around the quadrate as far as its lateral ridge, contacting the posterior side of the ventral half of the AMES (Figures 8a and S14). As such, this muscle has a larger volume in the male (Figure 9). The DM then inserts onto the posterior surface of the retroarticular process in both specimens (Figure 11).

#### 3.2.7 Longissimus capitis 1

Despite a significant attachment site on the skull (Figures 8b; 11a), the longissimus capitis 1 (LC1) has not been included in previous descriptions of chameleon cranial anatomy (Rieppel, 1981) as it is a neck muscle originating from fascia attached to the longissimus dorsi. In other lizards, LC1 is known for inserting only on the parietal or squamosal, but chameleons are the exception (Al Hassawi, 2004). In other *Chamaeleo* species, this muscle has been said to only attach to the supraoccipital (Al Hassawi, 2004). However, we find that LC1 inserts along the lateral surface of both the supraoccipital *processus ascendens* and the parietal *processus parietalis inferior* in *C. calyptratus* (Figures 11a and S15).

### 3.3 Nervous system

**FIGURE 13:**
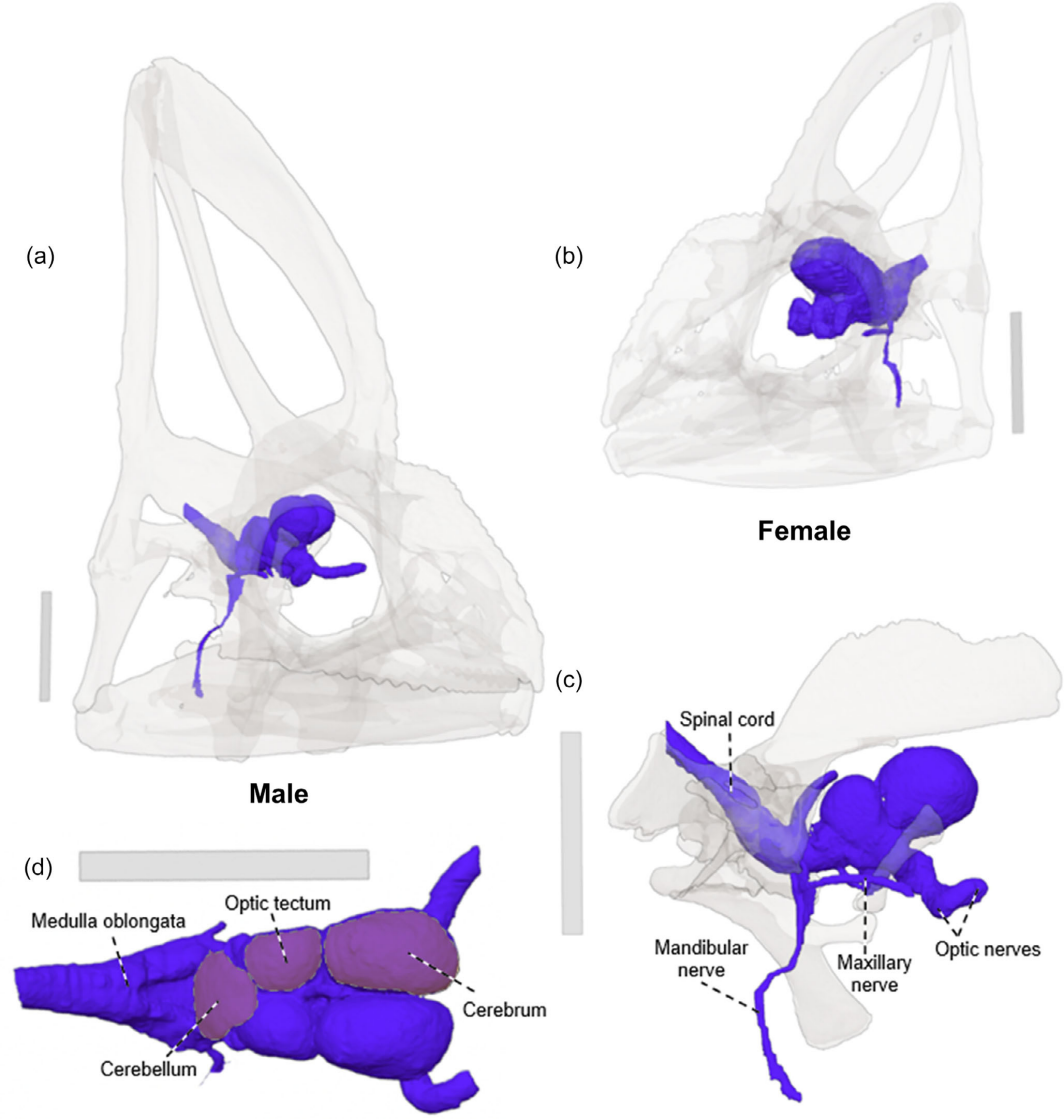
3D digital reconstruction of the brain and cranial nerves II and V of *Chamaeleo calyptratus*, visualized in Blender (Version 4.2): (a) right anterolateral view of the male skull (transparent); (b) left anterolateral view of the female skull (transparent); (c) anterolateral view of the right side of the male braincase, brain, and cranial nerves; and (d) dorsal view of the male brain. Note that the nerves travel further than depicted here. The gray scale bars next to each figure represent 10 mm in length.

Our staining protocol also allowed us to visualize the brain and some of the cranial nervous system. From anterior to posterior, the largest part of the brain is the cerebrum (Figure 13; also known as the telencephalon), which is responsible for a wide range of functions, from integrating sensory information to the formation of memory and regulation of bodily functions (Naumann et al., 2015). We were not able to identify the olfactory bulbs that stem from the cerebrum in either specimen, which are known to be tiny in chameleons compared to other reptiles (Shanklin, 1930). The optic nerves are well developed and connect the eye to the optic tectum, the main visual processor of the brain (Figure 13c,d). In *C. calyptratus*, the cerebellum, which is important for maintaining balance and fine‐tuning motor control while traversing their arboreal habitat (Anderson & Higham, 2014), has the same shape as in other chameleons. Finally, the medulla oblongata connects the brain to the spinal cord and is responsible for several functions of the autonomous nervous system.

The position of the trigeminal nerve (V) between major cranial muscle groups is fairly conserved across diapsids (Daza et al., 2011; Holliday & Witmer, 2007). We were able to extract two of its three branches—the mandibular (V_3_) and maxillary (V_2_) nerves (Figure 13c). The maxillary nerve is identifiable based on its lateral position relative to the PS, while the mandibular nerve courses vertically between the medial side of AP and the lateral side of PT‐d bundle a.

### 3.4 Bite force and mechanical advantage

Estimated bite force in both sexes increases as the bite point moves from anterior to posterior, as is expected from lever arm mechanics. However, depending on the bite point, the estimated bite force is between 4 and 4.8 times higher in the male than in the female (Table 4), the discrepancy being most pronounced during anterior bites. In contrast, mechanical advantage—the ratio of output bite force to input muscle force—is very similar at corresponding bite points between the two sexes, being slightly higher in the male during anterior bites, identical between the male and female during middle bites, and slightly higher in the female during posterior bites. Even when the female skull was scaled to the same width as the male skull, bite force in the male was still between 2.6 and 3.1 times higher than in the female.

**TABLE 4:** Estimated bite forces (BF) in Newtons (N) and mechanical advantage (MA) for male and female *Chamaeleo calyptratus*.

Specimen	Anterior BF (N)	Middle BF (N)	Posterior BF (N)	Anterior MA (N)	Middle MA (N)	Posterior MA (N)
Male	60.37	79.83	114.87	0.32	0.42	0.61
Female	12.69	19.36	28.47	0.28	0.42	0.62
Female (scaled)	19.52	29.77	43.77	0.28	0.42	0.62

## 4 DISCUSSION

Our digital dissection of PMA‐stained specimens has enabled us to visualize and describe chameleon cranial anatomy in more detail than ever before. While the skeletal anatomy of chameleons besides *C. calyptratus* has been relatively well described for most regions of the skull (Anderson & Higham, 2014; Čerňanský et al., 2014; Iordansky, 2016; Rieppel, 1981, 1987, 1993; Villa & Delfino, 2019), we have provided novel insights into the different types of cranial sutures, and confirmed the presence of epipterygoid bones in chameleons. We have investigated the soft tissue anatomy, which lacks study for chameleons in general (Rieppel, 1981), describing individual muscle bundles in detail, as well as part of the nervous system. By exploring both a male and female specimen, we also provide evidence of *C. calyptratus* as a strongly dimorphic species. Considering both inter‐ and intraspecific differences in cranial morphology, we begin to interpret what factors are influencing cranial function in *C. calyptratus*. As this species is a promising model organism for the study of reptile development and evolution, we also discuss how academics can utilize these valuable anatomical atlases to facilitate future research and education.

### 4.1 Differences in cranial anatomy between *C. calyptratus* and other chameleon species

While our descriptions of *C. calyptratus* correspond with some of the previous literature on chameleons (Anderson & Higham, 2014; Rieppel, 1981; Villa & Delfino, 2019), we found several key differences in both the skeletal and muscular anatomy compared to other species, including others in the genus *Chamaeleo*. The largest difference is the dorsally enlarged casque (Figure 1; Measey et al., 2009). As previous literature has only suggested (Vanhooydonck et al., 2007), we find that the greater size of the casque is affiliated with larger jaw adductor muscles, especially AMEM‐a, AMEM‐c, and AMEP‐3b (Figure 8). It is also worth noting that AMEP‐3b does not expand anteriorly to fill the entire lateral view of the casque, in support of Haas (1973) and contrary to Rieppel (1981).

Beyond the casque, we observed several differences in the cranial anatomy of *C. calyptratus* compared to other species. In terms of the skull, premaxillary vestigial teeth are absent in these adult specimens, unlike in *C. chamaeleon* ([*vulgaris*]; Rieppel, 1981). In addition, we found evidence of the presence of epipterygoids, contrary to previous studies (Evans, 2008; Iordansky, 2016; Villa & Delfino, 2019), and found that the stapes are better developed than expected, as they are said to be reduced in size to the point of non‐functionality in chameleons (Anderson & Higham, 2014). In terms of muscle anatomy, our descriptions are novel in that the AMEM could be consistently divided into four distinct bundles (Figure S8), and the deep layer of PT could be subdivided into two bundles (Figure S12). We also describe the tendon associated with the insertion of the PS muscles (Figures 12 and S11) and find that PS superficialis does not spread antero‐posteriorly across the entire casque, in support of Haas (1973) and contrary to Rieppel (1981).

These interspecific differences in cranial anatomy could be partly influenced by differences in habitat, social behavior and/or diet. Chameleons occupy a broad variety of habitats (Measey et al., 2014), even within the same species, resulting in multiple ecomorphs (e.g., *Bradypodion*; da Silva et al., 2014; Measey et al., 2009). While individuals occupying closed habitats (e.g., forests) are associated with larger casques, previous studies suggest they only exhibit higher bite forces when the casque is not corrected for body size, suggesting that it is an ornamental trait that does not contribute toward bite force directly (Measey et al., 2009). *C. calyptratus*, which resides in the vegetated areas of the Arabian Peninsula (Wilms et al., 2012), has the most enlarged casque of any chameleon. A preliminary investigation by Vanhooydonck et al. (2007) found that relative crest height and relative bite force have a significant, positive correlation, while Ligon and McGraw (2018) showed that casque width was the best predictor of male bite force in *C. calyptratus*. This has been linked to their strong territorial behaviors (often resulting in male–male aggression), and to intraspecific communication behaviors. In male chameleons, casque size is considered an honest signal for fighting performance, often preventing costly encounters, and its coloration is involved in courtship behaviors that display willingness to mate (Ligon & McGraw, 2018; Stuart‐Fox et al., 2006). Casque height in *Bradypodion* has also been positively associated with male fighting ability (Stuart‐Fox et al., 2006). Finally, diet in other species of chameleon has not been linked to the use of higher bite forces (Measey et al., 2011), and males and females did not demonstrate significant differences in dietary preferences (e.g., harder prey; da Silva et al., 2016). To make more direct comparisons of musculoskeletal anatomy and function between species, feeding performance needs to be quantified using laboratory analysis and/or biomechanical modeling.

### 4.2 Sexually dimorphic cranial anatomy suggests that males are adapted for generating higher bite forces

The male skull was 7.2% wider relative to mandible length compared to the female (Table 2), which corresponds with findings for *Bradypodion pumilum* (Measey et al., 2009), for which head width was shown to directly and positively contribute to increased bite force. Additionally, the largest morphological disparity between the sexes is found in the facial skeleton and cranial casque (Table 1; Figures 1 and 2). Notable differences in the facial skeleton include the relatively larger orbit, external naris and prefrontal fenestra in the female compared to the male (Table 2). In general, the male seems to have a more robust skeletal morphology than the female (Table 1), with the exception of the base of the parietal (Figure 1b).

Total skull height is 33.2% taller relative to mandible length in the male compared to the female. This is largely driven by the male casque, which is 11.5% taller relative to total skull height than the female. The male casque is also less curved and oriented more dorsally (Figure 1b). As suggested by previous studies (Vanhooydonck et al., 2007) but quantitatively demonstrated here, the enlarged casque of the male provides more extensive muscle attachment sites (Figure 11) and more space to house substantially larger jaw adductor muscles, particularly AMEM‐a, AMEM‐c, and AMEP‐3b (Figures 8 and 9). AMES and AP, muscles within the same externus complex, are also larger with more extensive attachment sites on the skull in the male (Figures 9 and; Figure 11). Even when the female skull is scaled to the same width as the male's, nearly all of the jaw closing muscle volumes are still substantially smaller than those of the male (with the exception of AMEM‐b), demonstrating that changes in skull shape (not merely increased skull size) are accommodating larger muscles in the male.

Larger jaw closing muscles in the male (nearly five times larger in terms of absolute volume; Supplementary Dataset) result in higher estimated bite forces (4–4.8 times higher) than in the female (Table 4). This may be related to prey capture and/or intraspecific aggression (Iordansky, 2016; Stuart‐Fox, 2014). Even when the female skull was scaled to the same width as the male skull, bite forces in the male were still 2.6–3.1 times higher than in the female (Table 4), demonstrating that changes in skull shape—not simply increased skull size—allow for larger muscles in the male. Interestingly, mechanical advantage was similar in the two specimens at the same bite point, suggesting that changes in skull geometry do not impact muscle moment arms, and that increased bite force is primarily due to larger muscle size (but see Study Limitations for caveats). Additionally, the depressor mandibulae is larger with a broader area of attachment in the male (Figures 9 and 11), so it is likely that the male can generate more force during jaw opening than the female. The larger size of the pterygoideus in the male, especially the superficial region (Figure 9), is likely linked to sexual selection to make the head appear wider, as its contribution to bite force is relatively minimal (Herrel et al., 1999).

### 4.3 First description of chameleon suture morphology provides novel insights into intraspecific differences in skull loading

This study presents the first detailed description of suture shape in chameleons, providing insights into how the skull might be loaded. Given the lack of previous literature, the only comparisons we can confidently make to other species are that the nasals are not fused in *C. calyptratus*, unlike *Bradypodion* and *Brookesia*, and like other species of *Chamaeleo*, the dorsal tip of the jugal meets the squamosal (Anderson & Higham, 2014). Additionally, the premaxilla has a ventral process that underlaps the vomer, unlike *Bradypodion*.

There are, however, differences in suture shape between the male and female that enable us to predict differences in skull loading regimes between the sexes. Experiments and computer modeling have linked certain suture shapes with specific types of loads: butt joints are usually associated with tension or bending; interdigitated sutures are associated with compression, with more pronounced interdigitations linked to increased loads; tongue and groove joints are associated with resistance to tension; and scarf/lap joints have been linked to tension, compression, torsion and/or shear, with some authors suggesting these contacts represent a functional compromise to resist complex loading regimes (Busbey, 1995; Herring & Mucci, 1991; Herring & Teng, 2000; Markey et al., 2006; Rafferty & Herring, 1999). Both the male and female exhibit butt joints at the midline of the anterior snout, between the bones of the braincase, and at the back of the skull. The bony pillar posterior to the orbit formed by the squamosal, postorbital and jugal exhibits stepped and tongue and groove joints. The prevalence of these contacts suggests tension is the dominant loading regime in these areas, potentially generated by muscle action or twisting of the skull during unilateral bites. In contrast, the sides of the anterior snout and center of the skull roof are dominated by interdigitated contacts, suggesting these regions are adapted to resist compression, possibly channeled upwards from the tooth row during biting. The lower jaw features extensive lap and scarf joints, suggesting resistance to complex loading regimes. This corresponds with data from in vivo strain gauge experiments in crocodilians that demonstrate the elongate, beam‐like lower jaw is subjected to bending, shear and torsion during biting (Porro et al., 2013). Future biomechanical modeling of the upper and lower jaws will allow testing of these hypotheses regarding the distribution of different types of stress/strain during biting in *C. calyptratus*.

There are also differences in suture shape between the sexes. The male shows stronger interdigitations and deeper slot joints in the cranium, a slot joint in place of a butt joint in the facial skeleton, and more extensive lap joints in the lower jaw, suggesting that the male skull is more strongly consolidated than the female in several areas (Table 1). This could reflect an adaptation to resisting higher forces generated by larger muscles, and resulting higher bite and jaw joint reaction forces in the male, hypotheses that will be tested in upcoming biomechanical modeling. The palate shows mixed evidence for this, in that the male has a deeper ectopterygoid‐pterygoid slot joint, but the palatines are much more interdigitated and the stepped joints between the vomer and its surrounding bones are more pronounced in the female (Figure 4b). Differences in joint distributions could also relate to the size difference between the male and the female. Studies on how suture type distribution changes with growth would provide valuable insight on this relationship.

### 4.4 Study limitations

The greatest limitation of this study is sample size, as we report the anatomy of only one male and one female specimen in detail. Thus, we cannot clearly identify which fine‐scale anatomical differences (e.g., number of crests on the jugal, shape of tendons) represent intraspecific variability. Any quantitative measurements provided here should therefore be used only as a guideline and will likely vary across the taxon. However, our anatomical descriptions closely match those observed through decades of traditional dissections (AH, pers. obs.) and are largely in line with descriptions for other species of *Chamaeleo* (Herrel et al., 2001; Rieppel, 1981). Furthermore, while we could not compare hyolingual anatomy between the sexes due to insufficient scan resolution at the anterior end of this region for the male specimen, the digital atlas of the female provides a 3D visualization of these structures, complementing detailed descriptions in Herrel et al. (2001). This could be particularly useful for future studies of intraspecific communication, since the hyoid muscles are involved in the generation of biotremors (Tegge et al., 2020).

Our small sample size is due to the time‐consuming and labour‐intensive process required to create comprehensive digital atlases, despite the recent advances in automated segmentation techniques we utilized (Didziokas et al., 2024). This is partly due to our choice to use PMA over diffusible iodine, which is known to have a slower diffusion rate (Pauwels et al., 2013), so that we could visualize more types of soft tissue, such as tendon and cartilage. It is also worth noting here that while PMA has been shown to have a higher incidence of muscle shrinkage than iodine staining (Balint et al., 2016), we used a concentration of 2.5%, which is low enough to show no noticeable changes in muscle volume (Pauwels et al., 2013). Furthermore, both specimens went through the same protocol, so this would not inhibit our ability to compare the anatomy of the male and female specimens.

Our bite force estimates (Table 4)—peak posterior bite forces of 114.87 N for the male (mandible length of 43.93 mm) and 28.27 N for the female (mandible length of 33.77 mm)—were comparable to those measured in vivo from individuals of similar lower jaw lengths (122.87 N and 54.13 N, respectively) by Drown et al. (2022) but markedly lower than forces measured by other workers. Ligon and McGraw (2018) reported in vivo bite forces of 238.24 N for an individual with a lower jaw length of 44 mm while AH measured in vivo bite forces of 249.42 N for a similarly sized male (mandible length of 42.49 mm) and 76 N for a similarly sized female (mandible length of 32.74 mm) (AH, pers. comm.). Thus, the bite forces calculated here using muscle volumes and total muscle lengths measured from stained μCT data (applying published corrections for fiber lengths) and 3D lever arm mechanics are substantially lower than values reported from some in vivo bite force experiments. Our imaging data clearly show that the jaw‐closing muscles of both individuals are highly pennate, which would result in substantially increased muscle cross‐sectional area (*physiological* cross‐sectional area, or PCSA) compared to our calculations. This is the most likely explanation for the lower‐than‐expected bite forces calculated in this study. Therefore, the values reported here should be seen as a conservative estimate of maximal bite force in individual *C. calyptratus* of this size.

Furthermore, despite the substantially taller casque of the male, we found that mechanical advantage was nearly identical between the sexes, suggesting the larger casque does not change muscle moment arms. However, we emphasize again that our results are based on only one individual of each sex. As part of a larger study, we are aware of exceptionally large females that feature enlarged casques that are oriented posteriorly (i.e., less vertically) than those of large males. However, these very large females were not available for staining. Future work will investigate how differences in the orientation of the casque between the very largest males and females may impact jaw mechanics in this species.

### 4.5 Conclusions

We have provided novel insights into major anatomical features that cannot be easily explored with physical dissection, such as suture types, 3D muscle relationships, and subtle boundaries between different bundles within muscle complexes. To facilitate more direct comparisons with other chameleon species, future studies should use our atlases and qualitative descriptions of interspecific differences as a foundation to describe cranial anatomy and function. Comparative studies of soft tissues are particularly lacking. This, combined with measurements of bite force and/or biomechanical modeling, will provide better insight into the functional consequences of differences in cranial anatomy in chameleons. Furthermore, while changes in the cranial skeleton across ontogeny have been examined previously in other chameleon species (Rieppel, 1993) and in the lower jaw of *C. calyptratus* (Kavková et al., 2022), it is unknown how the cranial muscles change throughout development. We hope that the workflow and anatomical atlases presented here inspire a new age of studies of reptilian anatomy and development.

By making interactive versions of our anatomical atlases available online, we have also contributed toward making the anatomical sciences more accessible to researchers, biosciences and veterinary students, educators at all grade levels, museums and the general public. Alongside this manuscript, we have published two lesson plans aligned with the UK science curriculum (one aimed at GCSE level students, one at first‐year university undergraduates) that only require a computer and internet access. They aim to guide educators and their students through digital dissections that will deepen students' understanding of biology and comparative anatomy and facilitate the development of practical skills that are relevant to modern scientific research.

## AUTHOR CONTRIBUTIONS

**Alice Leavey:** Writing – review and editing; writing – original draft; data curation; investigation; visualization; methodology; validation; formal analysis. **Eloy Gálvez‐López:** Conceptualization; writing – review and editing; data curation; validation; methodology; investigation. **Anthony Herrel:** Writing – review and editing; data curation; validation. **Laura B. Porro:** Conceptualization; funding acquisition; writing – review and editing; project administration; resources; supervision; methodology; validation; formal analysis; data curation.

## FUNDING INFORMATION

This project was supported by a UKRI‐funded Future Leaders Fellowship (MR/W011484/1) to LBP.

## CONFLICT OF INTEREST STATEMENT

The authors declare no conflict of interest.

## Supporting information


**Data S1:** Supplementary Information.

**Data S2** Understanding comparative anatomy using 3D digital models.

**Data S3** Supplementary Dataset.

## Data Availability

All the data associated with this manuscript are available in the supplementary material. The microCT scans can be found on MorphoSource (https://www.morphosource.org/projects/000752255?locale=en), while the 3D digital atlases can be visualized on SketchFab (https://sketchfab.com/aleavey/collections) and downloaded directly from Figshare (https://figshare.com/projects/3D_anatomical_atlas_of_the_heads_of_male_and_female_adult_Chamaeleo_calyptratus/263071).
