# Supplementary material for: 3D anatomical atlas of the heads of male and female adult Chamaeleo calyptratus
Source: Anat Rec (Hoboken). 2025 Nov 15;309(8):1937–69. doi: 10.1002/ar.70077 (PMC13331541; doi:10.1002/ar.70077)
Supplement: Supplementary file 1 — Data S1: Supplementary Information. [file AR-309-1937-s001.docx]

**Supplementary figures for: Leavey, A., Gálvez-López, E., Herrel, A. & Porro, L.B. 3D anatomical atlas of the head of male and female *Chamaeleo calyptratus.***


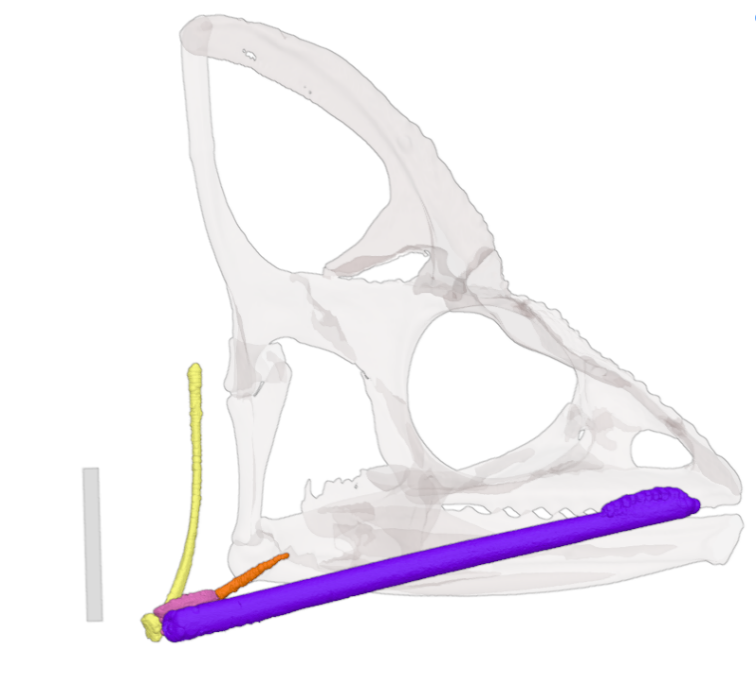


Ceratobranchial

Ceratohyal

(distal – cartilaginous)

Entoglossal process

Ceratohyal

(proximal – ossified)

**Figure S1 –** The tongue skeleton of the female specimen from a medial view. The gray scale bar represents 10mm.


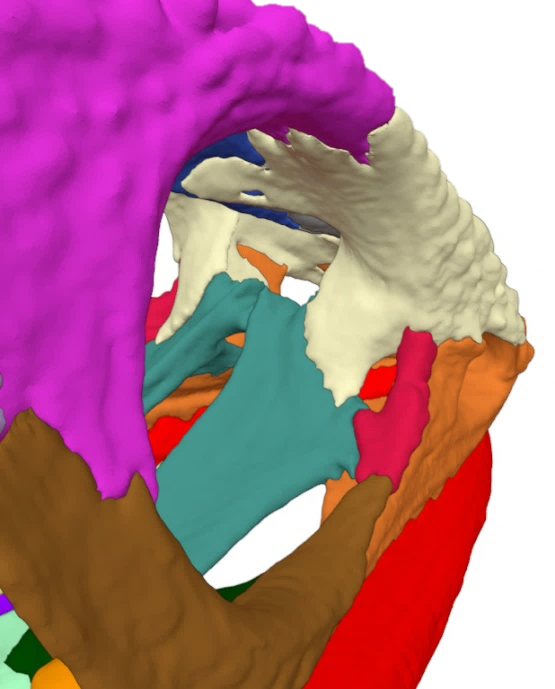


Infraorbital fenestra

Nasolacrimal canal

**Figure S2 –** An outline of the nasolacrimal canal and infraorbital fenestra of the male specimen from a posterolateral view of the right orbit (orange dashed line).


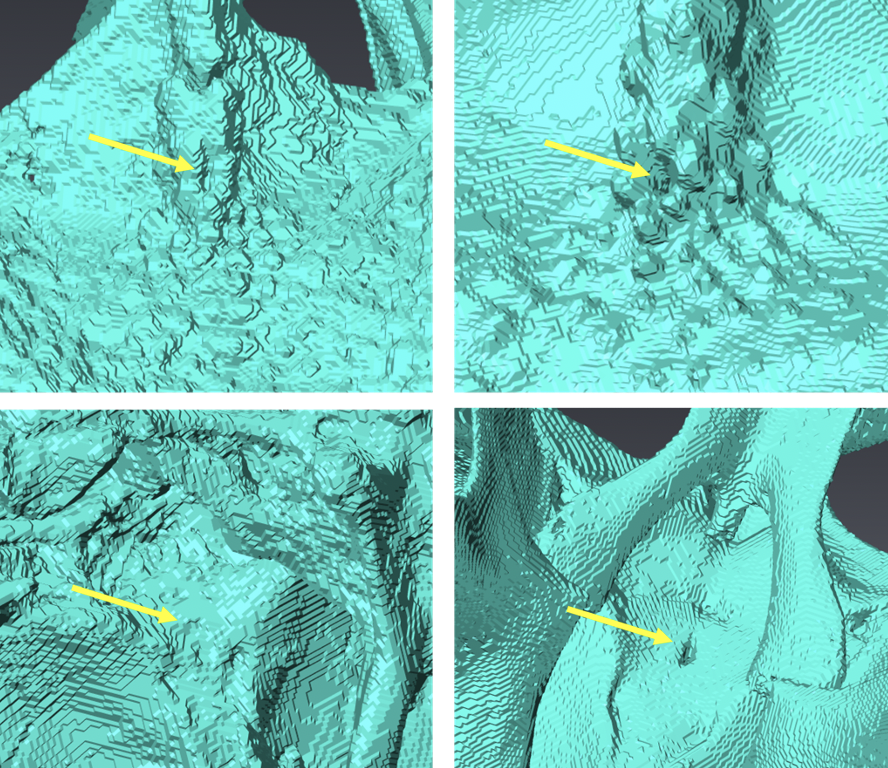


**Figure S3 –** The pineal foramen, indicated by the yellow arrow, in the male (left) and female (right) skull, in dorsal (top) and ventral (bottom) view.


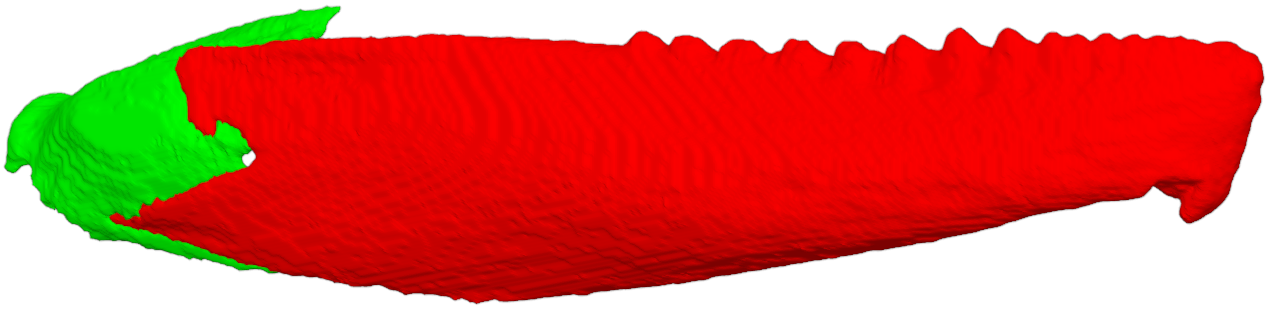


**Figure S4** – The lateral mandibular foramen (yellow circle) found at the contact between the dentary (red) and surangular (green) in the right lower jaw of the male. Note that this opening is homologous to the anterior surangular foramen of other lizards.

**Figure S5 –** The bodenaponeurosis in male (left) and female (right) *C. calyptratus* from a lateral (top) and medial (bottom) view. We could not visualise a lateral septa of the bodenaponeurosis in the female. The grey scale bars beside each figure represents 10mm in length.


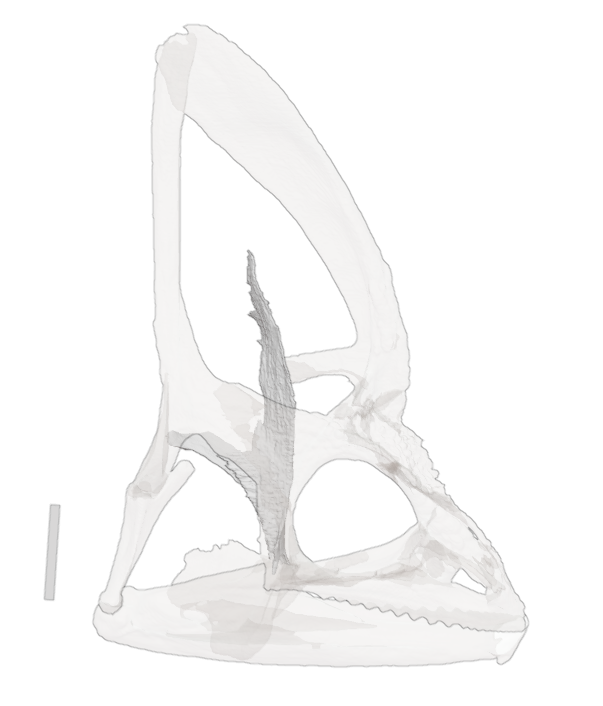

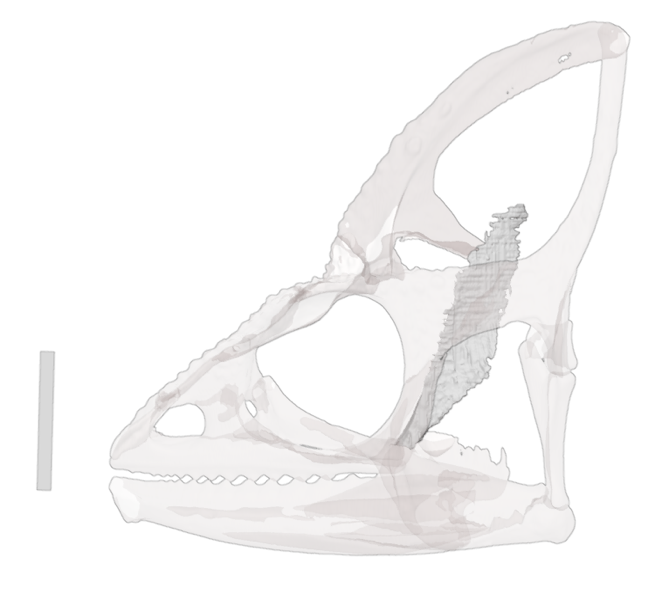

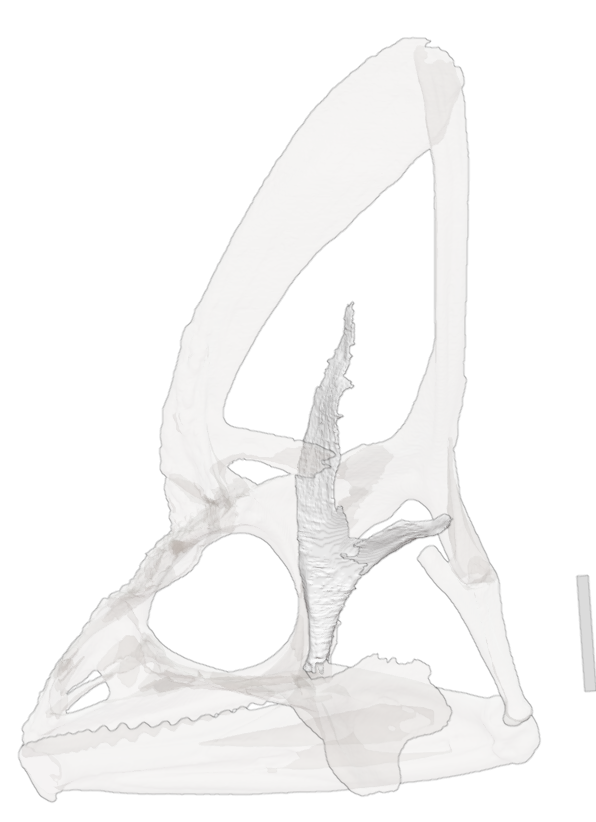

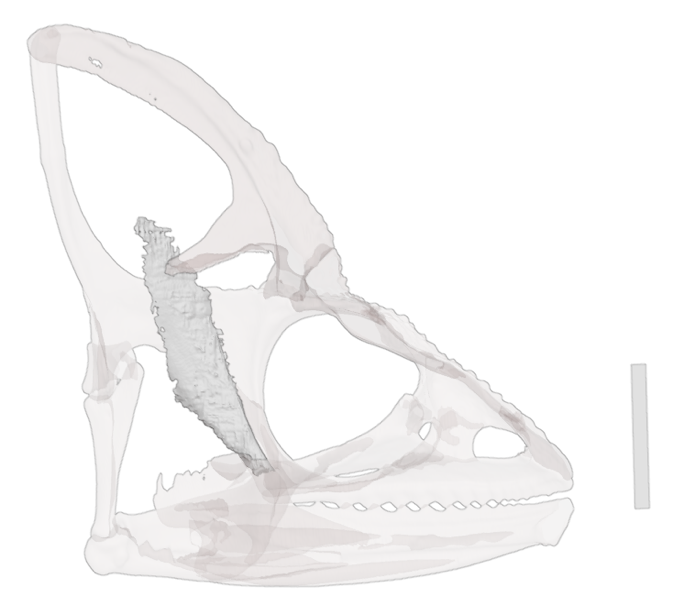


**Figure S6** – The levator anguli oris (LAO) and retractor anguli oris (RAO) muscles in male (left) and female (right) *C. calyptratus* from a lateral (top) and medial (bottom) view. In the female specimen, the scan resolution enabled this superficial anatomy to be further subdivided, showing where the LAO may end and rictal plate may begin. The grey scale bars beside each figure represents 10mm in length.


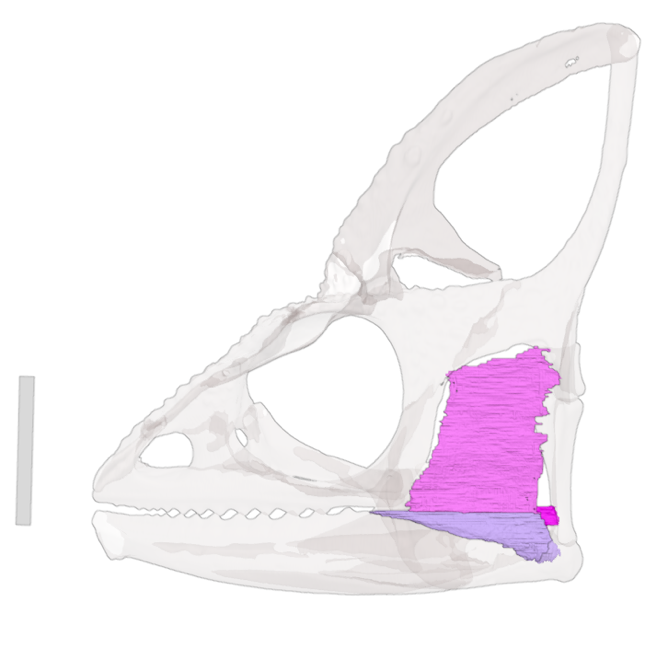

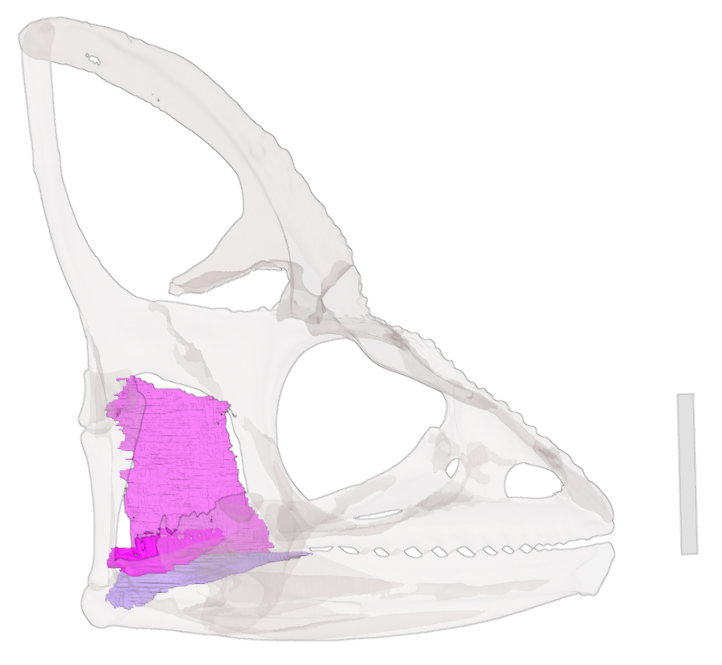

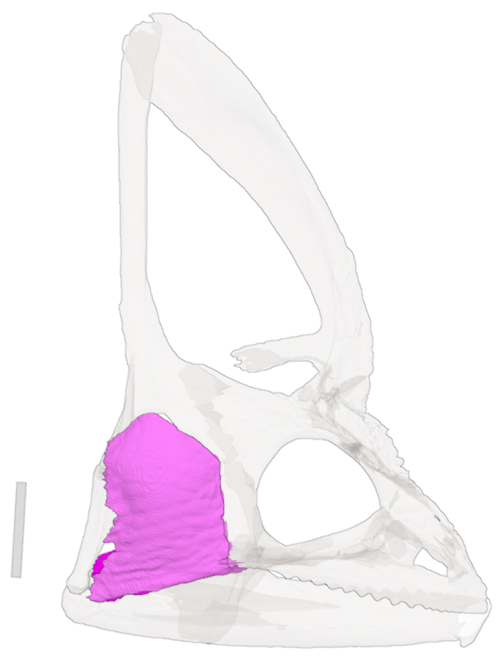

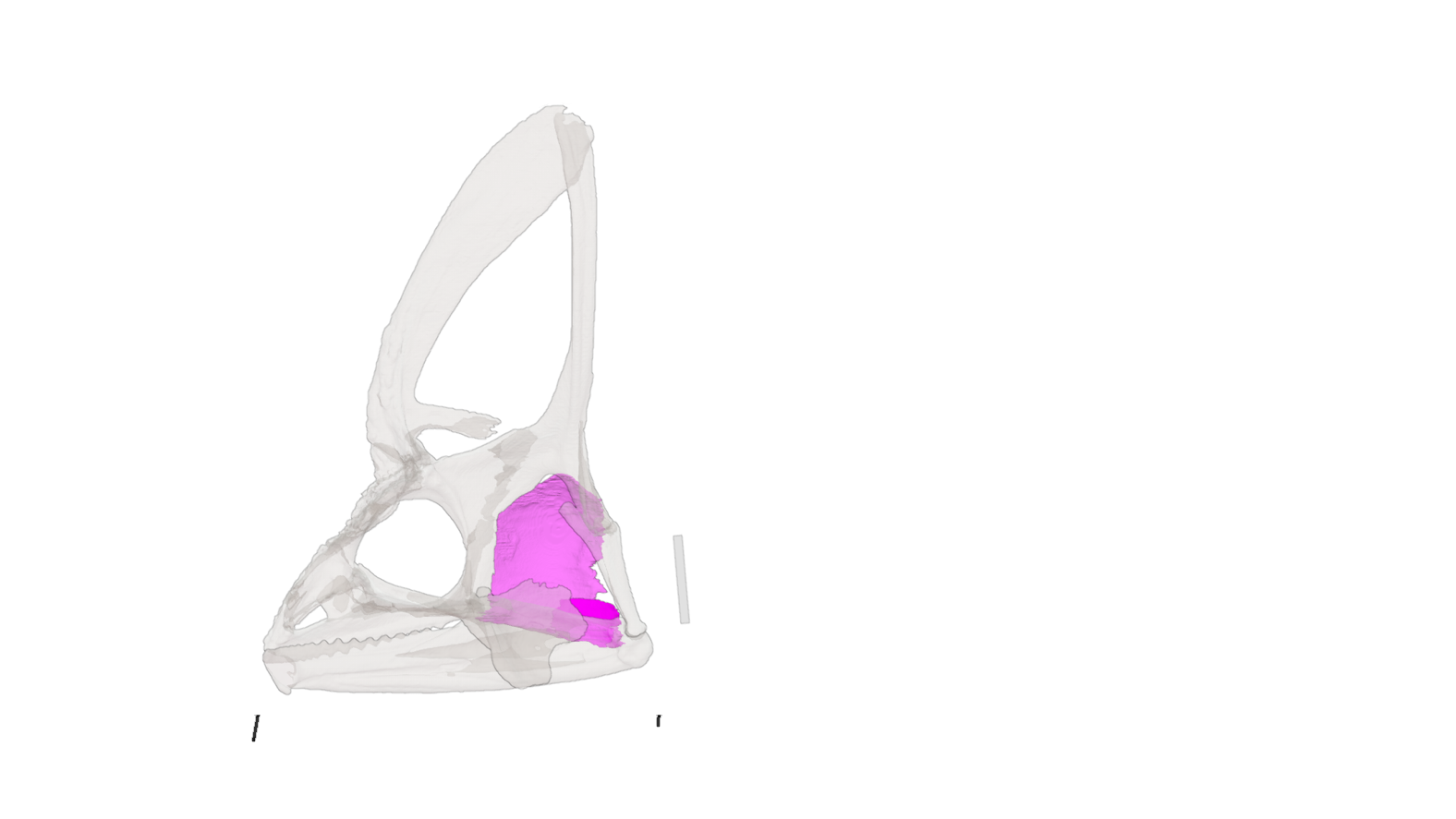


LAO + Rictal plate

LAO

Rictal plate

RAO


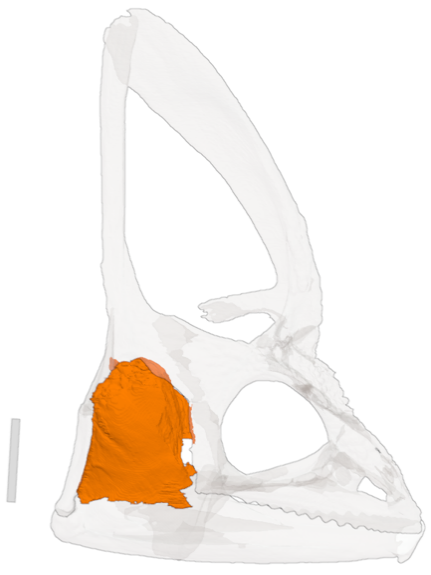

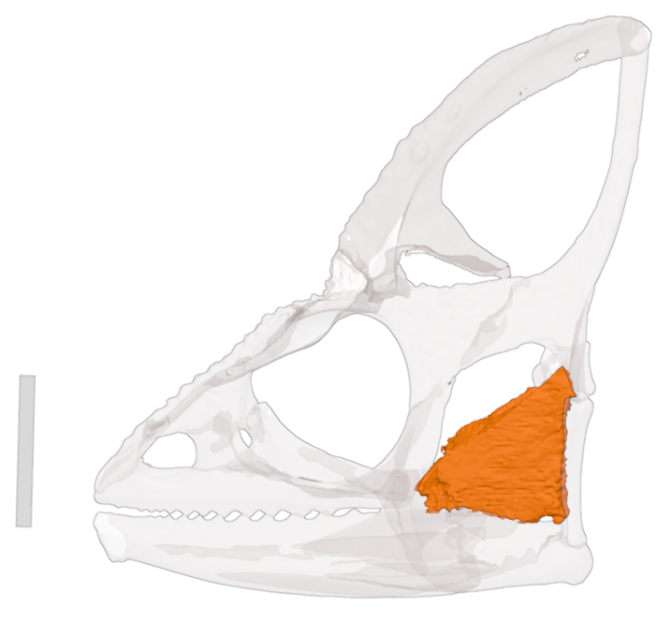

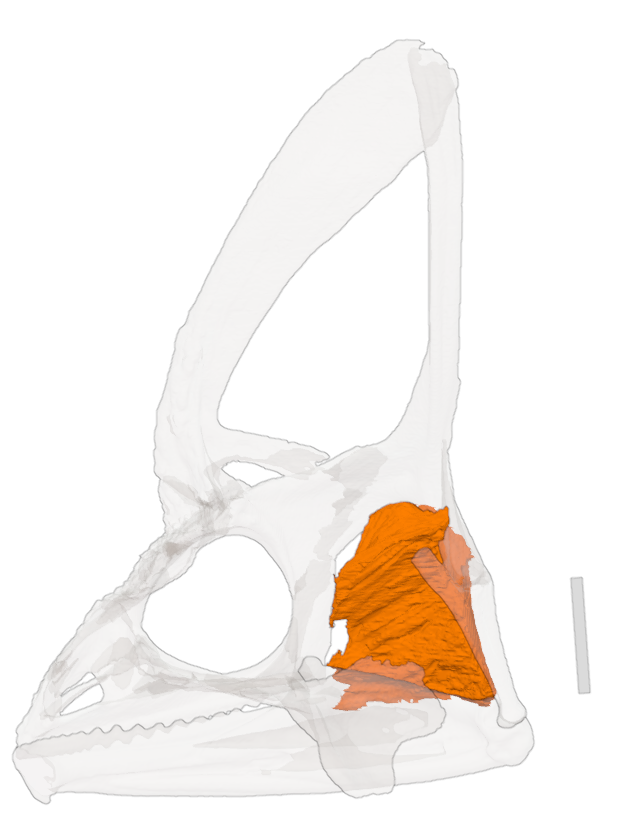

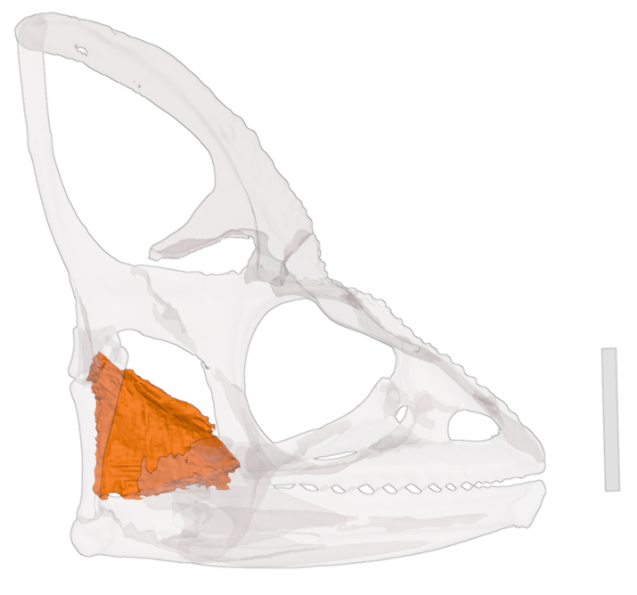


**Figure S7** - The adductor mandibulae externus superficialis (AMES) muscle in male (left) and female (right) *C. calyptratus* from a lateral (top) and medial (bottom) view. The grey scale bars beside each figure represents 10mm in length.


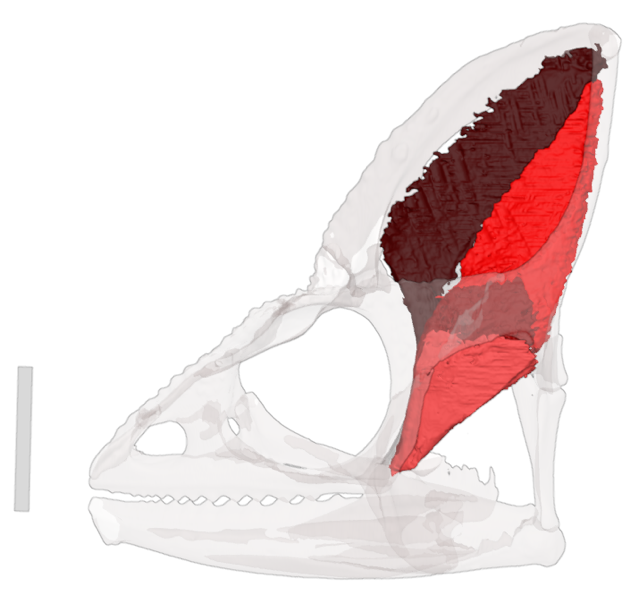

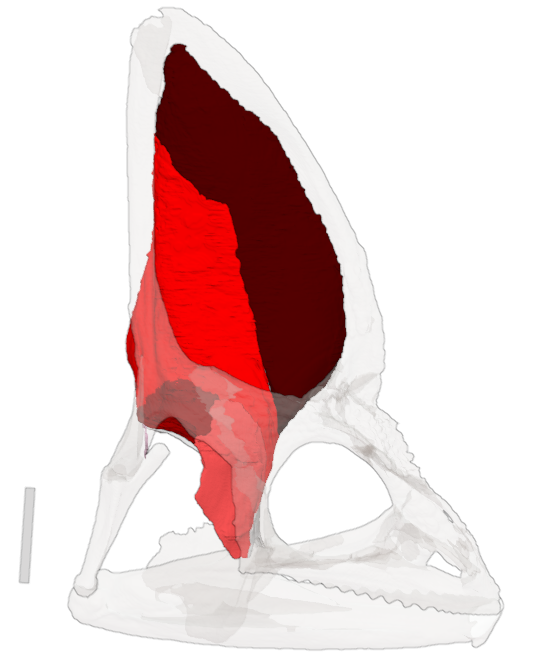

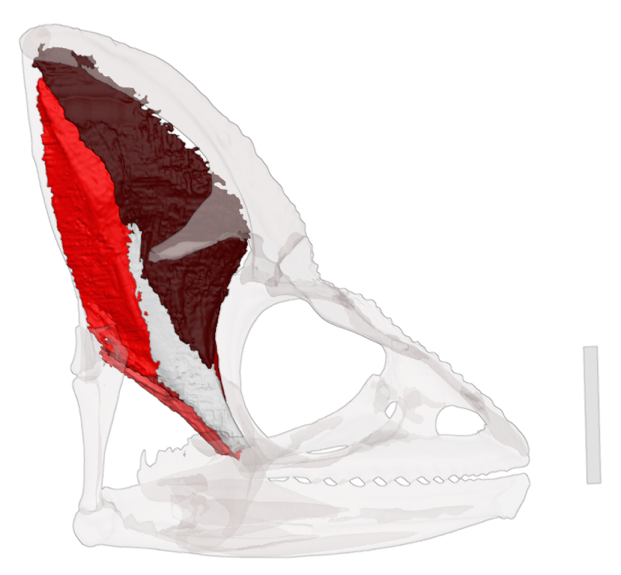

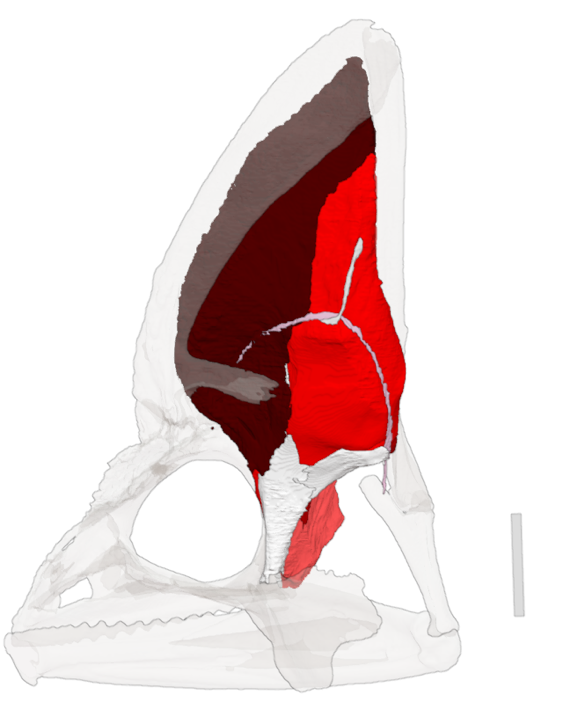


Bundle a

Bundle c

Bundle d

Bundle b

Artery

Tendon

Bodenaponeurosis

**Figure S8** - The adductor mandibulae externus medialis (AMEM) complex in male (left) and female (right) *C. calyptratus* from a lateral (top) and medial (bottom) view. The artery and small tendon were not able to be dissected well in the female specimen, but the bodenaponeurosis is present in both sets of figures. The grey scale bars beside each figure represents 10mm in length.

Bodenaponeurosis

Bundle 3c

Bundle 3b

Bundle 3a


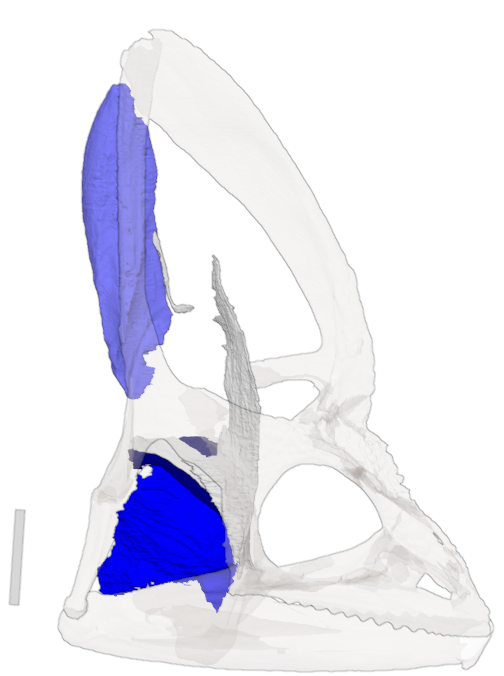

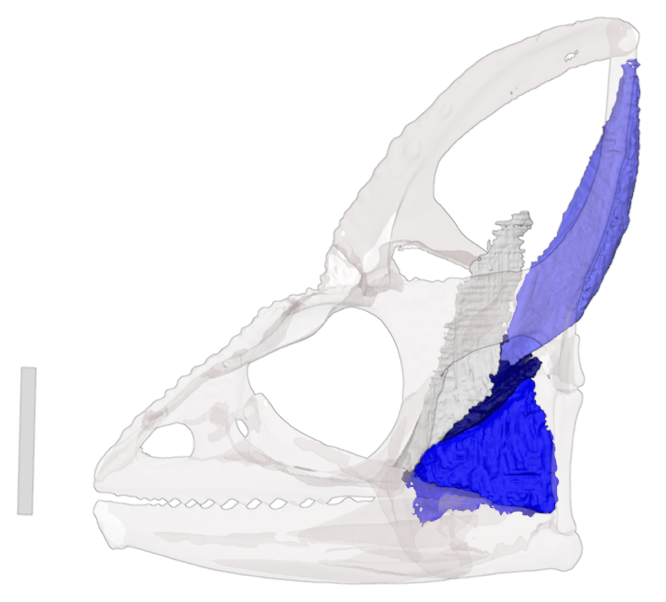

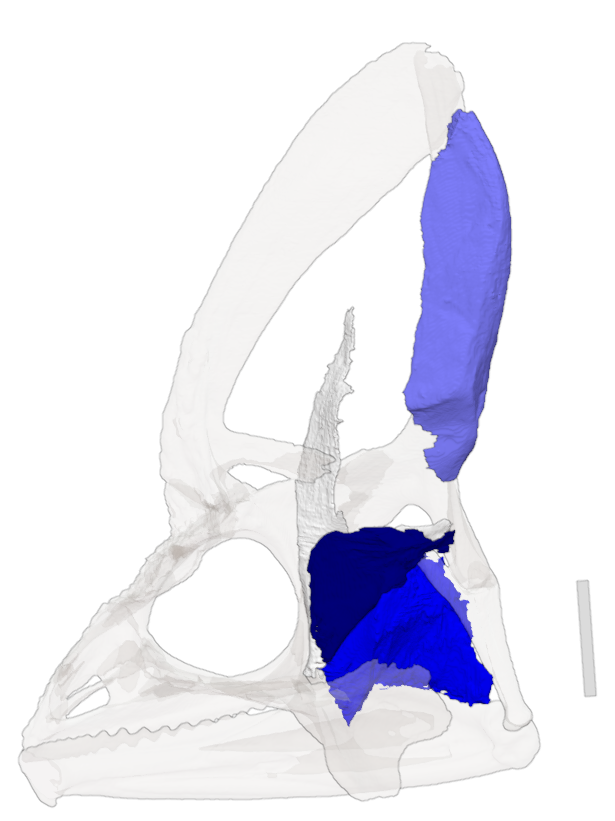

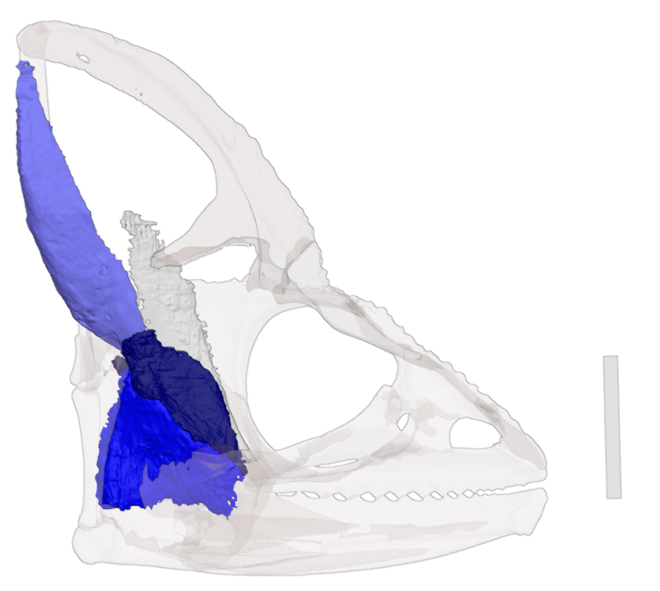


**Figure S9** - The adductor mandibulae externus profundus (AMEP) complex in male (left) and female (right) *C. calyptratus* from a lateral (top) and medial (bottom) view. The bodenaponeurosis is also present. The grey scale bars beside each figure represents 10mm in length.


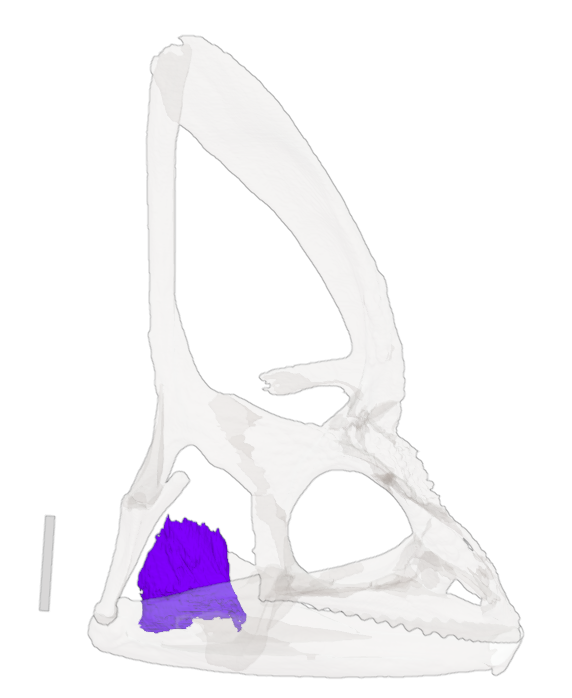

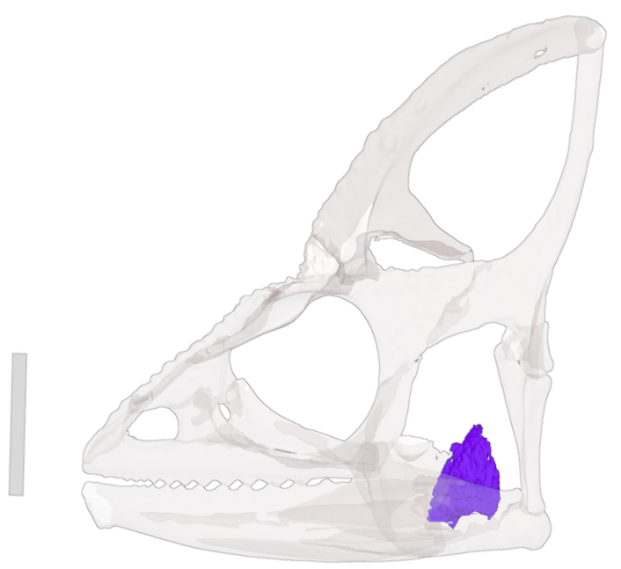

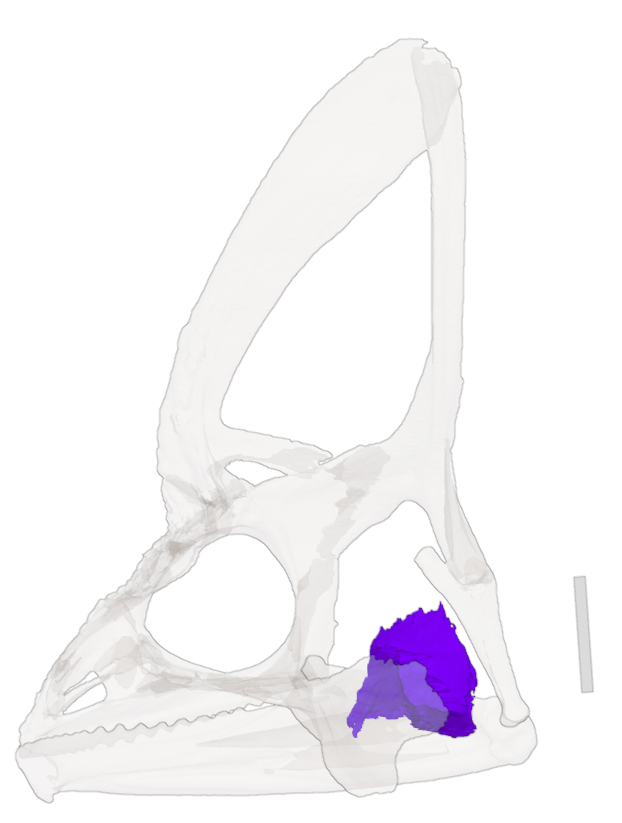

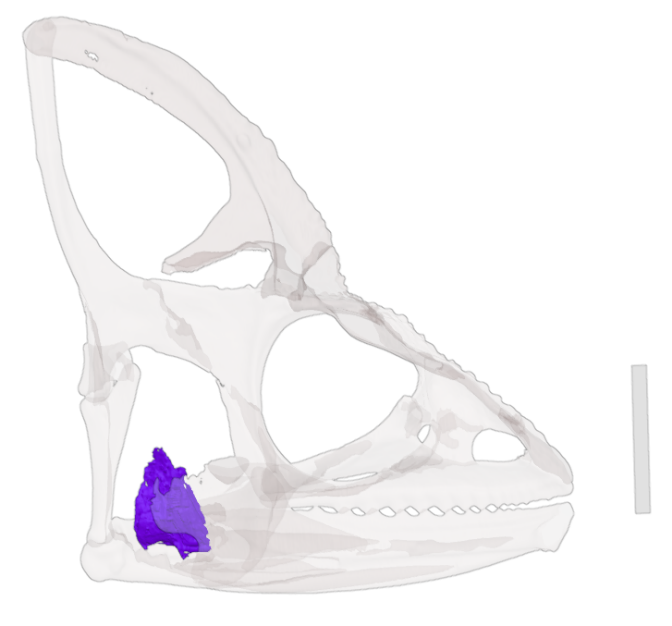


**Figure S10** - The adductor posterior (AP) in male (left) and female (right) *C. calyptratus* from a lateral (top) and medial (bottom) view. The grey scale bars beside each figure represents 10mm in length.


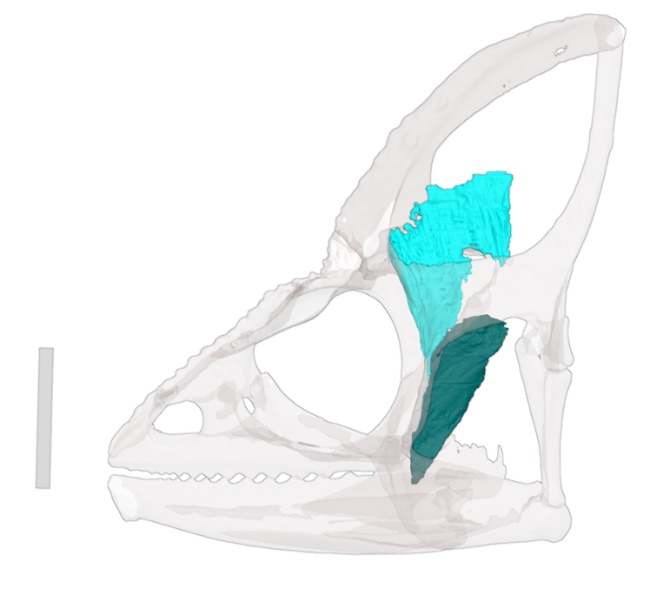

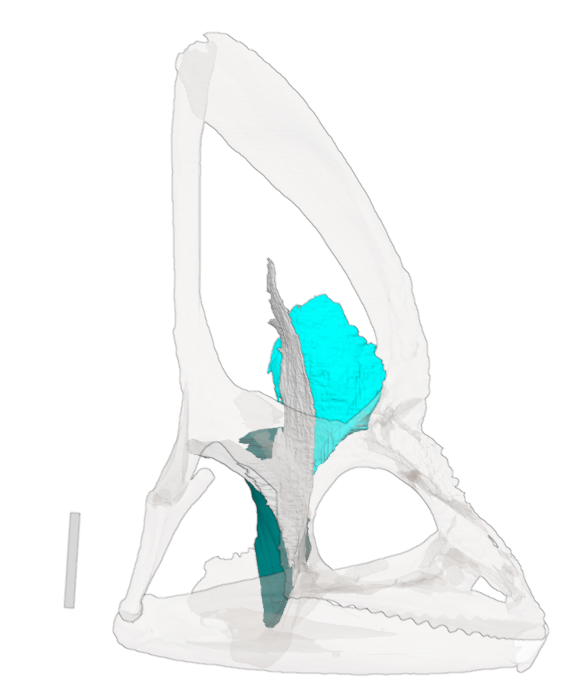

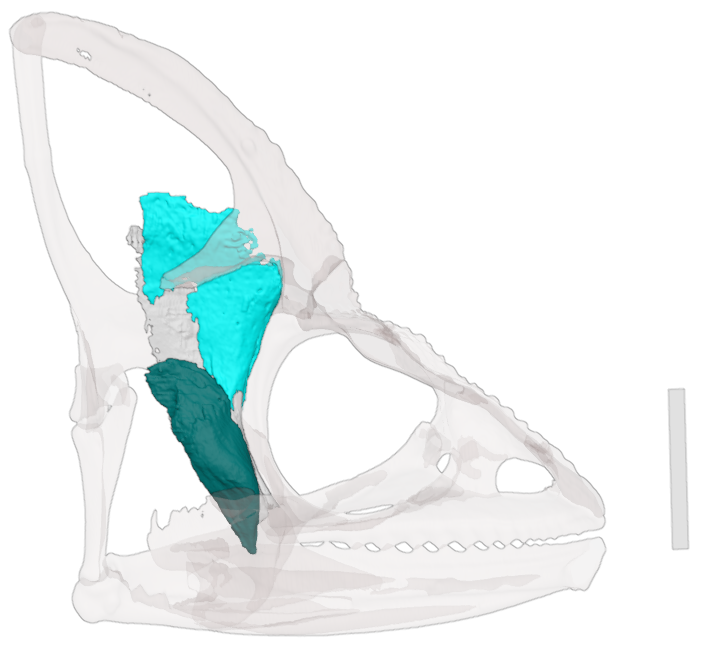

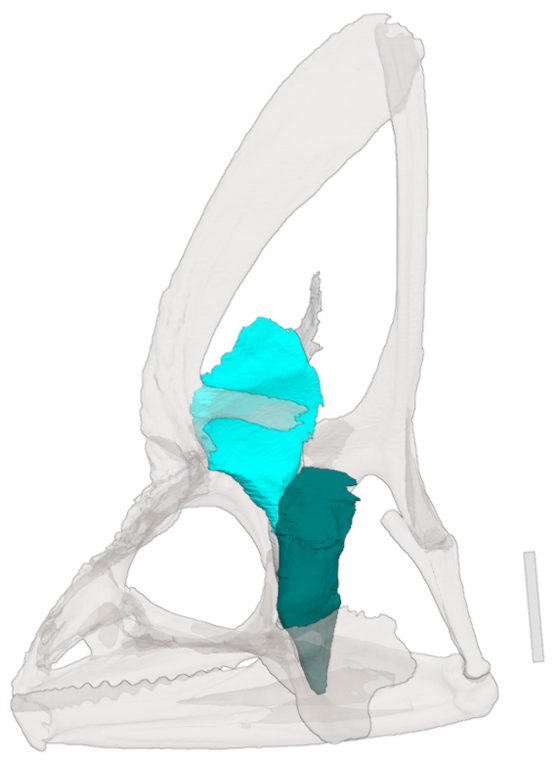

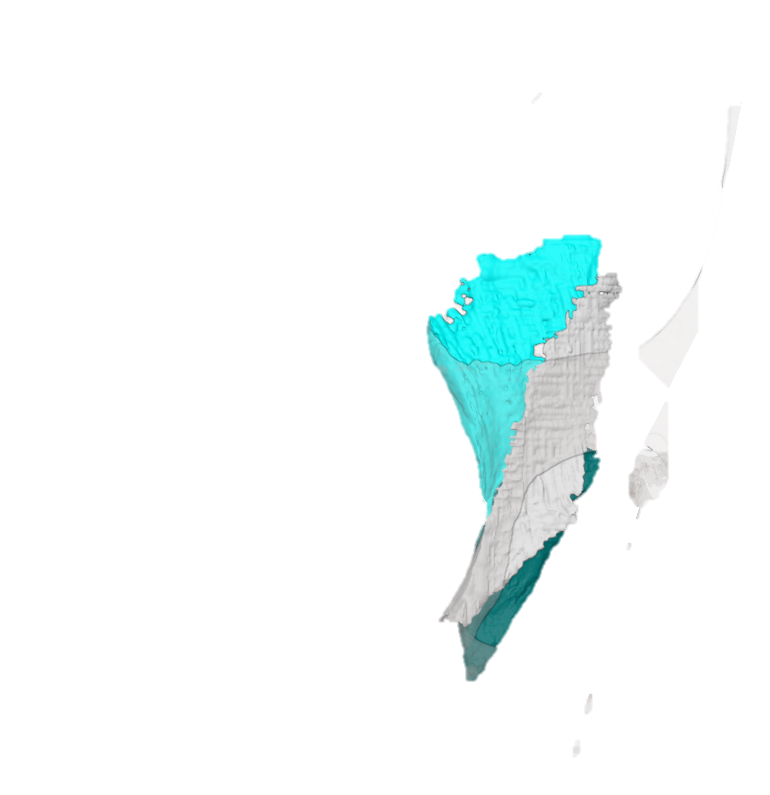


PS tendon

PS tendon

Bodenaponeurosis

PS profundus

PS superficialis

**Figure S11** - The pseudotemporalis (PS) muscles in male (left) and female (right) *C. calyptratus* from a lateral (top) and medial (bottom) view. The common insertion point of both heads is via a tendon visible in the medial view. The bodenaponeurosis is also present. The grey scale bars beside each figure represents 10mm in length.


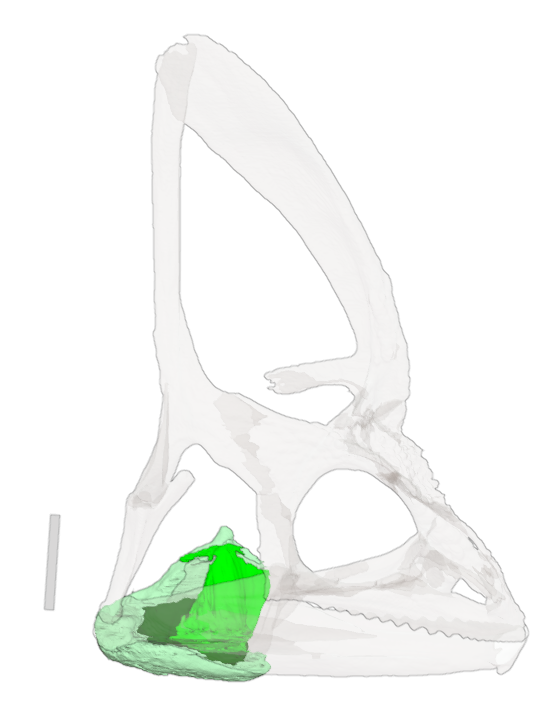

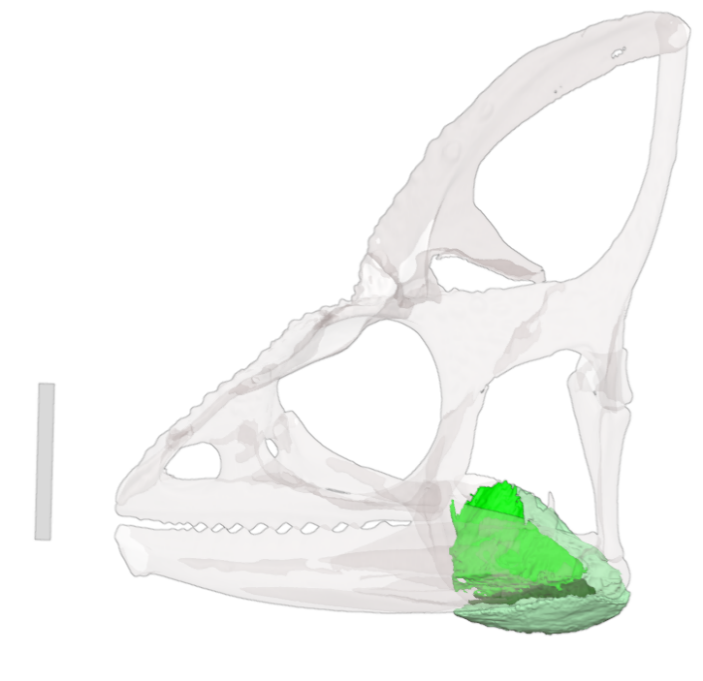

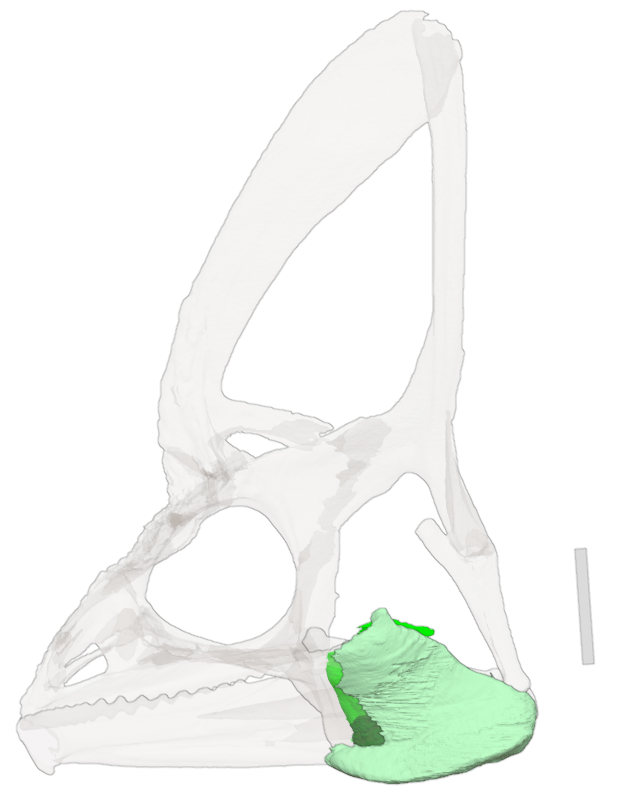

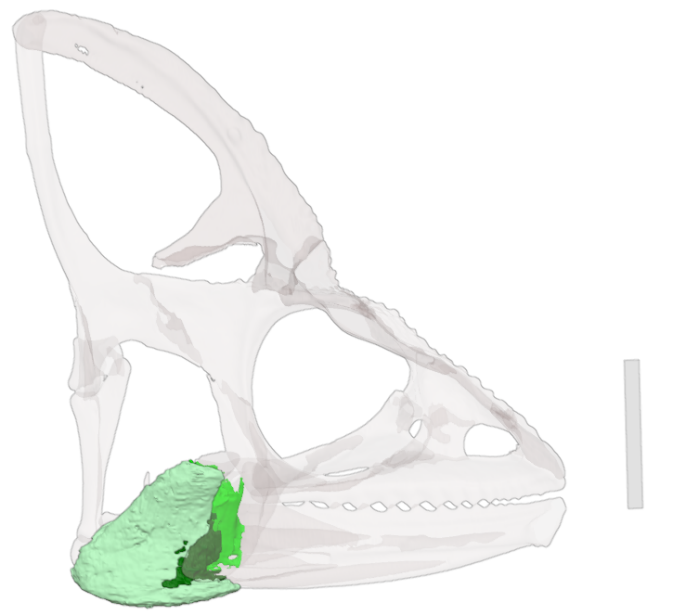


PT superficial

PT deep bundle a

PT deep bundle b

PT superficial

PT deep bundle a

PT deep bundle b

**Figure S12** - The pterygoideus (PT) muscle complex in male (left) and female (right) *C. calyptratus* from a lateral (top) and medial (bottom) view. The grey scale bars beside each figure represents 10mm in length.


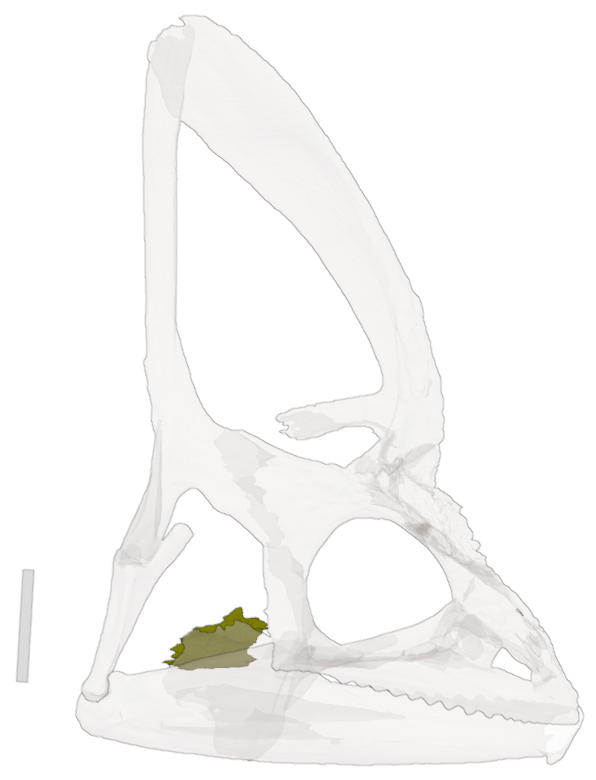

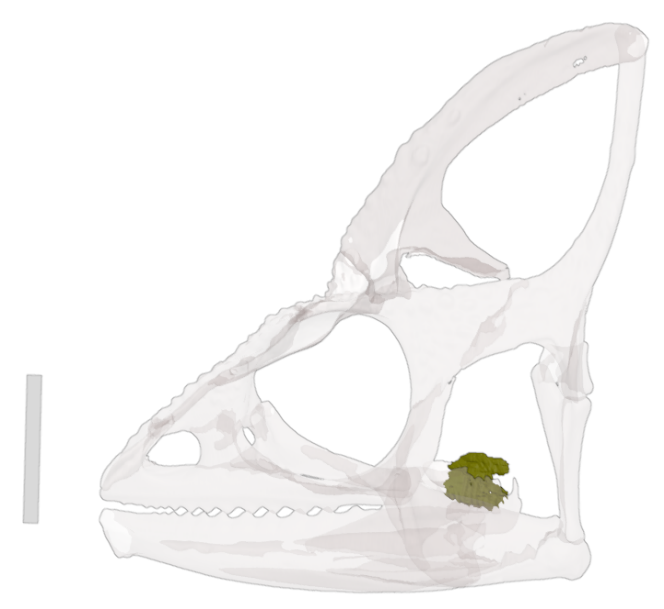


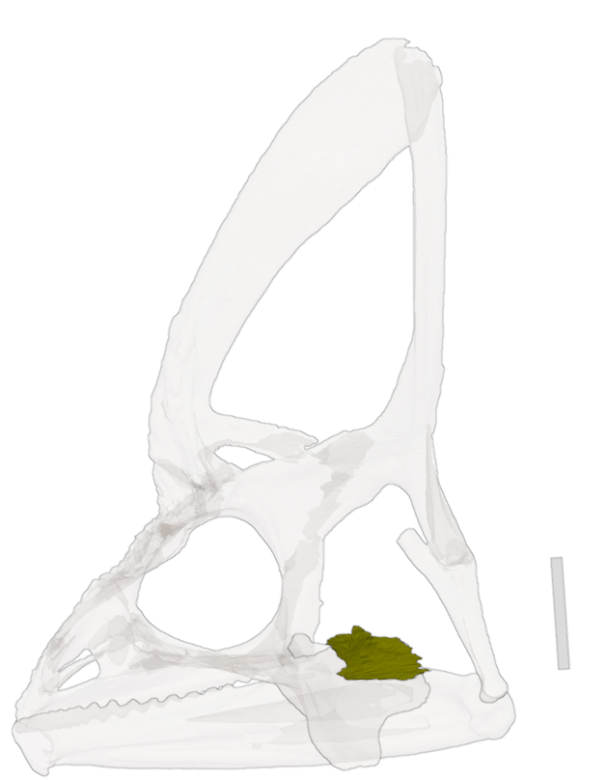

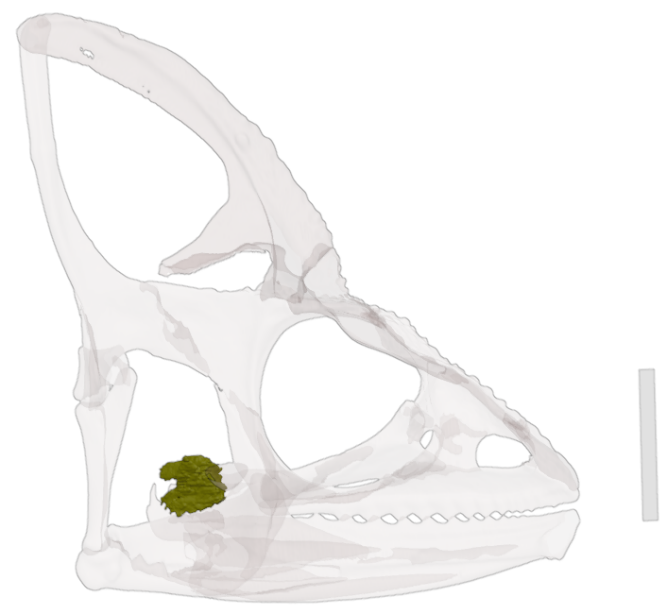


**Figure S13** – The protractor pterygoidei (PP) muscle in male (left) and female (right) *C. calyptratus* from a lateral (top) and medial (bottom) view. The grey scale bars beside each figure represents 10mm in length.


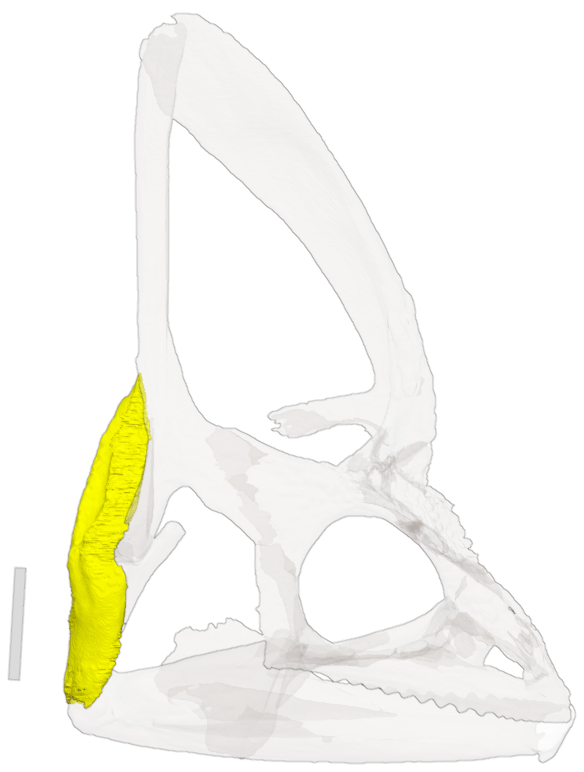

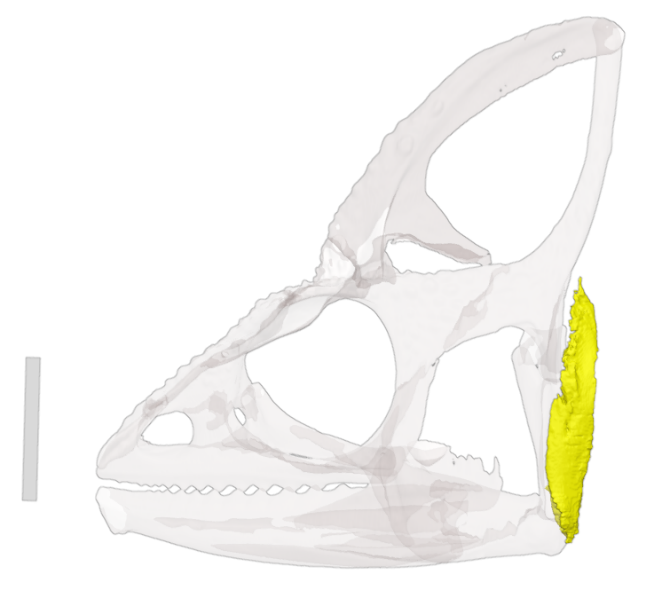


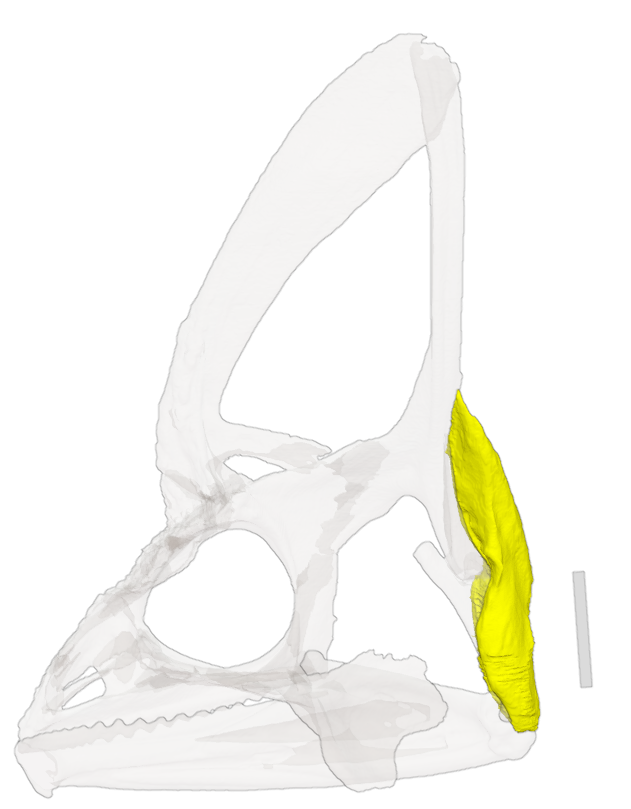

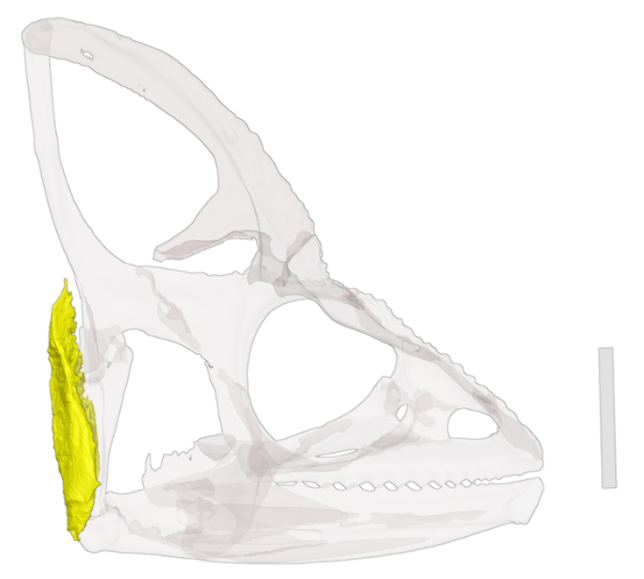


**Figure S14** – The depressor mandibulae in male (left) and female (right) *C. calyptratus* from a lateral (top) and medial (bottom) view. The grey scale bars beside each figure represents 10mm in length.


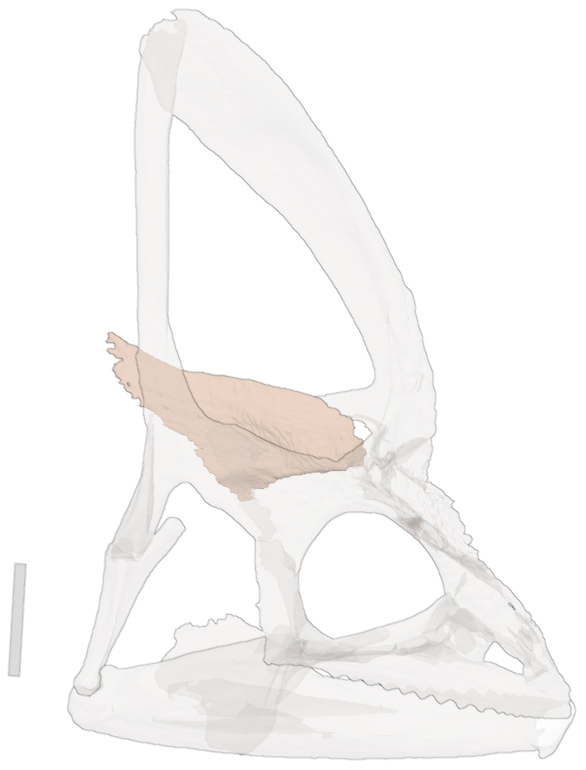

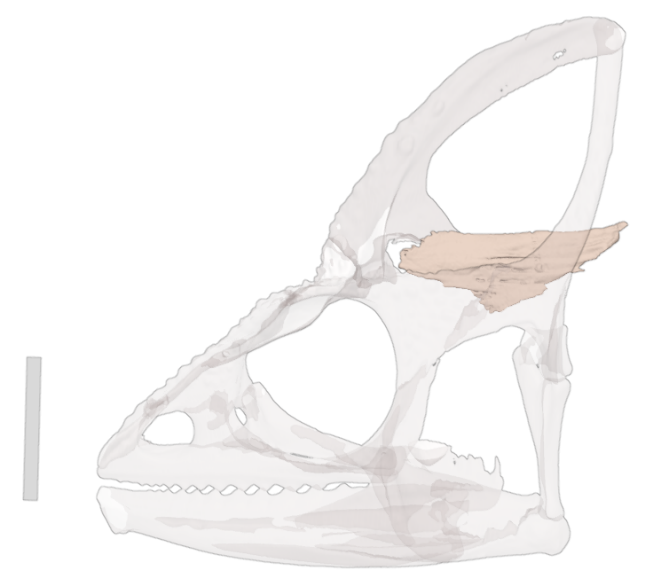

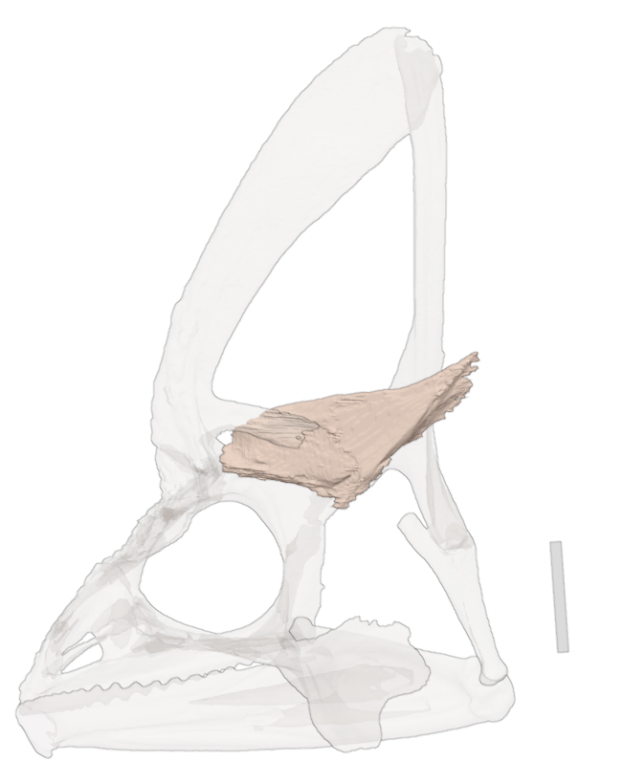

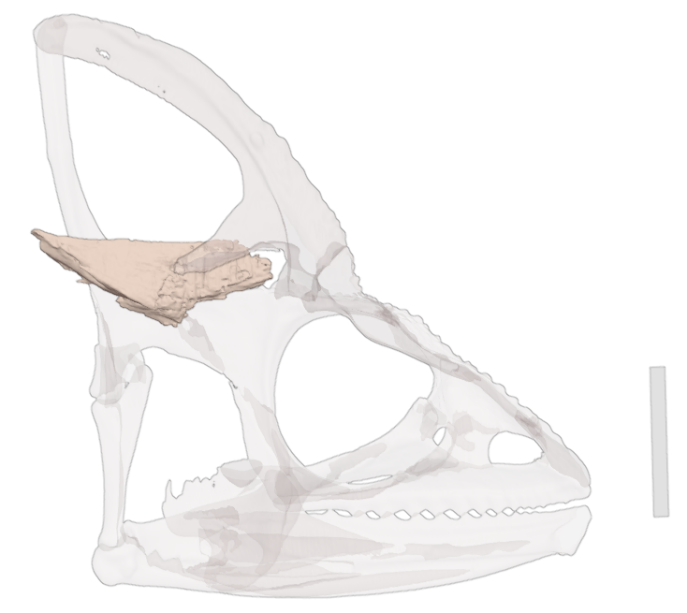


**Figure S15** – The longissimus capitis Pt 1 (LC-Pt1) muscle in male (left) and female (right) *C. calyptratus* from a lateral (top) and medial (bottom) view. The grey scale bars beside each figure represents 10mm in length.
