# Supplementary material for: 3D anatomical atlas of the heads of male and female adult Chamaeleo calyptratus
Source: Anat Rec (Hoboken). 2025 Nov 15;309(8):1937–69. doi: 10.1002/ar.70077 (PMC13331541; doi:10.1002/ar.70077)
Supplement: Supplementary file 2 — Data S2 Understanding comparative anatomy using 3D digital models. [file AR-309-1937-s002.pdf]

Understanding comparative anatomy using 3D digital models

Written as supplementary material for: Leavey, A., Gálvez-López, E., Herrel, A., Porro, L.B. (2025). 3D anatomical atlas of the head of male and female *Chamaeleo calyptratus. The Anatomical Record.*

Our open-access anatomical atlases of the veiled chameleon head provide a way for biological dissections to be carried out with just a computer and internet access, with the option to examine high-resolution versions of the models on free software. By making the modern technology currently being used in the anatomical sciences more accessible, we hope these atlases can be used to deepen students' understanding of various related topics across biology and to provide more equal learning opportunities across education. We hope that these digital models can be used in a hybrid approach that compliments physical dissection (e.g., as a revision tool, or to prepare/consolidate before/after a lab practical), to expand the range of species and anatomical structures that students can interact with. Therefore, we have designed two digital dissection lesson plans aligned with the UK science curriculum (one aimed at GCSE level students, one at first-year university undergraduates) to accompany our anatomical atlases. These lesson plans will guide educators through how to use digital models to encourage interactive learning of anatomy and facilitate the development of many practical skills that are relevant to modern scientific research. They are designed to bridge the gap between theoretical knowledge in comparative anatomy and hands-on analysis of real-world examples, fostering deeper understanding of how evolutionary pressures shape the anatomy of living organisms.

Lesson Plan 1

Target audience: Secondary School Students (Ages 14–16, KS4 / GCSE level)
Duration: 60 minutes

Learning Objectives:

By the end of this lesson, students should be able to:

1. **Understand the concept of comparative anatomy** and how it helps us understand evolutionary relationships and adaptations in different species.
2. **Identify and describe key differences** between male and female chameleon heads.
3. **Interpret anatomical data** to draw conclusions about the functional differences between male and female chameleons.
4. **Apply knowledge of anatomy** to evaluate how physical features are related to environmental and biological needs.

Links to the UK National Curriculum Links for KS4 Biology (GCSE Level):

- **Biology:**
    - Comparative anatomy is a way of visualising evolutionary adaptations (e.g., it can be linked to how species have changed in response to their environments).

    - Understanding the structure of animal systems, including how skulls adapt to feeding and sensory needs, and how this can differ between the sexes.
- **Scientific Enquiry Skills:**
    - Use of modern technology for scientific observation and investigation.
    - Collecting and interpreting data by using a range of methods.

Materials Needed:

- Computers with internet access.
- 3D digital models of male and female chameleon skulls
    - Available to view online (https://sketchfab.com/aleavey/collections)
    - Available to download in advance from Figshare (https://figshare.com/projects/3D_anatomical_atlas_of_the_heads_of_male_and_female_adult_Chamaeleo_calyptratus/263071) for visualisation in most glTF file viewers (e.g., https://gltf-viewer.donmccurdy.com/).
    - To have the ability to visualise anatomical labels and toggle on/off each element of the anatomy, Blender files are also available to download from Figshare (created in Version 4.2 but compatible with most other versions).
- Projector (for teacher-led explanation and demonstration).
- Writing materials for students to record their observations.

Preparation before the lesson:

- If students have already been introduced to the concepts of **comparative anatomy, sexual dimorphism,** and **evolution,** the educator can recommend that the students review this lesson material.
- The educator should familiarise themselves with the platform that they would like the students to view the digital atlases on - SketchFab or Blender. There are many online tutorials to facilitate this (e.g., https://www.youtube.com/watch?v=sxTIRE6c6bo&t=65s). The educator may also share this information with the students so that they can get used to the platform controls ahead of the lesson.
- The educator could instruct the students to do some research on the life of the veiled chameleon, *Chamaeleo calyptratus*, as this will help them to make inferences about how anatomy is related to ecology and function in this lesson.

Lesson structure:

Introduction (10 minutes):

- The educator should briefly introduce the concept of **comparative anatomy**, explaining how studying the anatomical differences between species helps us understand evolution and adaptation.

- Discuss the concept of **sexual dimorphism** - the differences between male and female individuals of the same species - and how it can be observed in skeletal structures.
- Introduce **the veiled chameleon** as a case study. This species has one of the largest cranial crests out of all chameleon species – studies have shown that a larger crest may be linked to larger bite forces.
- Explain that today, students be examining digital versions of male and female chameleon heads to identify anatomical differences that could be related to specific behaviours, such as territoriality, feeding, or mating.

Activity 1: Introduction to the 3D models via teacher-led demonstration (10 minutes):

- Using the projector, show students how to navigate through the 3D digital models of the male and female chameleon skulls on the platform.
- Demonstrate how to rotate, zoom, and interact with the models, especially how to visualise different materials (turning the tick-marks on and off).
- Highlight key areas of the skulls such as the jaw, teeth, eye sockets, and cranial crest.

Activity 2: Independent examination of the models by students (25 minutes):

- In pairs or small groups, students will use the computers or tablets to explore the 3D digital models.
    - If using SketchFab.com to view the models, one website tab should be displaying open male model, while another should show the female.
    - If using Blender, two separate sessions will need to be opened to see the male and female model. If this causes the computer to lag, then small groups of students could share two computers, one displaying the male model and one showing the female.
- They should start with just the tick-mark for the collection 'BONES' selected so only the skeletal anatomy is visible.
- Allow time for students to zoom in and examine specific areas of the skull in detail and answer question 1.
- Next, students should toggle off the 'BONES' collection and instead visualise the 'SOFT TISSUE' collection. Allow them time to explore the muscles and the brain that are now visible.
- Students answer the following questions throughout the activity:

1. **What are the key differences between the male and female chameleon skulls?** Examine the size of the crest, size of the jaw, overall shape of the skull, presence of distinct features like bony protrusions, and tooth structures.

   Males have a larger cranial crest which is oriented more vertically.

2. **What might these differences suggest about the muscles that are attached to the skull?**

   A larger cranial crest allows space for larger muscles. These muscles result in a stronger bite.

3. **What might these differences suggest about the roles or behaviours of each sex?**

   Males compete for females and are highly territorial, so they require larger bite forces for male-male competition. The size of the cranial crest is a signal to females that they are strong fighters. Students may also suggest that males eat larger/harder prey, but previous studies in other chameleon species have shown that this is not the case.

Activity 3: Group discussion (10 minutes):

- Invite students to share their findings and insights with the class.
- Invite students to make connections between these findings and **adaptation**, emphasizing how evolution shapes the body structures of organisms to meet environmental demands.

Conclusion and reflection (5 minutes):

- The educator should recap the key learning points of the lesson, focusing on the connection between anatomy and function.
- Ask students to reflect on how the use of 3D models helped them understand the concept of comparative anatomy in a more interactive way.
- For homework or as an extension activity, students can research other species that exhibit sexual dimorphism and consider the evolutionary advantages of these differences. There are many models of other reptile skulls available on SketchFab.com.

Formative assessment suggestion:

- Observing students during the practical activity to gauge their engagement and understanding of the material.
- Reviewing what students have written regarding the differences between male and female chameleon skulls, and how students explain their observations.

Summative assessment suggestion:

- At the end of the unit, students could write a short report or give a presentation on the comparative anatomy in another species, referencing the techniques used in this lesson.

Support for struggling students:

- Provide worksheets with prompts to guide their observations.
- Offer a brief one-on-one support session during the practical to help students navigate the 3D models.
- Pair students with higher-level peers for collaborative learning and mutual support.

Extension for advanced students:

- Challenge students to consider how the chameleon’s anatomy compares to other species of reptiles.

- Ask students to hypothesize how these anatomical differences would affect the species' survival strategies in various environments.

Lesson Plan 2

Target Audience: First-year university undergraduate students (Biology or Zoology)
Duration: 90 minutes

Learning Objectives:

By the end of this lesson, students should be able to:

1. **Understand the principles of comparative anatomy** and its application in studying evolutionary adaptations and functional morphology.
2. **Analyse and compare** the anatomical features of male and female chameleon skulls using 3D digital models.
3. **Critically assess** the implications of observed anatomical differences in relation to evolutionary pressures, behavioural ecology, and sexual dimorphism.
4. **Conduct virtual dissections** to apply knowledge of digital tools for anatomical analysis and explore functional differences in animal morphology.

Curriculum Links:

- **Biology / Zoology Undergraduate Level (1st Year):**
  - **Functional Morphology:** Explore the relationship between form and function in animal species, with a focus on skull structure and sexual dimorphism.
  - **Evolutionary Biology:** Examine how anatomical features are shaped by evolutionary pressures and environmental factors.
  - **Comparative Anatomy and Dissection Techniques:** Use of modern digital tools in place of traditional dissections to examine anatomical differences.
- **Skills Development:**
  - **Data Collection and Analysis:** Students will engage with 3D models, noting anatomical variations and formulating hypotheses based on their observations.
  - **Critical Thinking and Discussion:** Engage in group discussions to evaluate evolutionary implications of observed anatomical differences.

Materials Needed:

- Computers with internet access.
- 3D digital models of male and female chameleon skulls
  - Available to view online (https://sketchfab.com/aleavey/collections)
  - Available to download in advance from Figshare (https://figshare.com/projects/3D_anatomical_atlas_of_the_heads_of_mal

e_and_female_adult_Chamaeleo_calyptratus/263071) for visualisation in most glTF file viewers (e.g., https://gltf-viewer.donmccurdy.com/).
    - To have the ability to visualise anatomical labels and toggle on/off each element of the anatomy, Blender files are also available to download from Figshare (created in Version 4.2 but compatible with most other versions).
- Projector (for teacher-led explanation and demonstration).
- Writing materials for students to record their observations.

Preparation before the lesson:

- If students have already been introduced to the concepts of **comparative anatomy, sexual dimorphism,** and **evolution,** the educator can recommend that the students review this lesson material.
- The educator should familiarise themselves with the platform that they would like the students to view the digital atlases on - SketchFab or Blender. There are many online tutorials to facilitate this (e.g., https://www.youtube.com/watch?v=sxTIRE6c6bo&t=65s). The educator may also share this information with the students so that they can get used to the platform controls ahead of the lesson.
- The educator could instruct the students to do some research on the life of the veiled chameleon, *Chamaeleo calyptratus*, as this will help them to make inferences about how anatomy is related to ecology and function in this lesson.

Lesson Structure:

Introduction (15 minutes):

1. **Instructor Presentation:**

- Begin by introducing the core principles of **comparative anatomy**—the study of similarities and differences in the anatomy of different species—and its role in understanding evolutionary relationships, adaptation, and specialization.
- Explain the concept of **sexual dimorphism**, focusing on how male and female individuals of the same species may differ in morphology due to reproductive roles, behaviour, and ecological pressures.
- Introduce the **veiled chameleon** as a model organism – it has many unique evolutionary traits, including one of the largest cranial crests out of all chameleon species. Studies have shown that a larger crest is linked to larger bite forces.
- Explain that today, students will examine male and female chameleon skulls to identify anatomical differences that could be related to specific behaviours, such as territoriality, feeding behaviour, territoriality, or mating.

2. **Contextualizing the Practical:**

- Explain how digital models and virtual dissections have become increasingly important in modern biology and zoology, especially as alternatives to traditional dissections, and how they can aid in the study of anatomical differences in living species without causing harm.

Activity 1: Introduction to 3D models via educator-led demonstration (15 minutes):

- Using the projector, show students how to navigate through the 3D digital models of the male and female chameleon skulls on the platform.
- Demonstrate how to rotate, zoom, and interact with the models, especially how to visualise different materials (turning the tick-marks on and off).
- Highlight key areas of the skulls such as the jaw, teeth, eye sockets, and cranial crest.

Activity 2: Independent exploration and analysis (40 minutes):

- In pairs or small groups, students will use the computers or tablets to explore the 3D digital models.
  - If using SketchFab.com to view the models, one website tab should be displaying open male model, while another should show the female.
  - If using Blender, two separate sessions will need to be opened to see the male and female model. If this causes the computer to lag, then small groups of students could share two computers, one displaying the male model and one showing the female.
- They should start with just the tick-mark for the collection 'BONES' selected so only the skeletal anatomy is visible.
- Allow time for students to zoom in and examine specific areas of the skull in detail.
- They will examine the skulls in detail, making observations and taking notes on the following key areas:
  - **Anatomical Differences:** Identify any notable differences between the male and female chameleon skulls (e.g., size, shape, features like protrusions or differences in the mandible).
  - **Functional Implications:** Hypothesize how these differences might be linked to the size and shape of the muscles (without looking at the soft tissue). Students should note how they think this might impact the chameleon's feeding strategies, mating behaviour, or territorial roles.
  - **Sexual Dimorphism:** Consider the potential evolutionary reasons for these differences—what role does sexual selection or ecological pressures play in shaping these traits?
- Next, students should toggle off the 'BONES' collection and instead visualise the 'SOFT TISSUE' collection. Allow them time to explore the muscles that are now visible.
- Students should document whether the muscles meet their expectations based on what they noted about the skull. How do they differ between the sexes? How might differences in these jaw-closing muscles impact function?

Activity 3: Group discussion and critical analysis (15 minutes):

- The educator could facilitate a class discussion where each group shares their observations and analyses. Encourage students to think critically about the functional significance of the anatomical features they've identified.
- Pose higher-level questions, such as:
  - How do the differences between male and female skulls reflect different ecological or reproductive strategies?
  - What does the study of comparative anatomy tell us about the evolution of behavioural traits?
  - Are there parallels to be drawn with sexual dimorphism in other reptile species or animals in general?

Conclusion and Reflection (5 minutes):

- Summarize the key findings from the student discussions, highlighting how anatomical differences relate to ecological and behavioural adaptations in chameleons.
- Reflect on how the use of 3D digital models enhances the study of comparative anatomy and allows for more interactive, hands-on learning with fewer ethical implications.
- Provide a preview of future topics, such as functional morphology in other vertebrates and the evolution of different feeding strategies, which will build on this foundational knowledge.

Formative assessment suggestion:

- Review student worksheets to assess their ability to identify and analyse anatomical differences between male and female chameleon skulls.
- Observe group discussions for engagement and depth of critical thinking.
- Provide immediate feedback on students' analyses during the class discussion.

Summative assessment suggestion:

- As a follow-up, assign students to research and write a report comparing the skull morphology of chameleons with that of another reptile or vertebrate species, considering sexual dimorphism and evolutionary adaptations. There are many digital anatomical models available on websites such as SketchFab.com. The report should integrate findings from this practical with broader comparative anatomy concepts.

Support for struggling students:

- Provide additional guidance during the practical session to help students navigate the 3D models effectively.
- Offer prompts or scaffolding questions to help students make connections between anatomy and function.

Extension for advanced students:

- Encourage advanced students to research more information about the veiled chameleon and other *Chamaeleo* species to inform their discussions of what could be driving this extreme expansion of the cranial casque.
- The anatomical atlas also includes the cranial muscles – ask students to explore the relation between skeletal and muscular anatomy. How does the enlarged cranial crest impact the shape and size of the jaw adductor muscles?
- Ask students to explore and compare anatomical differences across other species with similar ecological roles, such as other lizards or reptiles.
